# Supplementary figures and images for: Imputed genomes and haplotype-based analyses of the Picts of early medieval Scotland reveal fine-scale relatedness between Iron Age, early medieval and the modern people of the UK
Source: PLoS Genet. 2023 Apr 27;19(4):e1010360. doi: 10.1371/journal.pgen.1010360 (PMC10138790; doi:10.1371/journal.pgen.1010360)

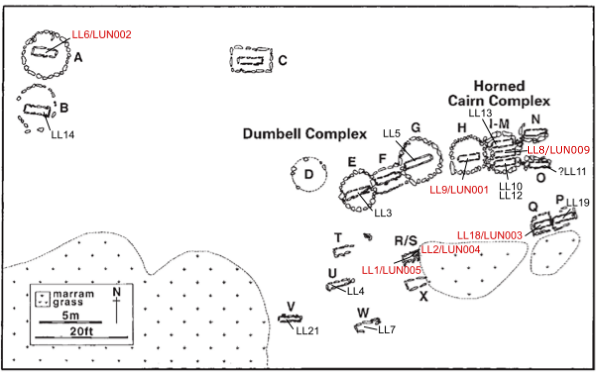

Supplement: S1 Fig — The plan was published in Greig et al. [36]. The samples yielding DNA are in red. (TIF) [file pgen.1010360.s013.tif]

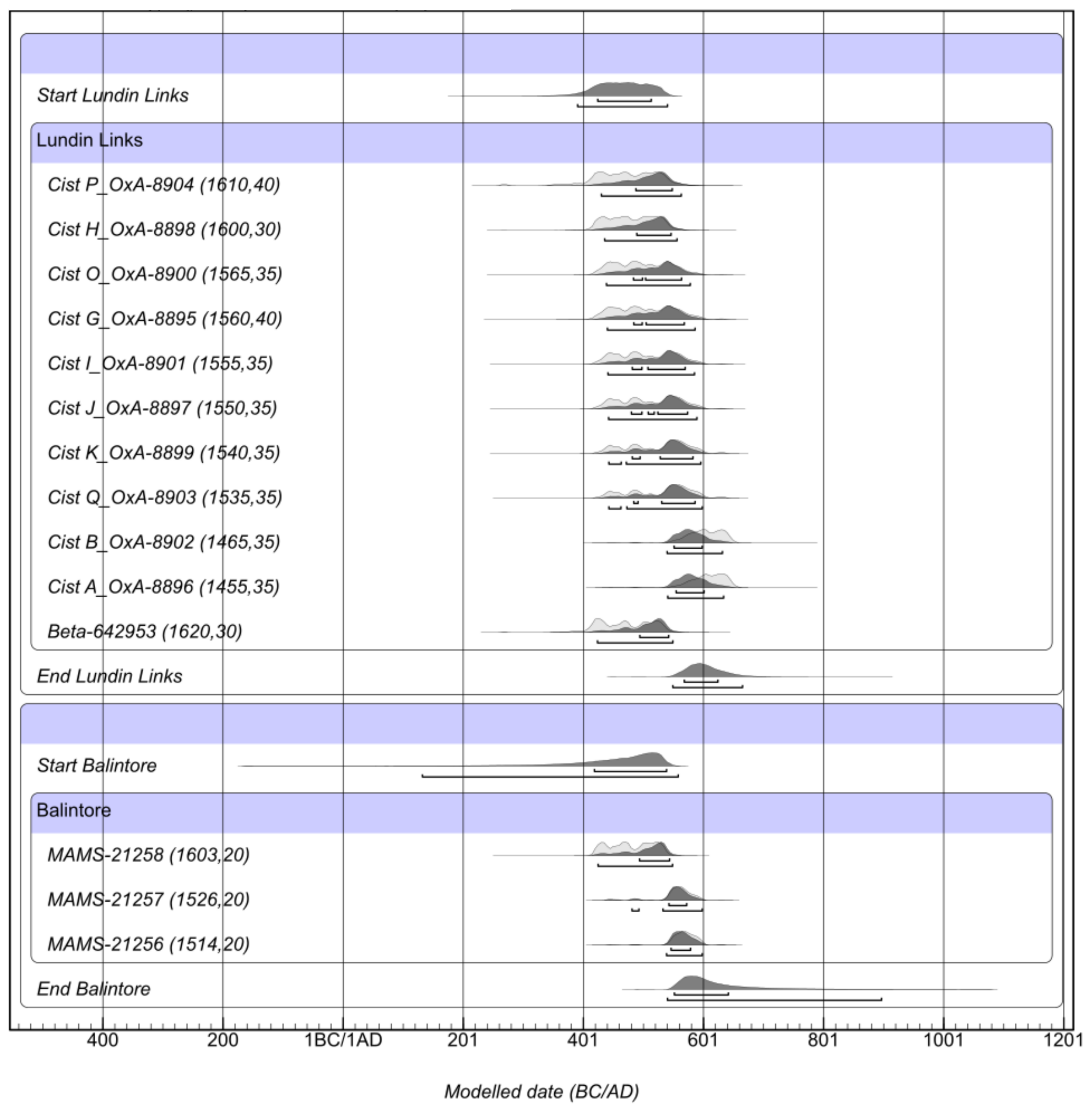

Supplement: S2 Fig — Radiocarbon determinations calibrated using OxCal. v4.4 [95] and the IntCal20 atmospheric curve [96]. (PNG) [file pgen.1010360.s014.png]

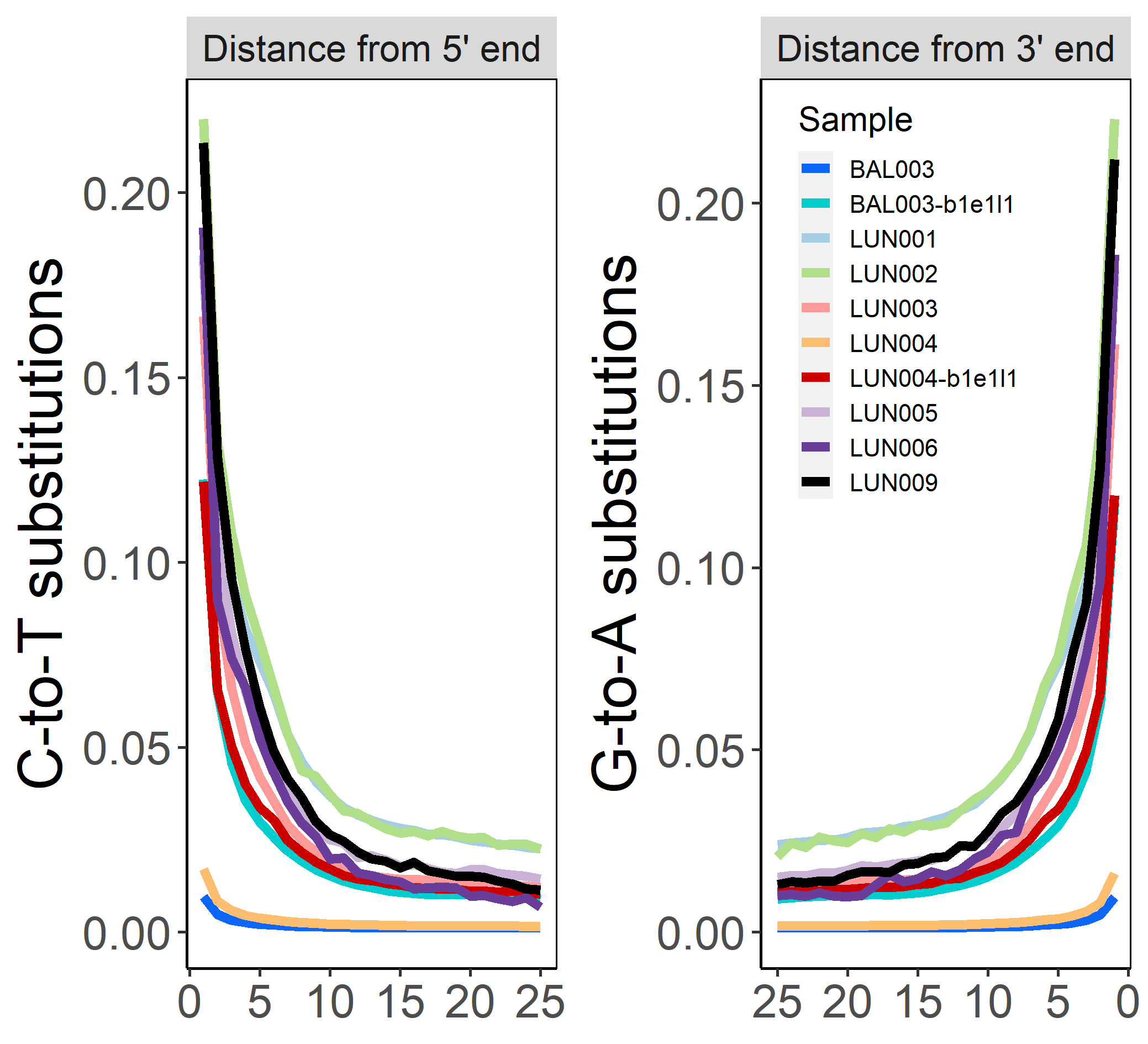

Supplement: S3 Fig — (TIF) [file pgen.1010360.s015.tif]

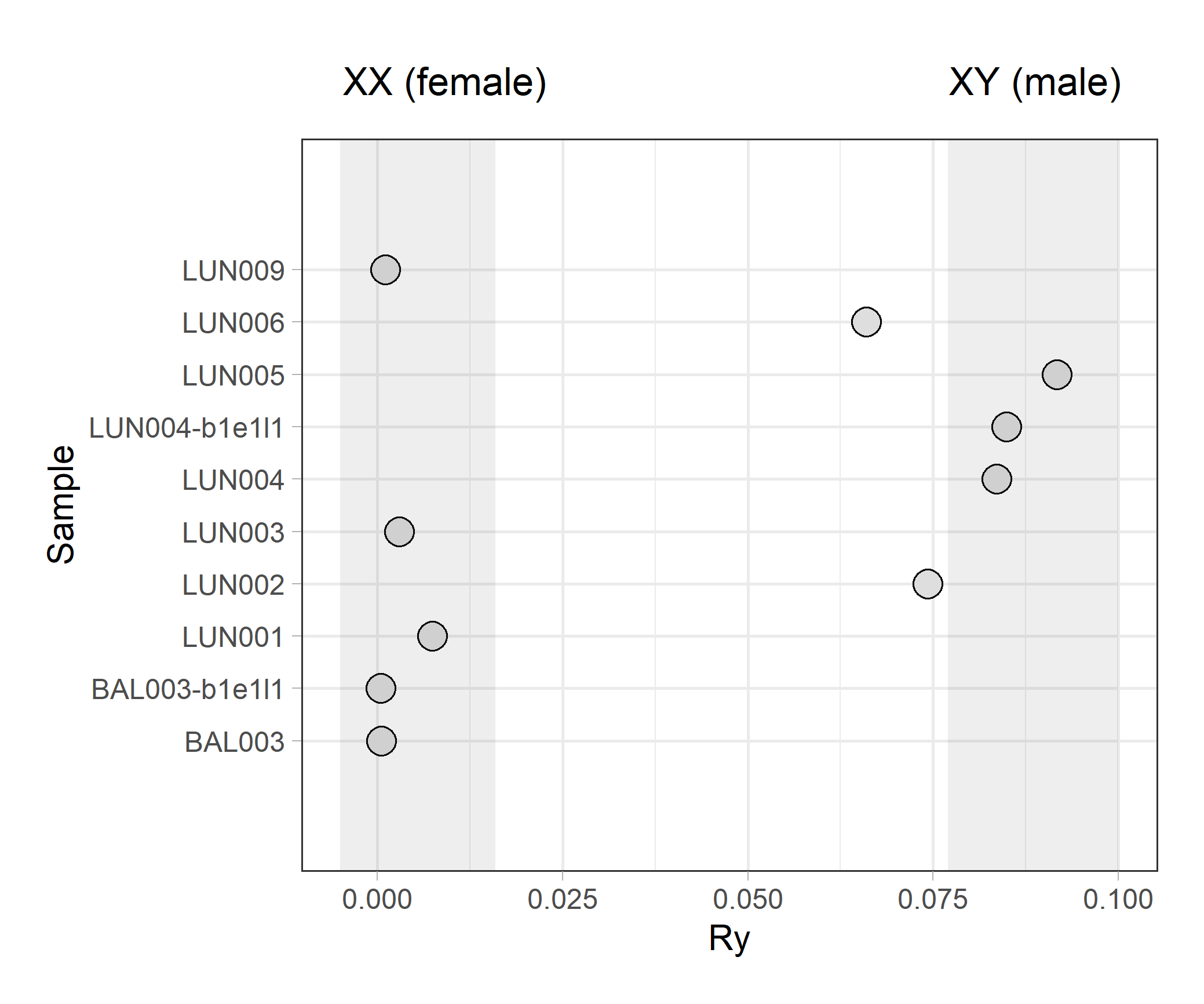

Supplement: S4 Fig — We determined the biological sex based on Y-chromosome alignments (ny) compared to the total number of reads of the X and Y-chromosomes (nx + ny). The 95% confidence interval (CI) was computed as Ry ±(1.96 x Ry x (1 –Ry))/((ny + nx)). (TIF) [file pgen.1010360.s016.tif]

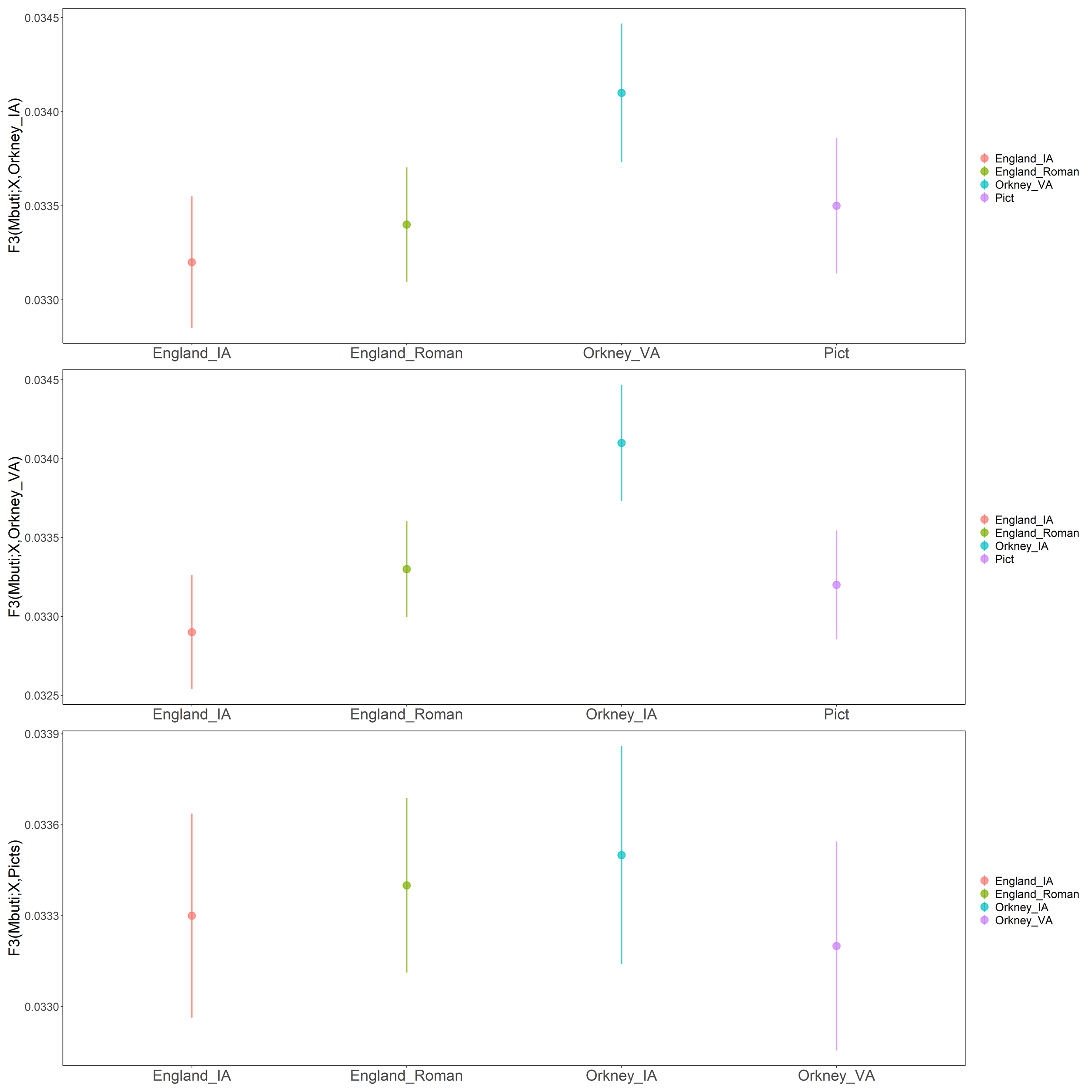

Supplement: S5 Fig — (TIF) [file pgen.1010360.s017.tif]

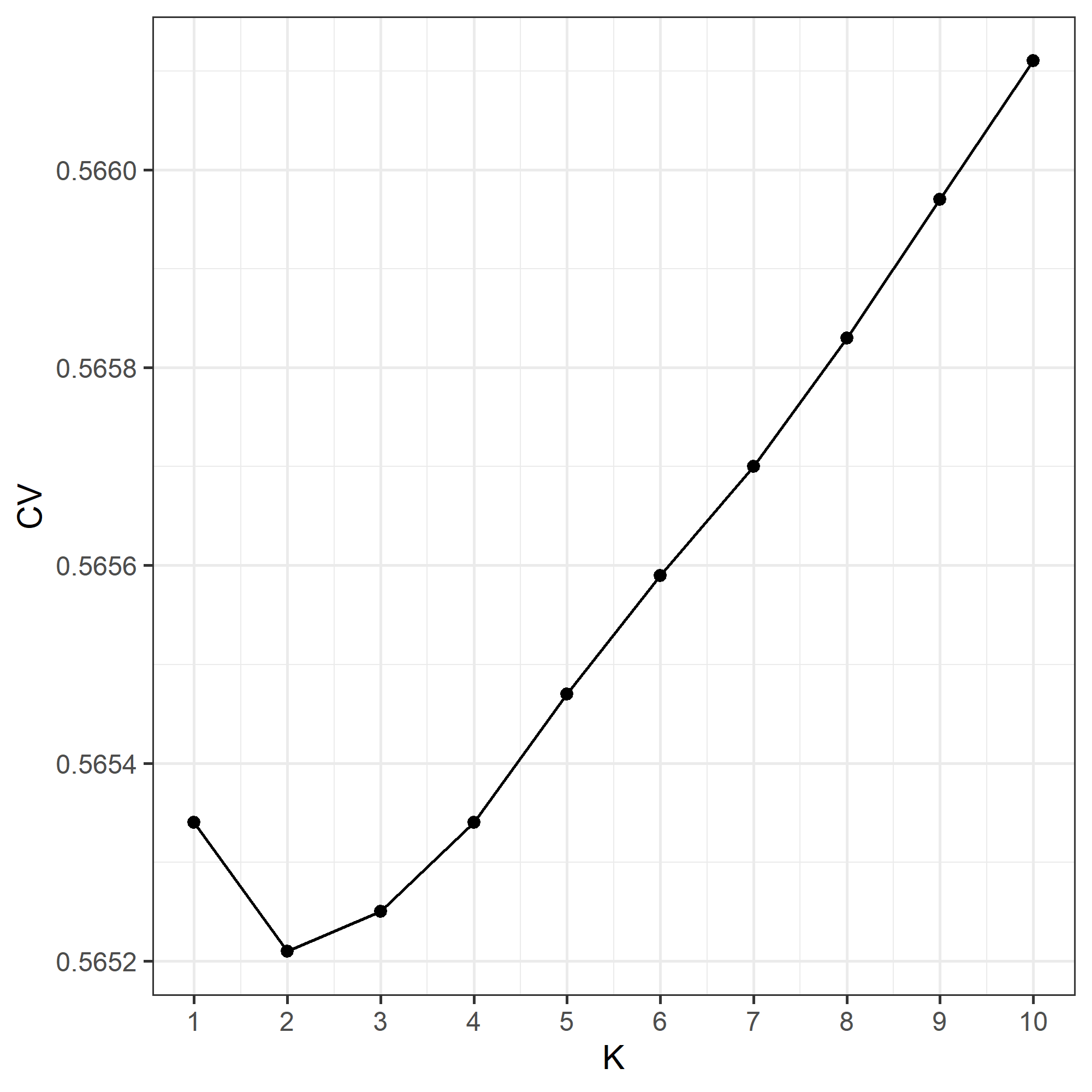

Supplement: S6 Fig — The cross-validation was done using the—cv option of admixture runs for K = 1 to K = 10 with 4,914 individuals and 87,518 SNPs using 50 bootstraps (-B 50). (TIF) [file pgen.1010360.s018.tif]

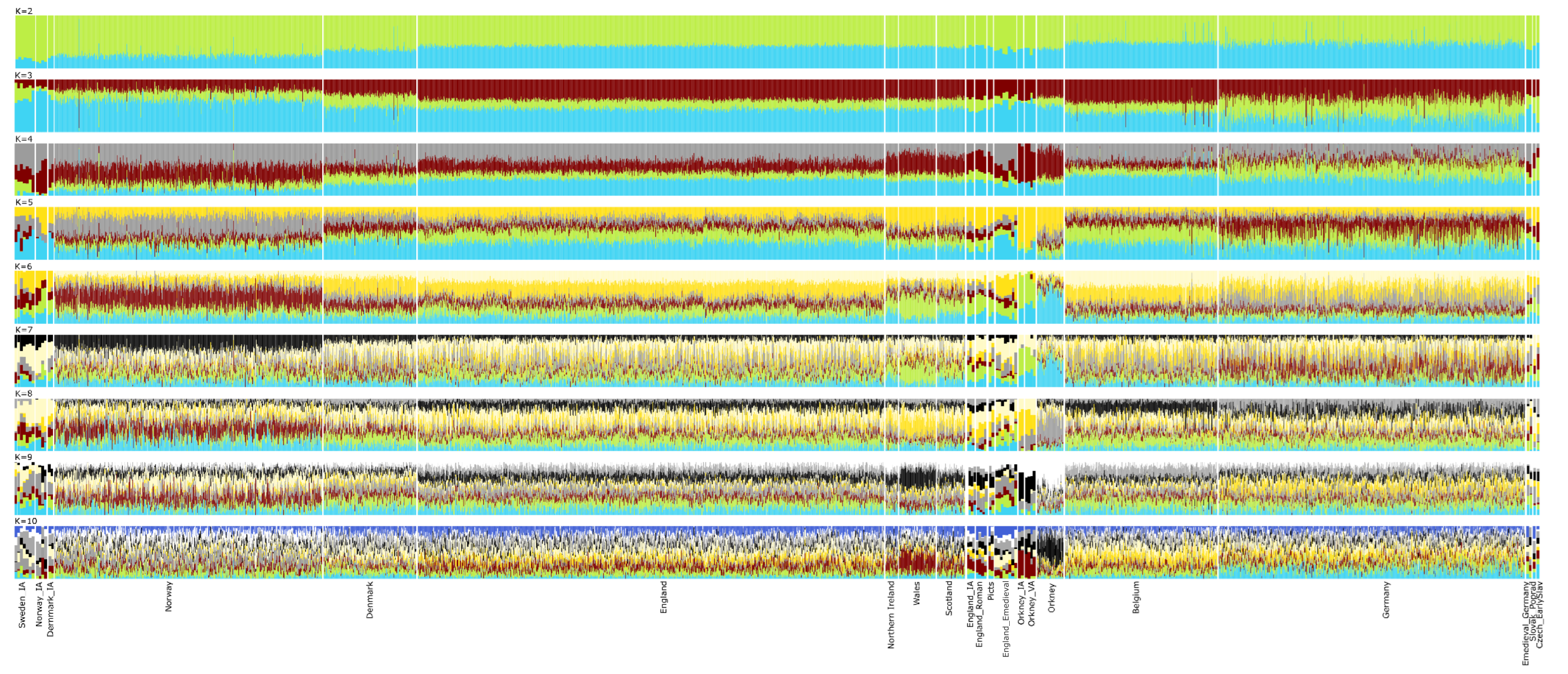

Supplement: S7 Fig — (TIF) [file pgen.1010360.s019.tif]

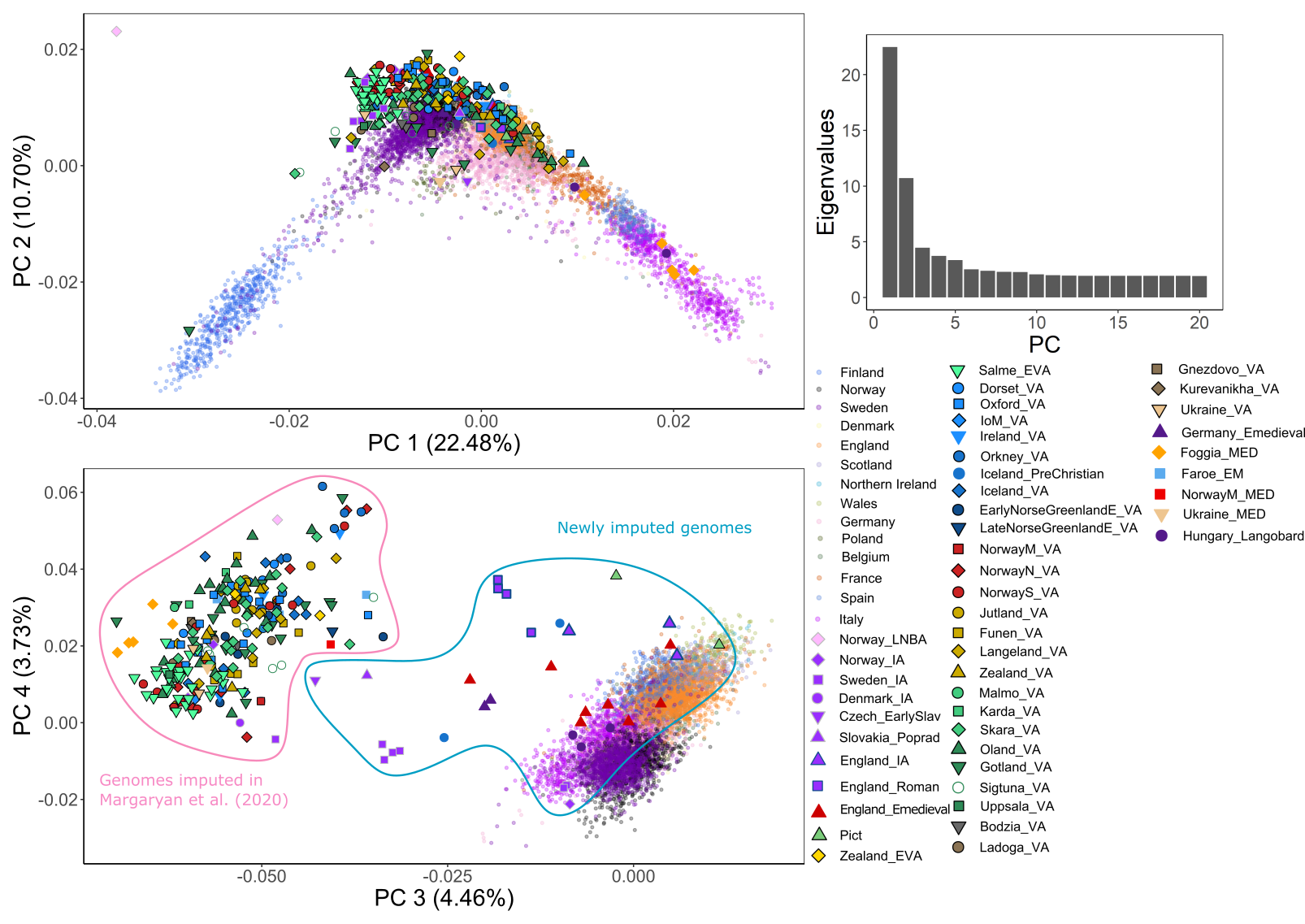

Supplement: S8 Fig — SNPs with maf <5% were removed and pruned (88,040 SNPs remained). PC3 is impacted by allele frequency bias differentiating the data imputed in Margaryan et al. [9] and the genomes newly imputed in this study. (TIF) [file pgen.1010360.s020.tif]

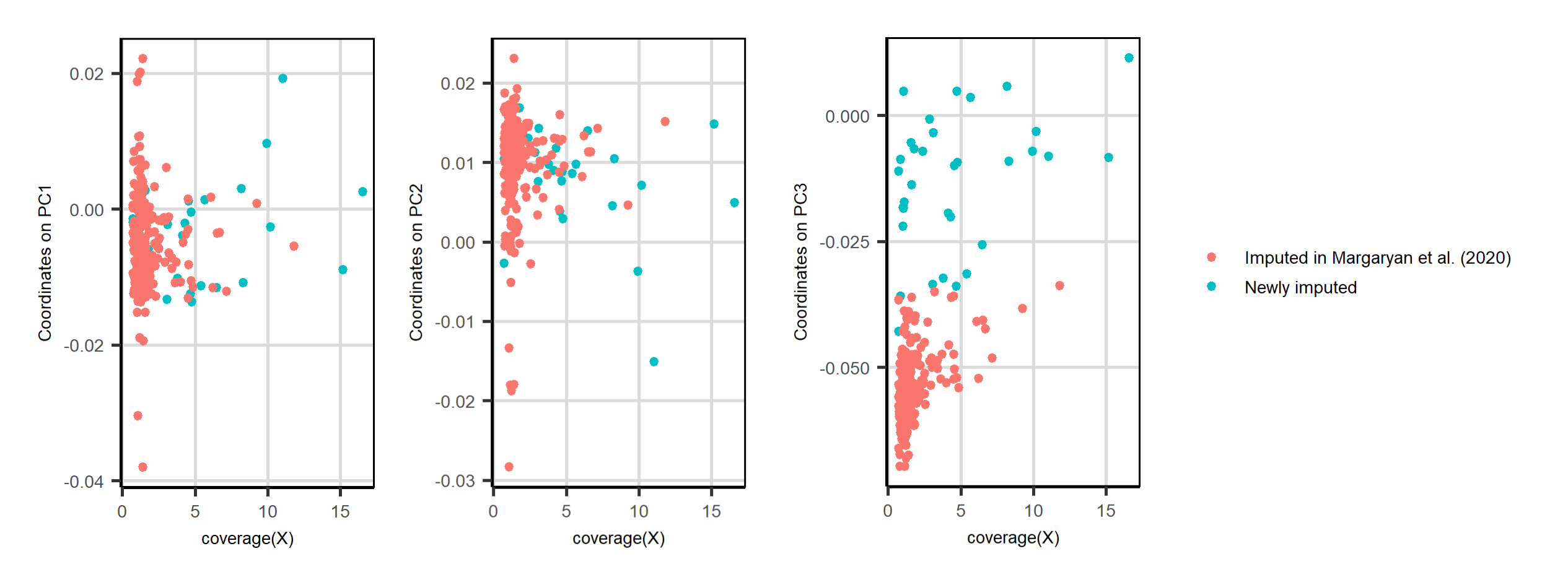

Supplement: S9 Fig — The genomes newly imputed in this study are in blue and the genomes imputed in Margaryan et al. [9] are in pink. (TIFF) [file pgen.1010360.s021.tiff]

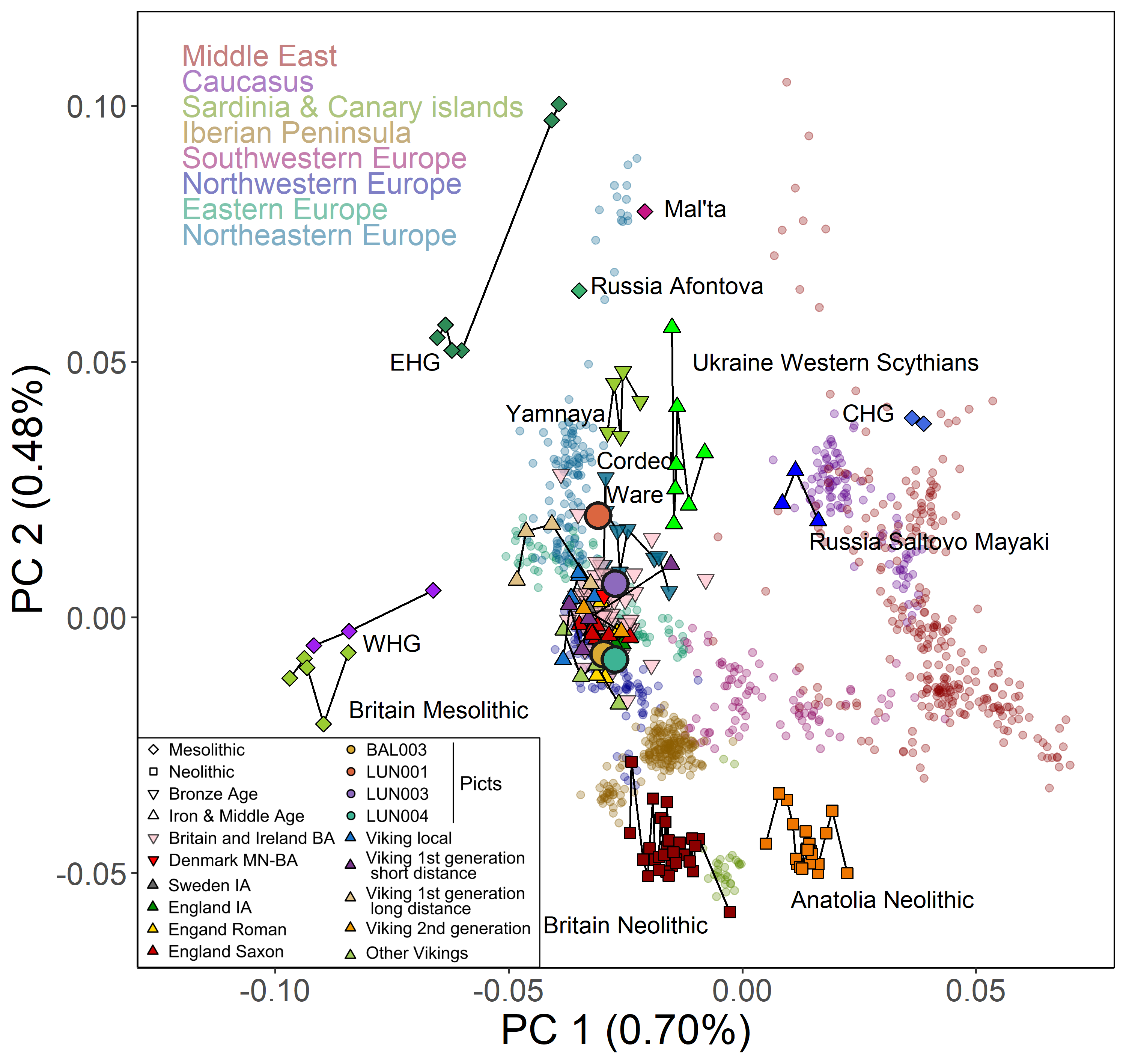

Supplement: S10 Fig — Pseudo-haploid genotypes from ancient samples are projected onto the first two principal components defined by 1,056 present-day West Eurasians from the ‘HO’ dataset (S). VA, Viking Age. (TIFF) [file pgen.1010360.s022.tiff]

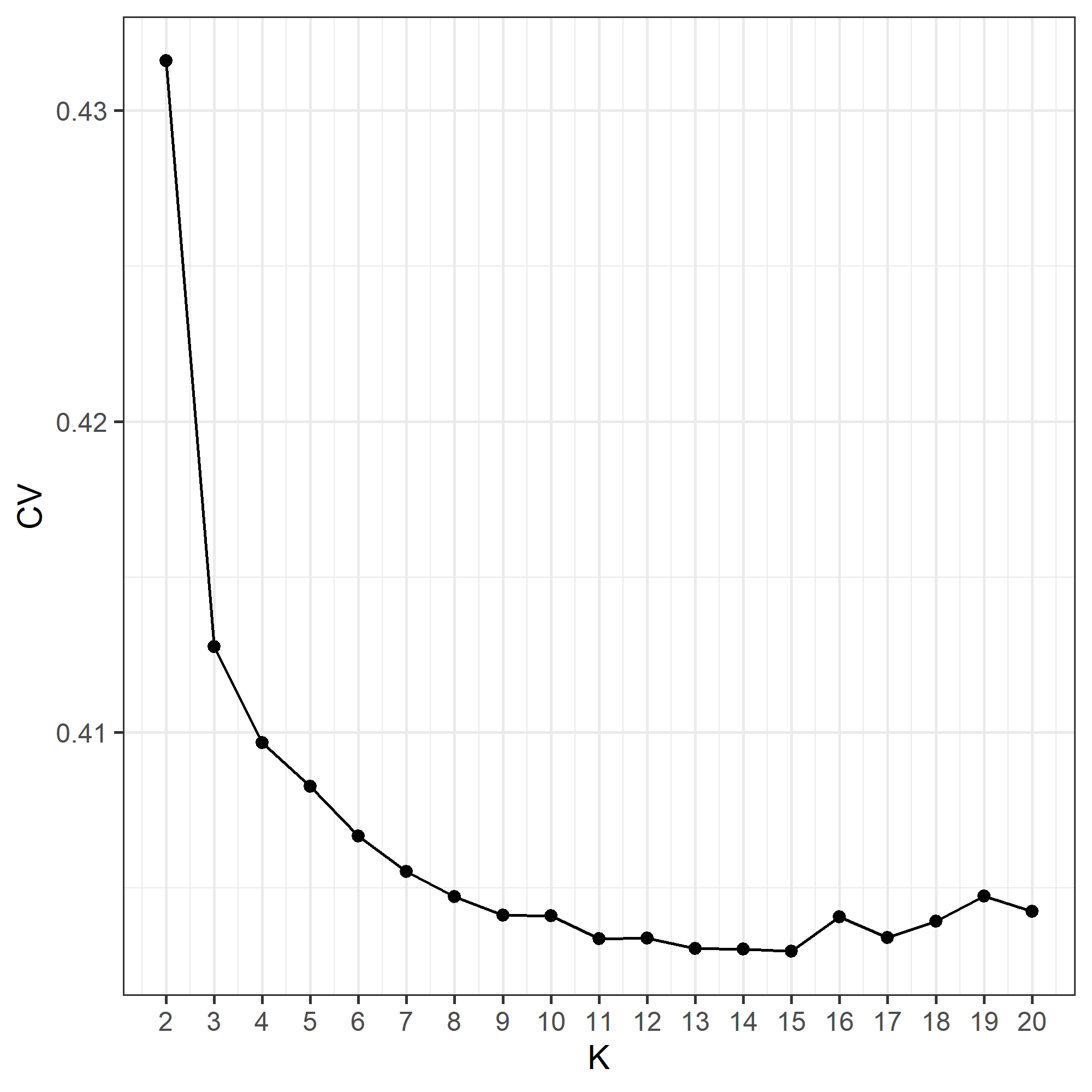

Supplement: S11 Fig — The cross-validation was done using the—cv option of admixture runs for K = 3 to K = 20 with 3,591 individuals and 85,655 markers. (TIF) [file pgen.1010360.s023.tif]

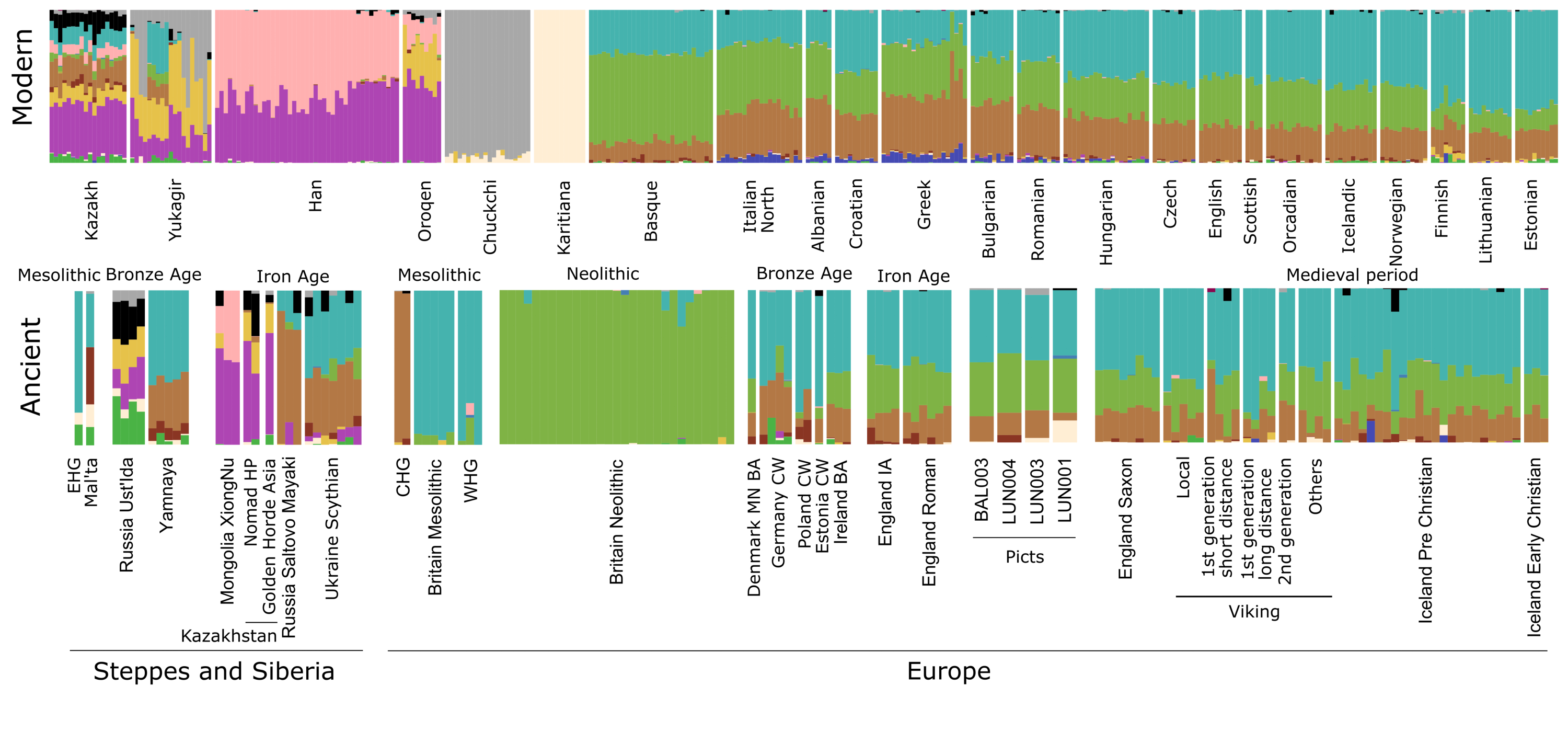

Supplement: S12 Fig — Full displays of the ADMIXTURE analysis are in S13–S15 Figs. (TIFF) [file pgen.1010360.s024.tiff]

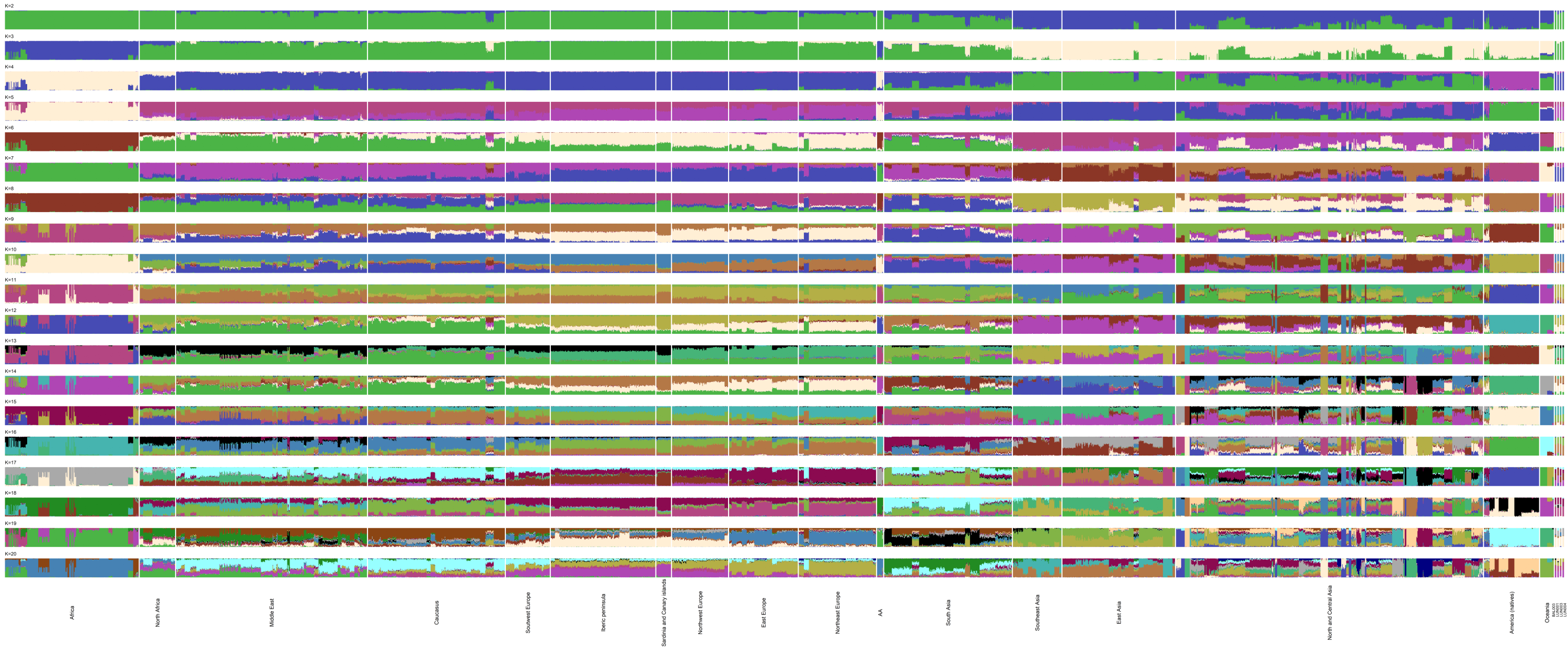

Supplement: S13 Fig — Only modern individuals are represented. (TIFF) [file pgen.1010360.s025.tiff]

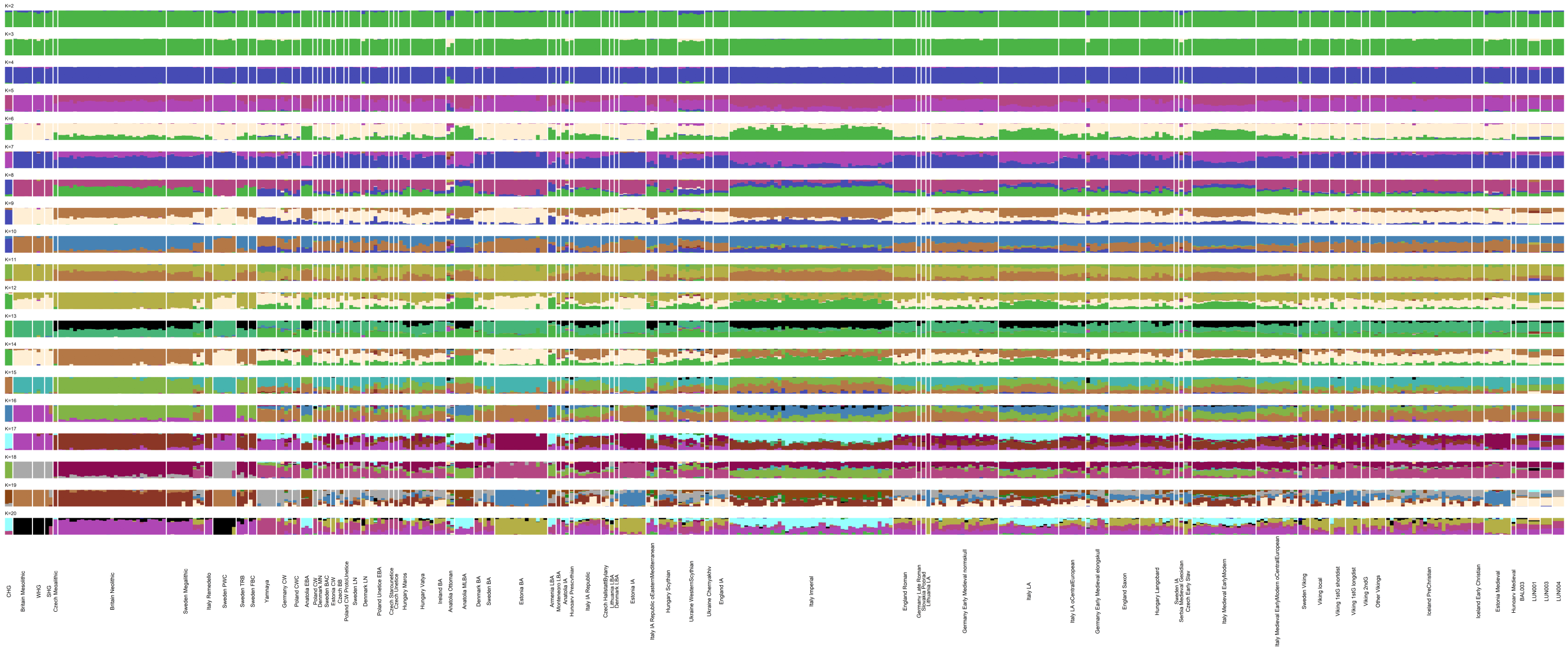

Supplement: S14 Fig — Only ancient individuals from western Eurasia are represented. (TIFF) [file pgen.1010360.s026.tiff]

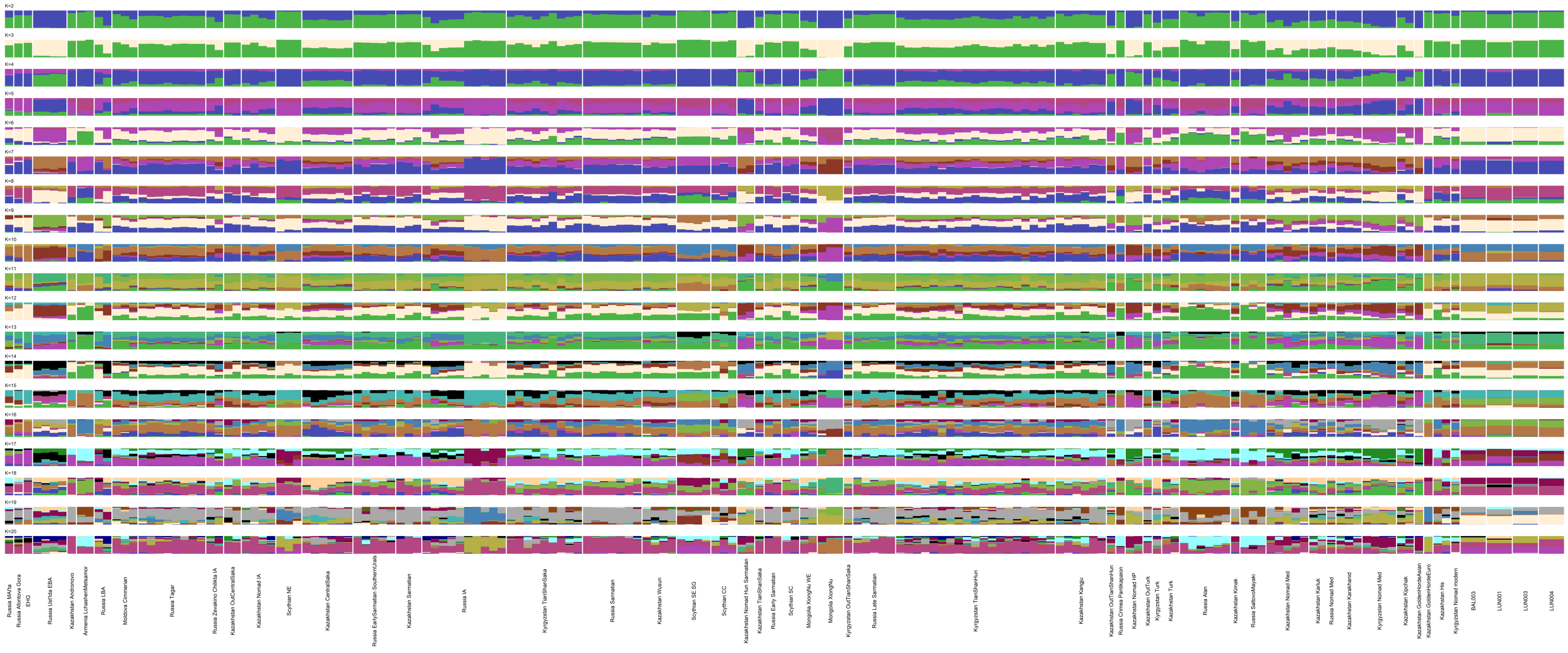

Supplement: S15 Fig — Only ancient individuals from Eastern Eurasia are represented. (TIFF) [file pgen.1010360.s027.tiff]

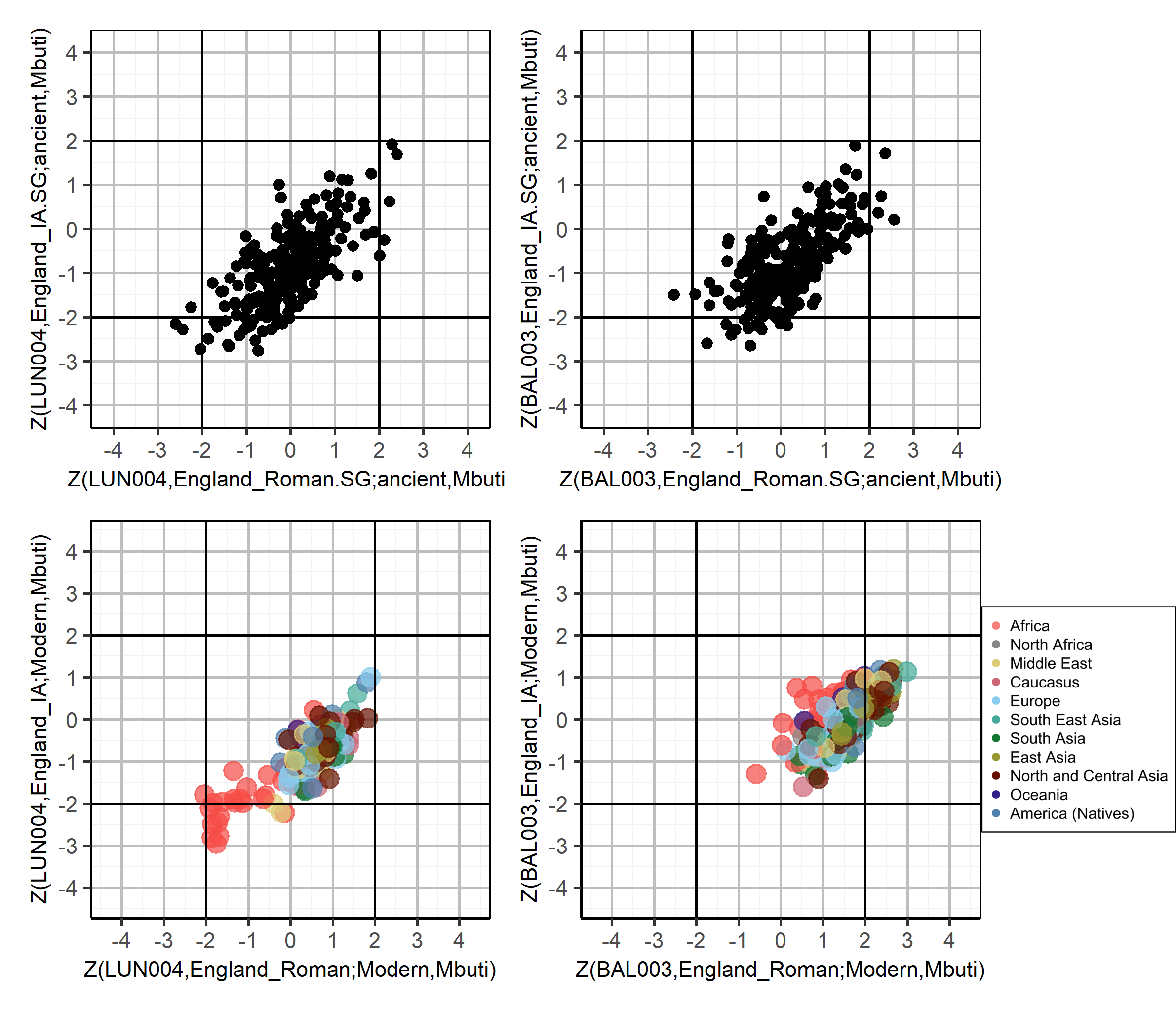

Supplement: S16 Fig — We tested for symmetry as D(Pict, ancient England; X, Mbuti) and plotted the resulting Z-scores. Details on the test results and sample size are in S10 Table. (TIF) [file pgen.1010360.s028.tif]

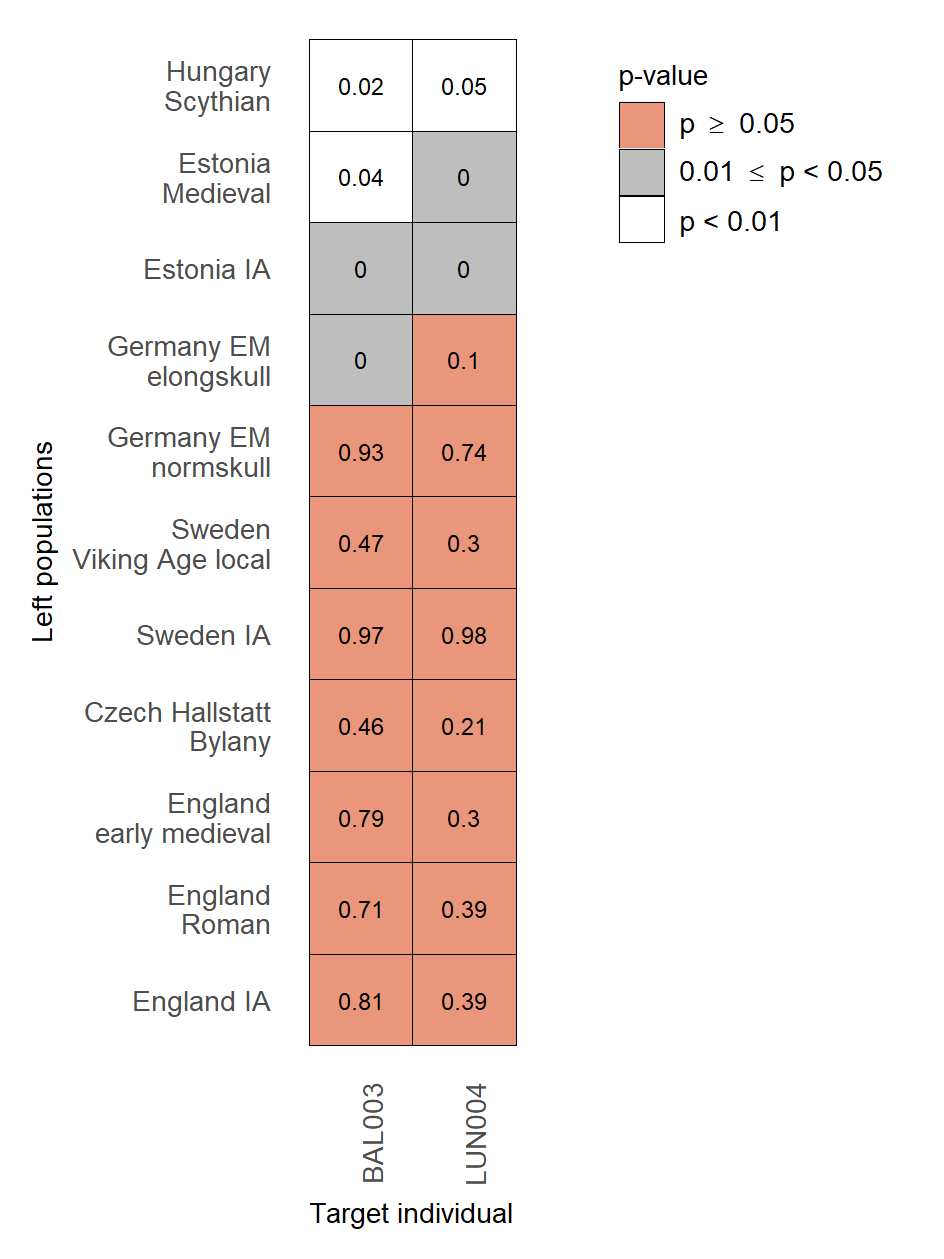

Supplement: S17 Fig — (TIF) [file pgen.1010360.s029.tif]

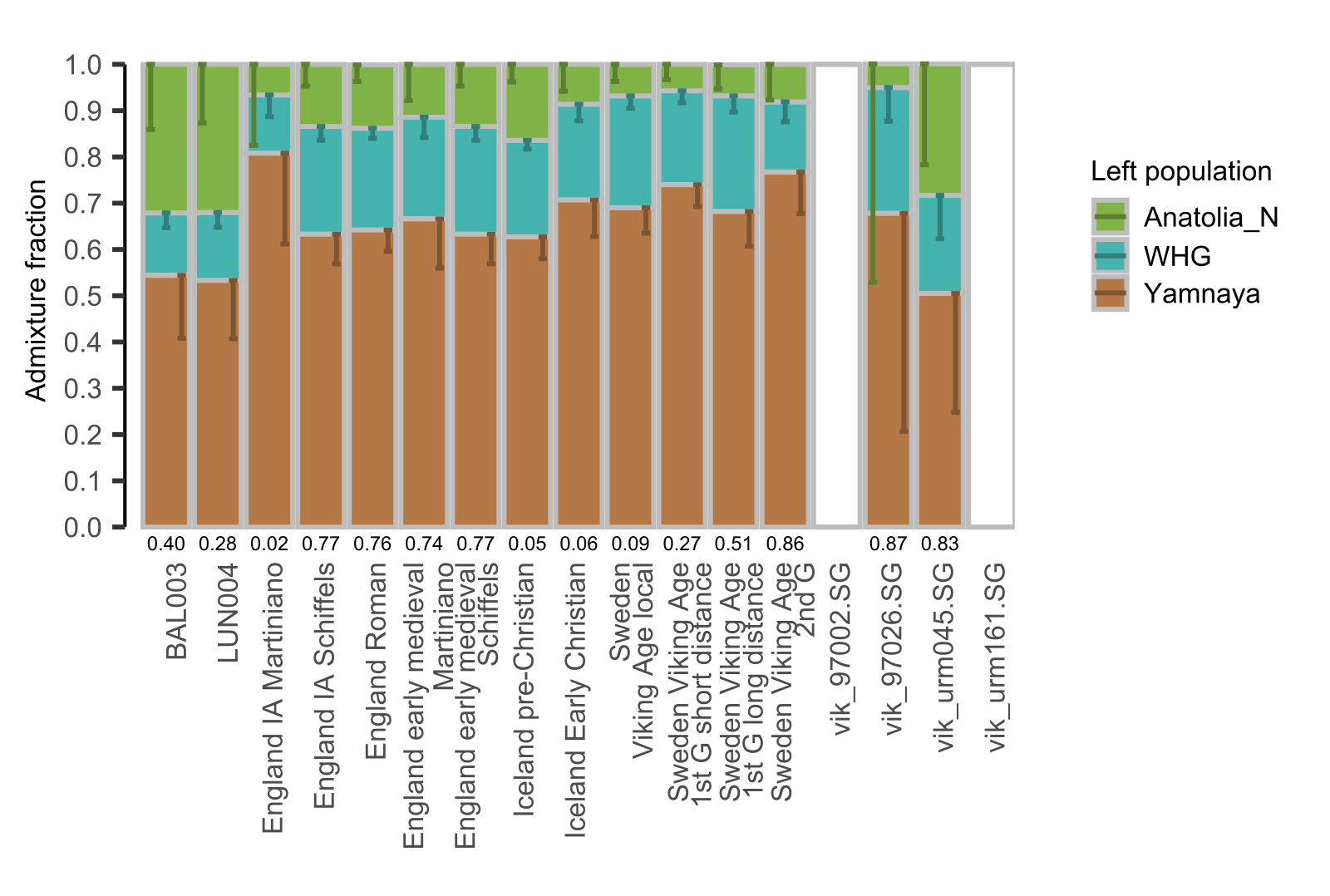

Supplement: S18 Fig — Empty bars are target populations for which the three-way models’ proportion was impossible to estimate using qpAdm. Vik_97002.SG, vik_97026.SG, vik_urm045.SG and vik_urm161.SG are individuals buried in a Viking context in Sigtuna, Sweden but likely migrants of diverse origins. G, generation. (TIF) [file pgen.1010360.s030.tif]

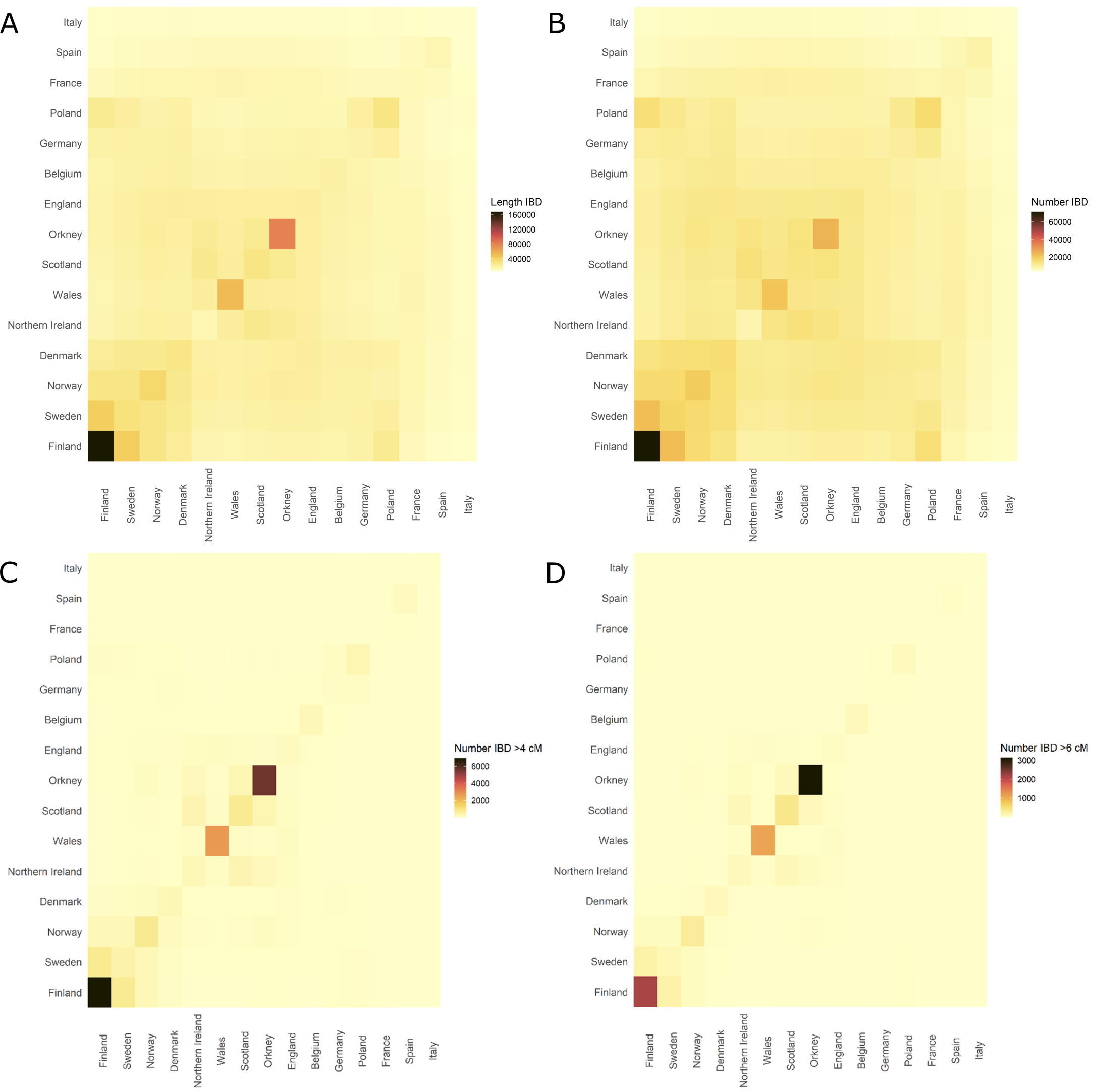

Supplement: S19 Fig — A) Total length of shared IBD segments >1 cM, B) total number of shared IBD segments >1 cM, C) total number of shared IBD segments >4 cM and D) total number of shared IBD segments >6 cM. The number corresponds to the mean of 100 bootstraps drawing 44 random individuals per population. (TIF) [file pgen.1010360.s031.tif]

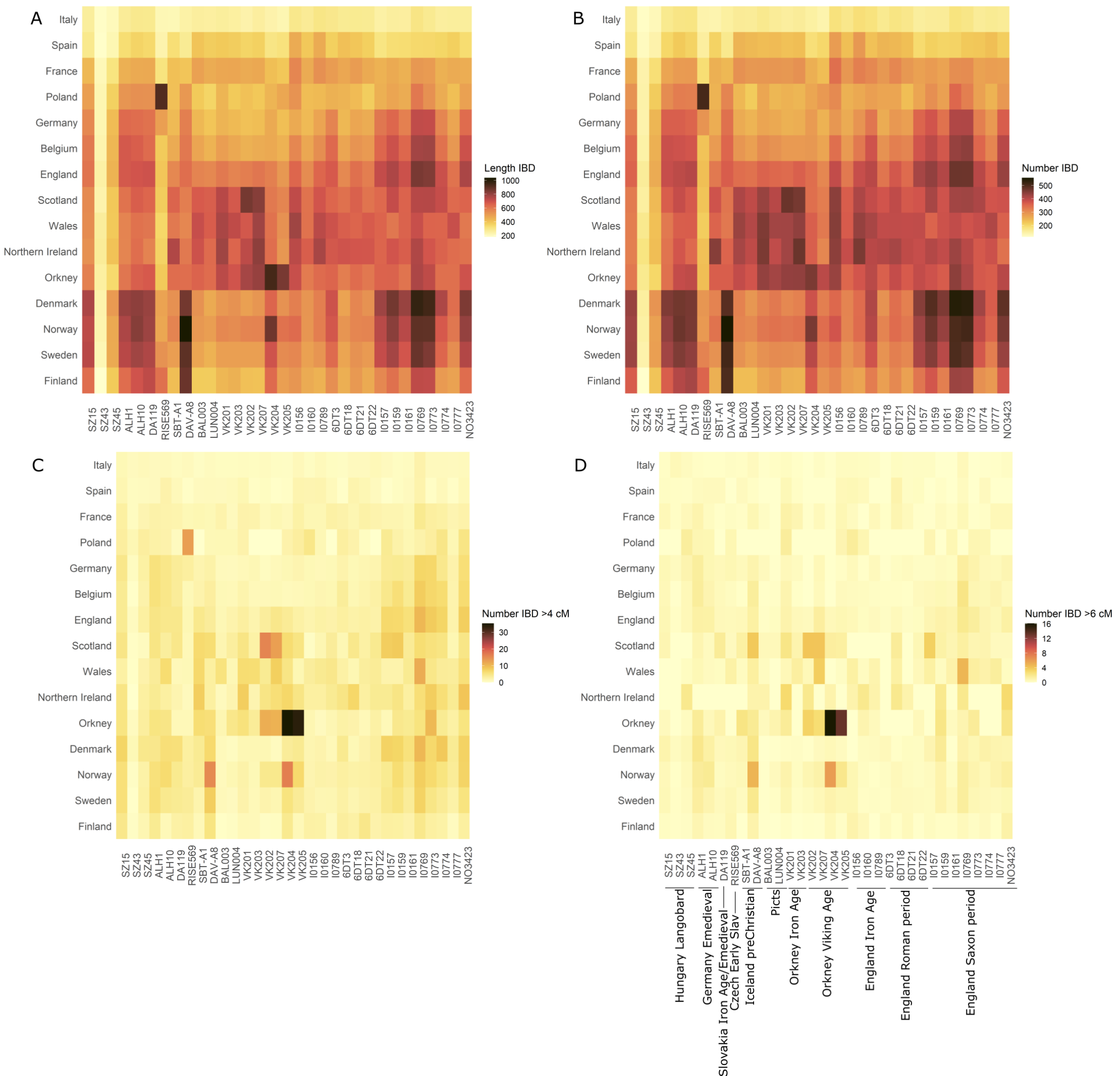

Supplement: S20 Fig — The ancient genomes are the newly imputed genomes and the six ancient Orcadians from Margaryan et al. [9]. A) Total length of shared IBD segments >1 cM, B) total number of shared IBD segments >1 cM, C) total number of shared IBD segments >4 cM and D) total number of shared IBD segments >6 cM. The number corresponds to the mean of 100 bootstraps drawing 44 random individuals per European population. (TIF) [file pgen.1010360.s032.tif]

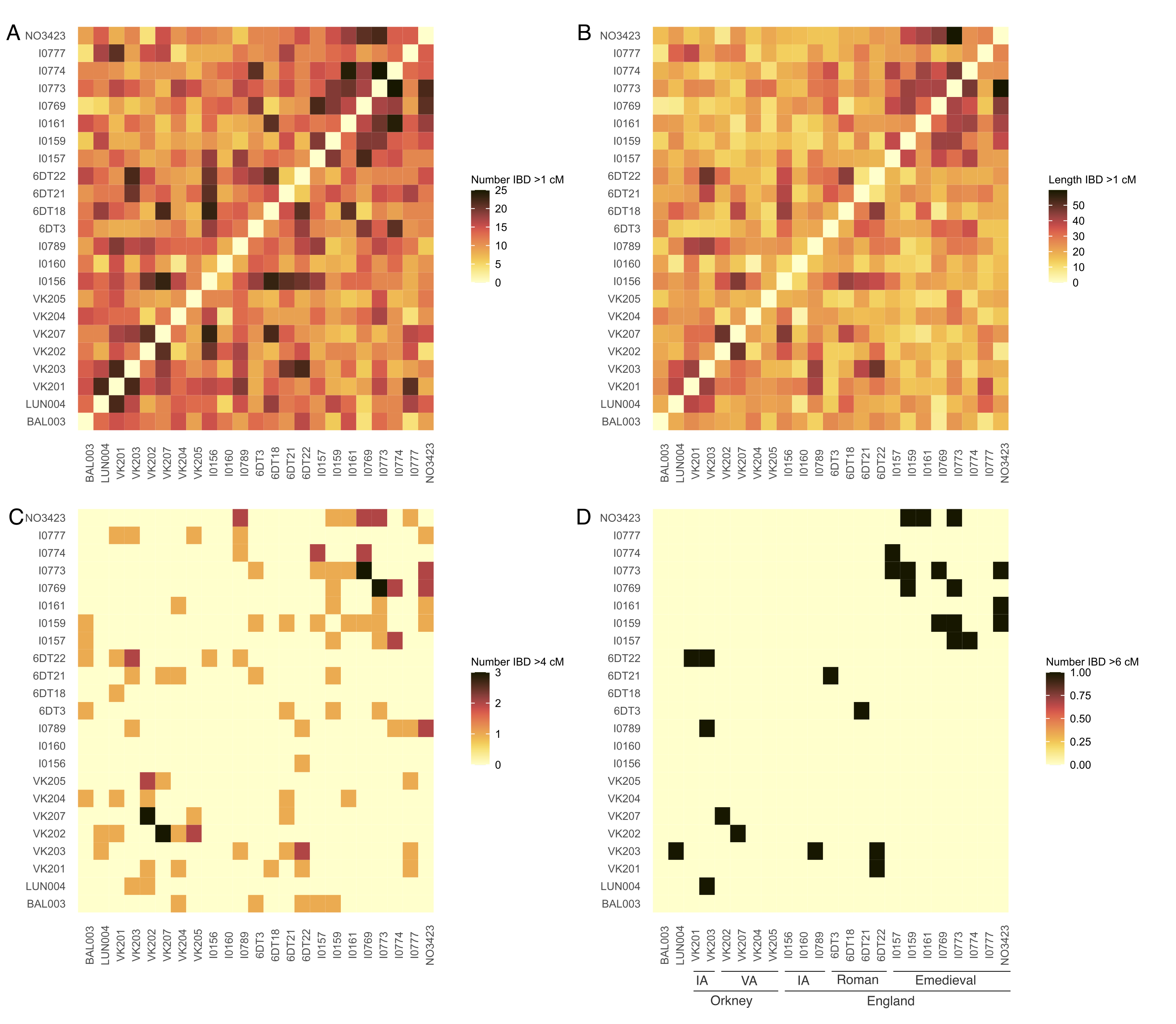

Supplement: S21 Fig — A) Total length of shared IBD segments >1 cM, B) total number of shared IBD segments >1 cM, C) total number of shared IBD segments >4 cM and D) total number of shared IBD segments >6 cM. IA, Iron Age; VA, Viking Age; Emedieval, early medieval. (TIF) [file pgen.1010360.s033.tif]

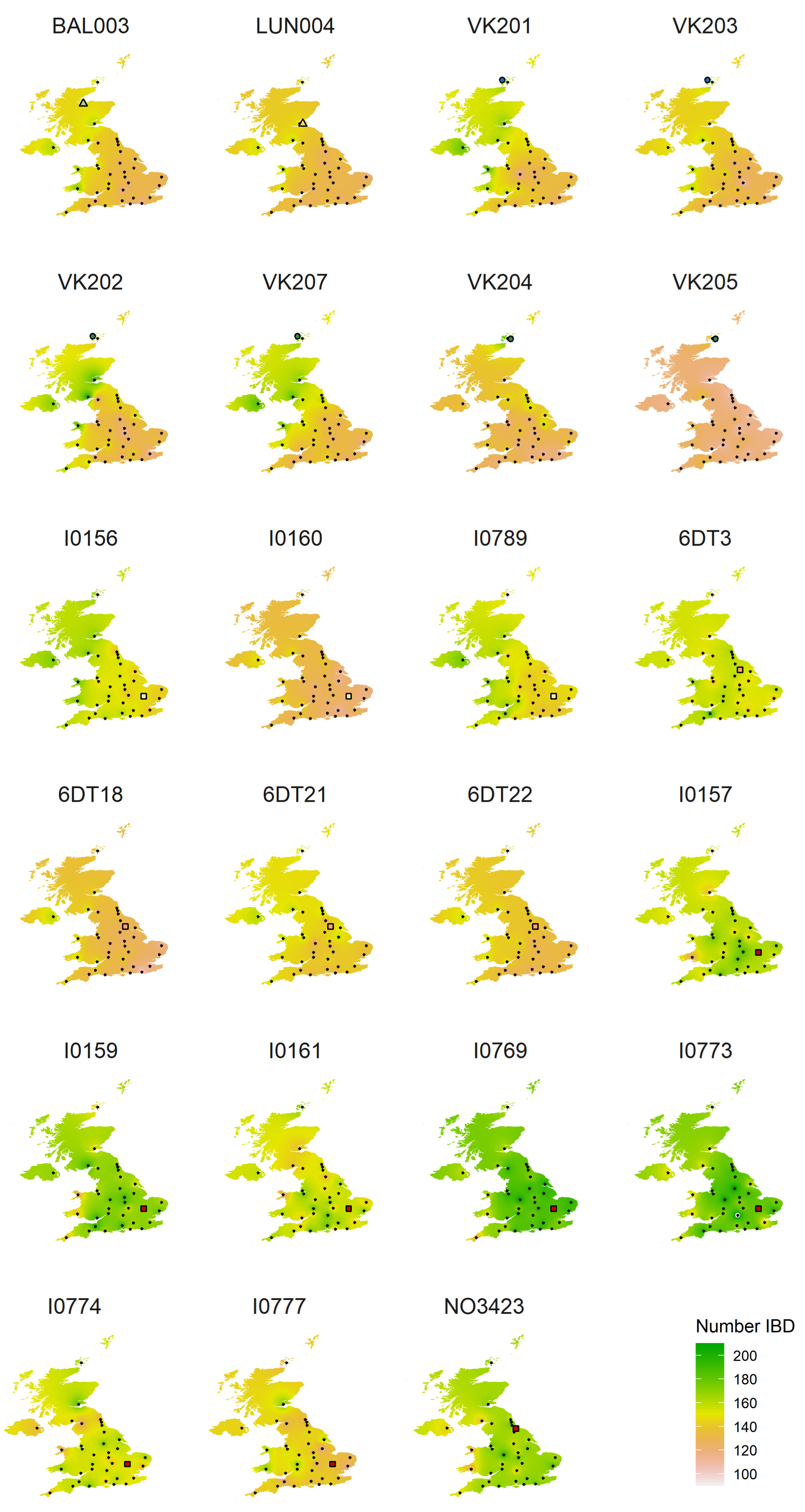

Supplement: S22 Fig — (TIF) [file pgen.1010360.s034.tif]

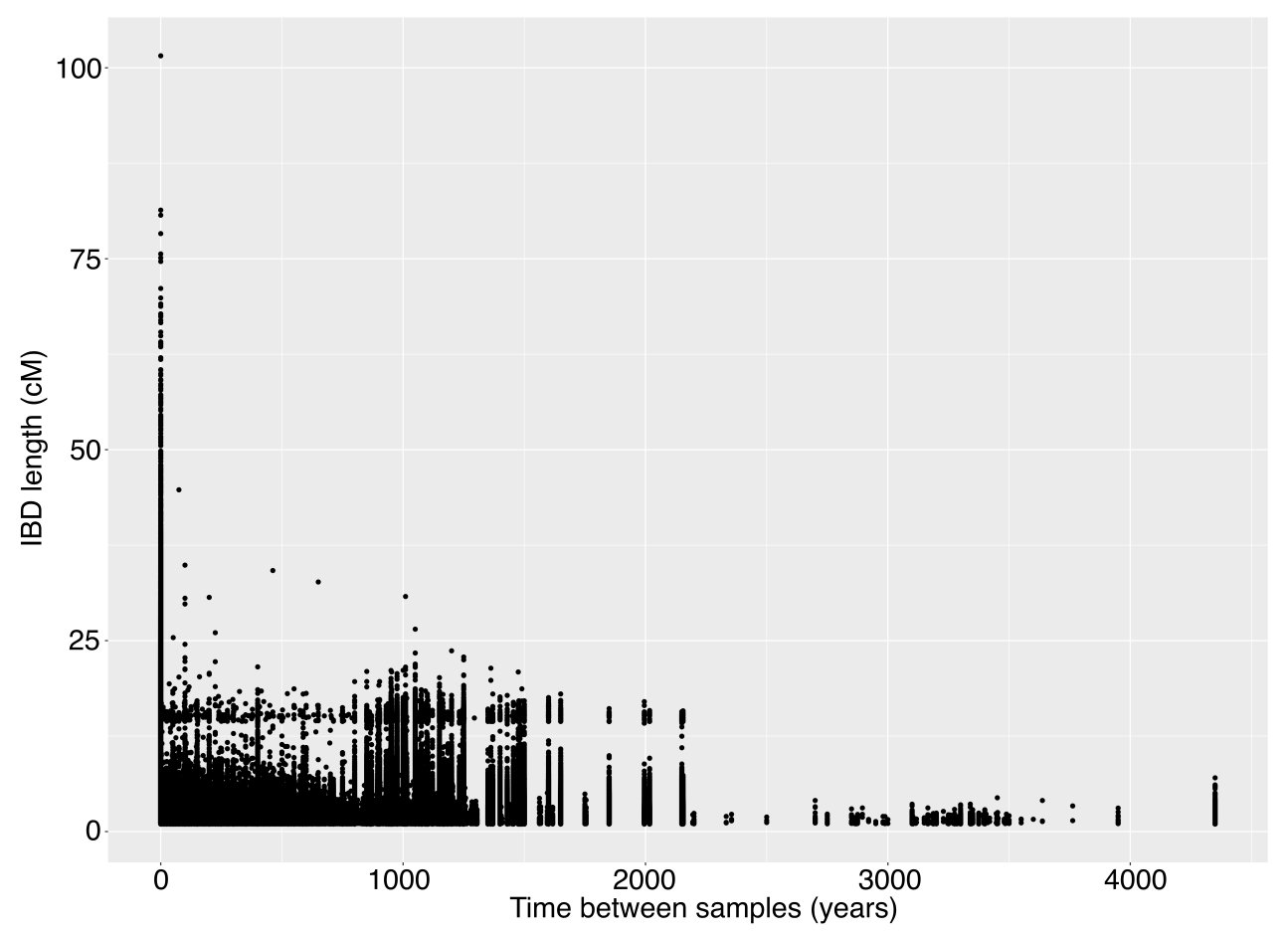

Supplement: S23 Fig — (TIF) [file pgen.1010360.s035.tif]

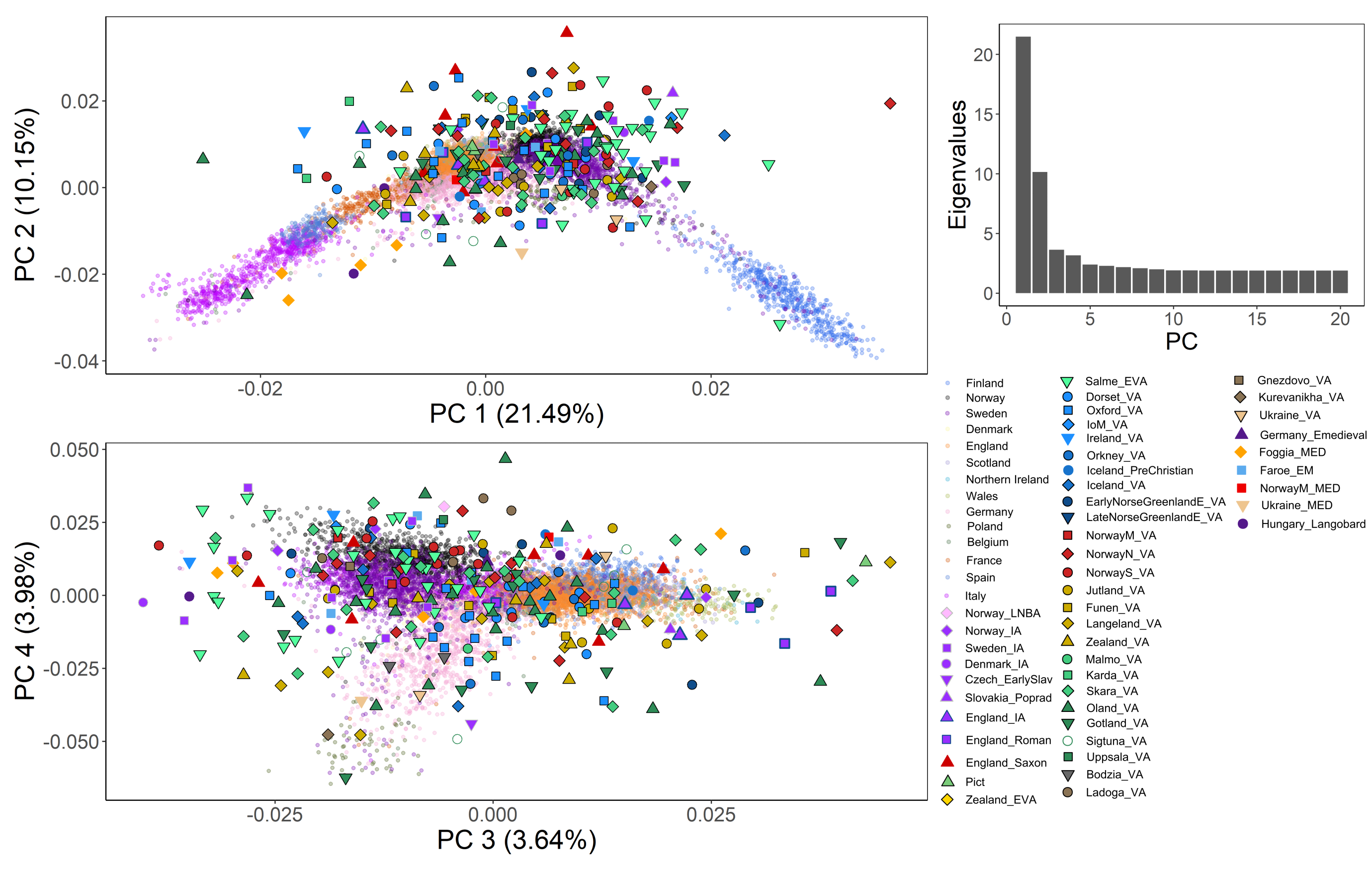

Supplement: S24 Fig — The pseudo-haploid ancient genomes (S7 Table) were projected. This analysis replicates S8 Fig but only using modern genomes for the PCs computation and projected pseudo-haploid ancient genomes. SNPs with maf <5% were removed and pruned (88,369 SNPs remained). (TIFF) [file pgen.1010360.s036.tiff]

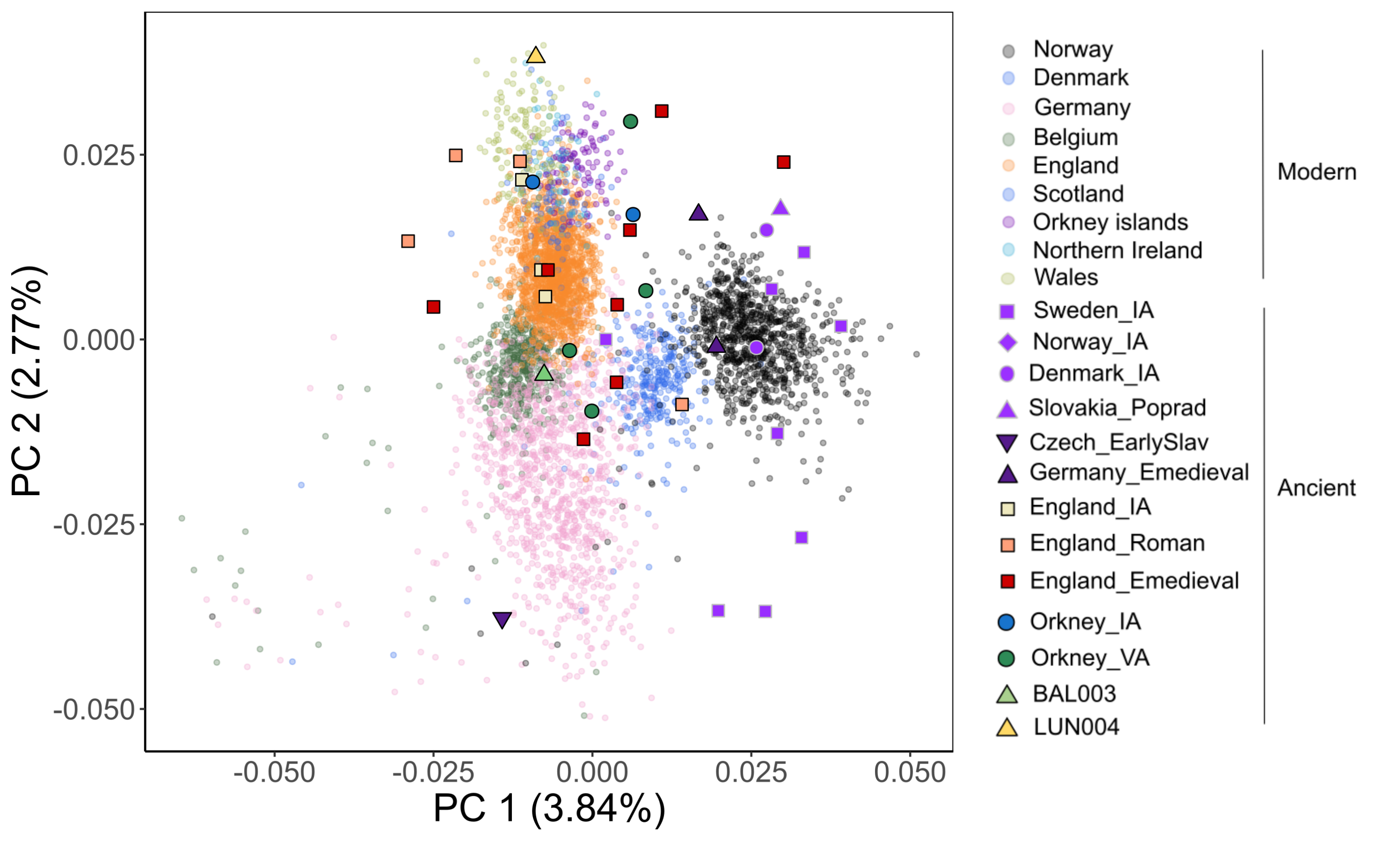

Supplement: S25 Fig — The pseudo-haploid ancient genomes (S7 Table) were projected. This analysis replicates Fig 2A, but only using modern genomes for the PCs computation and projected pseudo-haploid ancient genomes. SNPs with maf <5% were removed and pruned (88,369 SNPs remained). (TIFF) [file pgen.1010360.s037.tiff]

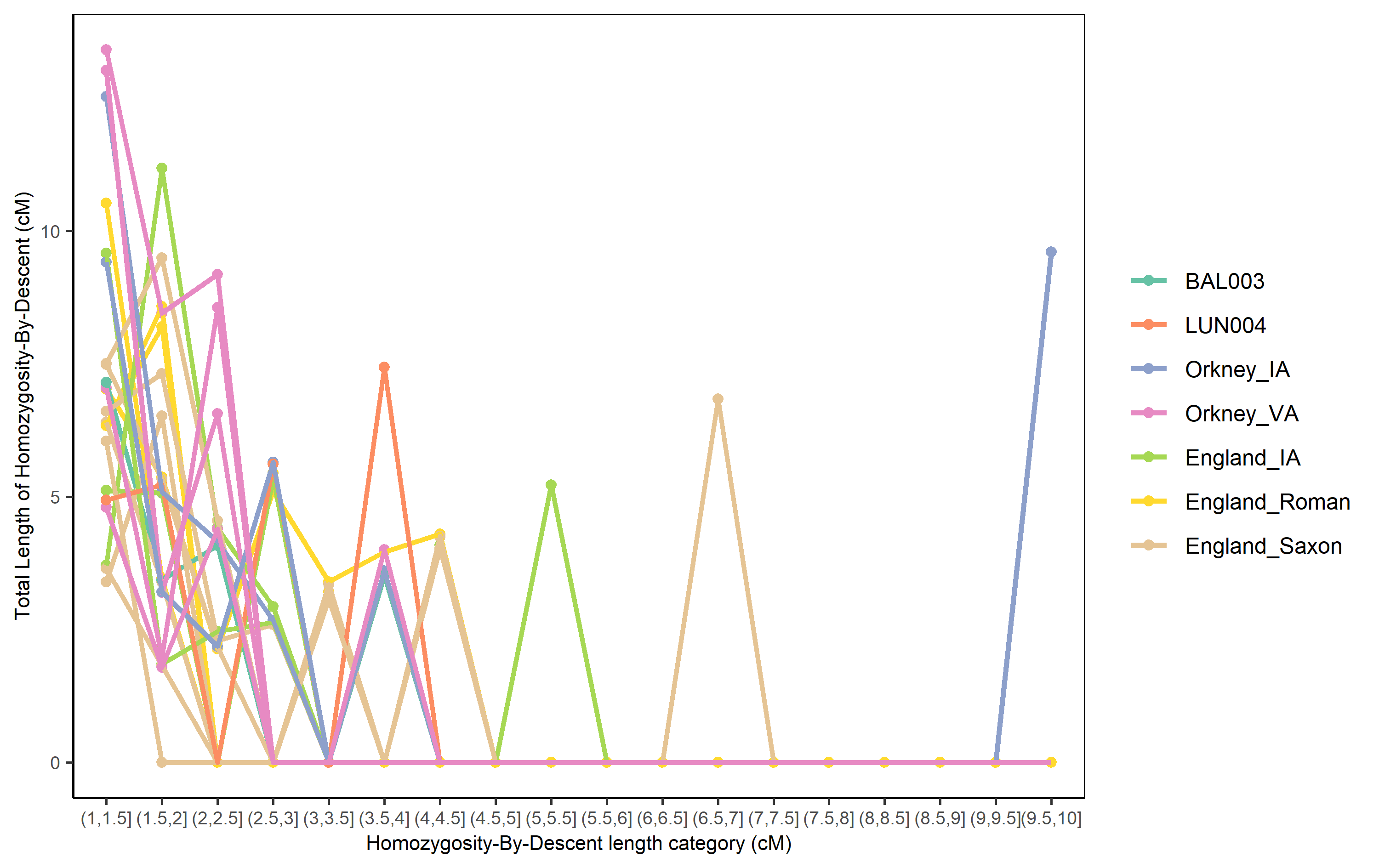

Supplement: S26 Fig — (TIF) [file pgen.1010360.s038.tif]

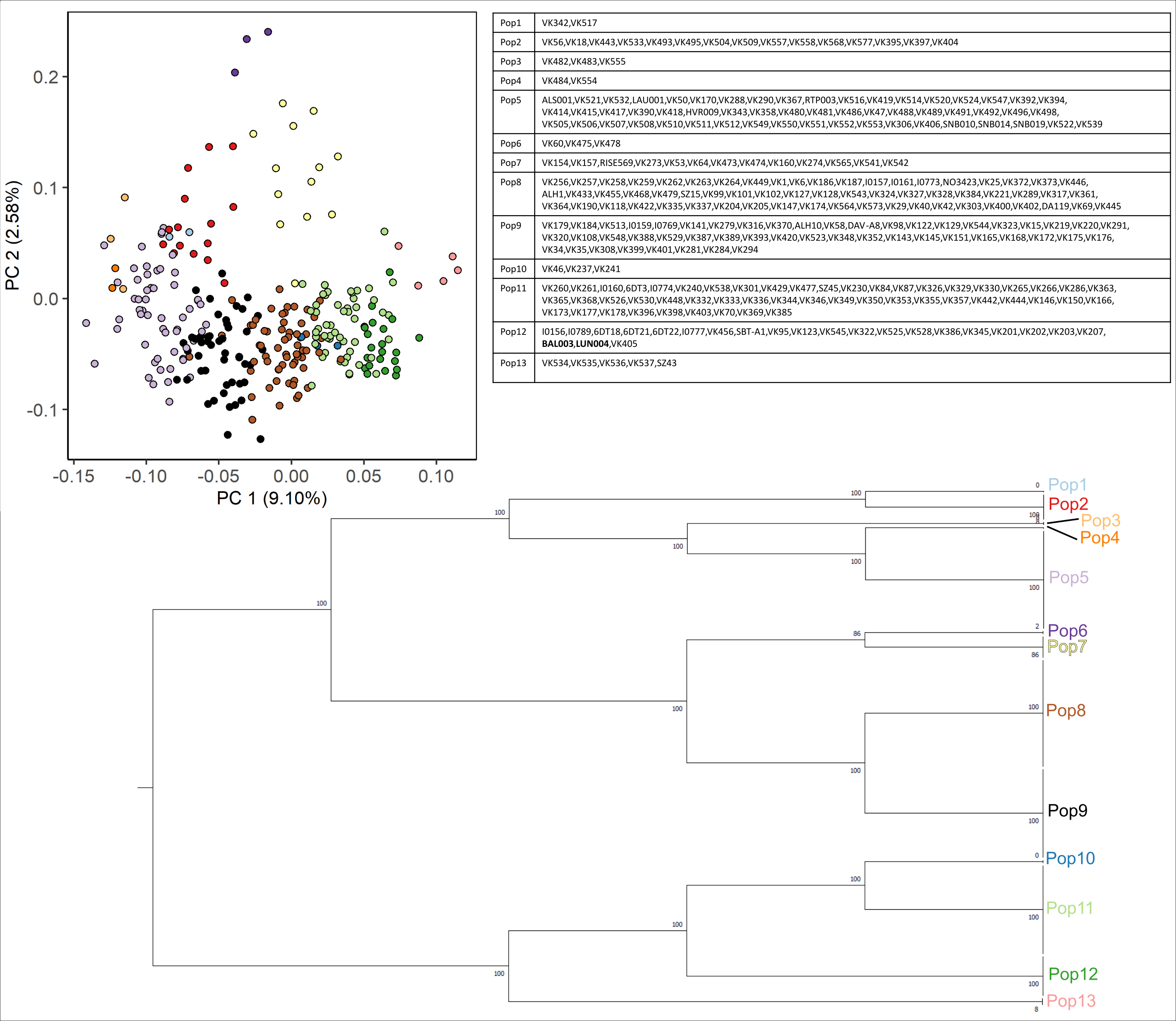

Supplement: S27 Fig — (TIF) [file pgen.1010360.s039.tif]
