# Supplementary material for: Imputed genomes and haplotype-based analyses of the Picts of early medieval Scotland reveal fine-scale relatedness between Iron Age, early medieval and the modern people of the UK
Source: PLoS Genet. 2023 Apr 27;19(4):e1010360. doi: 10.1371/journal.pgen.1010360 (PMC10138790; doi:10.1371/journal.pgen.1010360)
Supplement: S1 Text — (DOCX) [file pgen.1010360.s040.docx]

**S1 Text. Supplementary information.**

**S1.1 The archaeological context of the sites.**

**Balintore**

Balintore today is a village in northern Scotland located in the Highland council region. In 1982, developments near the village (Fearn parish) led to the discovery of two extended inhumations whilst excavating a trench for a new sewer pipe (NRHE 15248) (1). The bodies (accession numbers INVMG1982.082 [listed erroneously as INVMG1985.082 in Sheridan et al. (2)] and INVMG1982.083) were both laid out in long cists in a southwest-northeast position with the head to the southwest. No grave goods were associated with the bodies. Radiocarbon dates suggest the burials date to the 5^th^-7^th^ centuries AD. Other reports of extended inhumations such as NRHE 15259 suggest a larger cemetery, although it should be noted that some possible Pictish burials from Balintore have been determined via radiocarbon dating to be Neolithic in date (3). Balintore is located close to Shandwick where an intricately carved Pictish cross-slab of 8^th^-9^th^ century date suggests some form of high status ecclesiastical or secular focus in the later first millennium AD.

**Lundin Links**

Lundin Links today is a village in Fife in central-eastern Scotland, located on the northern shore of the Firth of Forth. Lundin Links was the subject of rescue excavations in the 1960s following a series of erosion events reported as early as the mid-19^th^ century. The cemetery was located on dunes near the village of Lower Largo, Fife (4). Excavations following storm erosion in 1965 and 1966 revealed a series of cairns covering long cists along with graves without any form of monumental setting. The cairns included circular and rectangular examples and more unusual monuments including the ‘dumb-bell complex’ that consisted of two round cairns joined by an oval setting of stones, and the ‘horned-cairn complex’ comprising several sub-circular cairns and crescentic structures. These two latter monuments are notable for containing multiple inhumations, a relatively unusual aspect of Pictish inhumation practices. Earlier discoveries of human remains from Lundin Links suggest the cemetery was more extensive than that revealed in the 1960s and more human remains have been found in recent decades. The excavations yielded 22 individuals dating to the 5^th^ to 7^th^ centuries AD. Osteoarchaeological analysis suggests some shared familial traits amongst the females interred in the horned cairn complex. Lundin Links is a classic example of a Pictish square and round cairn cemetery as found in eastern and northern Scotland (5,6).

**Radiocarbon dating**

Fourteen radiocarbon dates are available from Balintore and Lundin Links. The dates for ten samples from Lundin Links were already published in Greig et al. (4) (S4 Table). A single additional bone sample from Lundin Links (LUN004) was pre-treated at the Archaeological Chemistry Laboratory, University of Aberdeen. Mandibular fragments associated with the tooth from which genetic data had been maintained were selected and external surfaces of bone fragments were cleaned with the aid of a dental drill with a tungsten carbide burr. Collagen was extracted using the method of Longin (7), with modifications based on the recommendations of Collins and Galley (8), as described in Britton et al. (9). Samples were demineralised in 0.5 M hydrochloric acid at ~5°C for 3-10 days with acid changed at regular intervals. They were then rinsed to neutrality with de-ionized water and gelatinized in a weak acidic (pH 3) HCl solution at 70 °C for 48 h. The liquid fraction containing the gelatinized protein was isolated through filtration using 5-8 μm Ezee™ mesh filters (Elkay Laboratory Products), the remaining solution was then frozen and lyophilised. Yields were calculated (%) to estimate the quantity of preserve collagen, and C:N ratios were determined in order to assess the preservation and integrity of the extract (10). The latter was undertaken at the Scottish Universities Environmental Research Centre (East Kilbride) using a Thermo Scientific^TM^ EA IsoLink^TM^ coupled to a Delta V^TM^ Advantage IRMS via a Conflo IV interface with an analytical error of ±0.3 ‰, a system which enables the co-measurement of carbon, nitrogen and sulphur stable isotope ratios, alongside elemental data allowing the calculation of C:N ratio (11). A sub-sample of extract was then sent to Beta Analytic^TM^ (ISO/IEC 17025:2017) for radiocarbon dating via their commercial services using Accelerator Mass Spectrometry (AMS).

The three tooth samples from Balintore were pre-treated at the Department of Human Evolution, Max Planck Institute for Evolutionary Anthropology (MPI-EVA), Leipzig, Germany, using the method described in Talamo and Richards (12) and Talamo et al. (13). The outer surface of the bones was first cleaned by a shot blaster, and 500 mg of the whole bone was taken. The sample was decalcified in 0.5M HCl at room temperature until no CO_2_ effervescence is observed. 0.1M NaOH was added for 30 min to remove humics. The NaOH step was followed by a final 0.5M HCl step for 15 min. The resulting solid is gelatinized following Longin (7) at pH 3 in a heater block at 75 °C for 20 h. The gelatine was filtered in an Eeze-Filter™ (Elkay Laboratory Products (UK) Ltd.) to remove small (>80 mm) particles. The gelatine was then ultra-filtered (14) with Sartorius ‘VivaspinTurbo’ 30 KDa ultrafilters. Prior to use, the filter was cleaned to remove carbon containing humectants (13,15). The sample was lyophilized for 48 h. The date was corrected for a residual preparation background estimated from pre-treated ^14^C free bone sample. To identify the preservation of the collagen yield, C:N ratio was also estimated. It should be between 2.9 and 3.6, and the collagen yield is not less than 1% of the weight (10,13). Stable isotope analysis was performed in MPI-EVA, Leipzig (Lab Code R-EVA), using a ThermiFinnigan Flash EA with Delta V isotope ratio mass spectrometer. After the evaluation of these values, the samples were sent to Curt Engelhorn Centre for Archaeometry (CEZA) in Mannheim (Germany) (Lab Code MAMS), where they were graphitized and dated (16).

Calibrated date ranges were calculated using the internationally agreed calibration curve of Reimer et al*.* (17) and OxCal v4.4 (18), with conventional radiocarbon ages (19) presented in Table 1, S1 and S4 Tables and the main text. The date ranges were calculated using the maximum intercept method (20) and quoted with the endpoints rounded outward to 10 years, with probabilities calculated using the method of Stuiver and Reimer (1993) (21). The dates were modelled within a simple Bayesian model. At Lundin Links, the modelling estimated that all the dated activity began in *cal AD 391-541* (*95% probability*; S2 Fig, S4 Table) or in *cal AD 425-514* (*68% probability*; S2 Fig, S4 Table) and that dated activity ended in *cal AD 550-666* (*95% probability*; S2 Fig, S4 Table), or in *cal AD 569-625* (*68% probability*; S2 Fig, S4 Table). The estimated span of the dated activity at this cemetery was *0–275 years* (*95% probability*; S2 Fig, S4 Table), or *0–200 years* (*68% probability*; S5 Fig, S4 Table). At Balintore, the modelling estimated that all the dated activity began in *cal AD 114-560* (*95% probability*; S2 Fig, S4 Table) or in *cal AD 414-539* (*68% probability*; S2 Fig, S4 Table) and that dated activity ended in *cal AD 541-906* (*95% probability*; S2 Fig, S4 Table), or in *cal AD 646-553* (*68% probability*; S2 Fig, S4 Table). The estimated span of the dated activity at this cemetery was *0–792 years* (*95% probability*; S2 Fig, S4 Table), or *0–232 years* (*68% probability*; S5 Fig, S4 Table).

**S1.2 Evidence for index misassignment in LUN001 and LUN003.**

LUN001 and LUN003 libraries were single indexed following the protocol from Meyer and Kircher (2010) (22) and multiplexed together with libraries from different samples, including two ancient samples from South America (unpublished) for sequencing (referred to as X1 and X2). Double-indexing protocols (23) were developed after discovering that multiplexed libraries using single indexes were often cross-contaminated with other pooled samples (24,25). Samples misassignment can occur for five known reasons. First, errors in sequencing, library amplification or oligonucleotide synthesis can cause a set of overly similar indexes to become indistinguishable (22). Second, another possible source of sample misidentification is cross-contamination of indexes by the oligonucleotide manufacturer or during later handling. Third, if a multiplex set of libraries is amplified, often following a hybridisation capture step, the PCR can produce chimaeras by recombining different molecules, a process called ‘jumping PCR’ (26,27). Fourth, free-floating primers can be hybridised with the template molecule and amplified in the sequencer during the clustering through the bridge amplification step, a process called ‘index hopping’ (24,28). Fifth, a cluster position can be misassigned by the optical sensor during sequencing, commonly referred to as ‘bleed over’ (28). This last process is usually associated with a bad quality score of the read.

Given the relative position of LUN001 and LUN003 on the PCA (S10 Fig), drifted towards Yamnaya, Russia Afontova and Mal’ta, samples who are closely related to present-day Native Americans (29), and the presence of an ancestry component associated with Native American ancestry in LUN001 and LUN003 (S12 Fig) we suspected that sample misassignment had occurred in the sequencing runs with LUN001, LUN003, X1 and X2. We tested whether sample misassignment could be present in LUN001 and LUN003 libraries. First, we modelled LUN001 and LUN003 with the potential contaminants (X1 + X2) as one source and several ancient Europeans as the second source, Mbuti and modern Native American populations (Surui, Mayan, Piapoco, Karitiana, Pima, Mixe, Quechua, Zapotec and Mixtec) as outgroup using *qpAdm*. LUN001 and LUN003 can be modelled as originating from two sources, an European source and the ancient potential contaminants, with modern Native Americans are set as outgroup (S2 Table, except LUN003 when *Sweden_IA.SG* or LUN004 are set as source 2). This shows that the divergent ancestry in LUN001 and LUN003 is not related to an ancient ancestry shared by all Native Americans and X1 and X2 but an ancestry directly descending from X1 and X2, which is consistent with a significant index hopping occurring between the samples. LUN001 and LUN003 carry 11.4-18.3% and 0.6-5.2% ancestry from X1+X2, respectively. Second, we test whether LUN001 and LUN003 can be modelled as originating from a single ancient European source adding the contaminants X1 and X2 in the list of outgroups. A model including a true source as outgroup should be rejected by *qpAdm*. Models on LUN001 are always rejected (p < 0.05) but not the models on LUN003 (P > 0.05) (S2 Table), which is consistent with a significant sample misassignment within LUN001 but not or less in LUN003.

Contamination estimates using Schmutzi (30) failed to detect excessive contamination in LUN001 and LUN003, because Schmutzi is developed to detect present-day non-deaminated contaminants. But here the potential contamination source is ancient, so is deaminated (S1 Table). LUN001 and LUN003 are females (S4 Fig), while the two ancient samples from South America are males. Thus, we estimated Y-chromosome contamination in LUN001 and LUN003. Although LUN001 has a slightly higher Y-chromosome contamination (4%), LUN003 has Y-chromosome contamination respecting ancient DNA norms (1%) (Table 1), and those values are not high enough to raise any red flags.

An mtDNA-based method was developed to assess sample misassignment rates. We generated a consensus mtDNA sequence for LUN001, LUN003, X1 and X2 using Schmutzi (30) and identified diagnostic mismatch positions. Then, the rate of X reads in LUN001 or LUN003 was estimated as $r= \frac{\sum_{i=1}^{\mu} a_{i}}{\sum_{i=1}^{\mu} b_{i}}$. Here, µ is the mismatch sites, i.e., where the consensus allele between the ancient South Americans and LUN001 or LUN003 differ, a is the number of reads carrying the ancient South American alleles at each mismatch position, and b is the total number of reads at each mismatch position. The method was used considering transversion sites only (3 sites using X1 and 8 sites using X2, covered by 100-900 reads). The mtDNA reads misassignment rate was estimated per sequencing lane. The sequencing lane L004_screen was completed before pooling and deep sequencing with the ancient South Americans, so we do not expect any sample misassignment in this lane.

The sample misassignment rates estimated using mtDNA reads on the transversions are low (S3 Table). The sample misassignment rate is always null in the sequencing lane made before pooling (L004_screen). Although many sequencing lanes show greater misassignment rates than the L004_screen, the misassignment rate is low showing that the mtDNA reads are not substantially affected by this issue. This result could mean that either low cross-contamination had a considerable impact on the later analyses, or the autosomal reads were more impacted by sample misassignment than the mtDNA reads. For instance, mtDNA-based contamination estimate depends on the tissue and are not necessarily representative of the nuclear DNA contamination since there are several orders of magnitude difference in the mtDNA-to-nuclear DNA ratio per cells (31).

To be conservative, we decided not to interpret the autosomal data from LUN001 and LUN003, but we are confident that the mtDNA haplogroup assignments are robust and can include LUN001 and LUN003.

**S1.3 Genomic analysis on pseudo-haploid sequences.**

**Genotyping of BAL003, LUN004, LUN003 and LUN001 and comparison to SNP reference panels representing ancient and modern human genetic diversity**

First, the few first and last bases of the molecules from USER-treated double-stranded BAL003 and LUN004 libraries still carry uracils that can influence variant calling (S3 Fig). So, the five first and last bases were ‘soft-clipped’, meaning that we reduced the base quality of any ‘T’ in the first five bases and any ‘A’ in the five last bases to a PHRED score of 2 (32). We applied a pseudo-haploid sequence calling to each of the sample sequenced to a sufficient coverage (BAL003, LUN004, LUN003 and LUN001) (one allele on each polymorphic site is randomly called when at least two reads overlap the site). For pseudo-haploid sequence calling, we used mpileup from SAMTOOLS v1.9 (33), with options computation of base alignment quality (BAQ) computation disabled (-B), read group tag ignored (-R) and base quality <30 removed (-q 30) and pileupCaller from SequenceTools v1.2.2. To reduce the potential for deamination resulting in an excess of C-to-T and G-to-A substitutions, we limited the base calling to transversion substitutions.

The newly-generated genomes were merged with ancient and modern individuals from the Allen Ancient DNA Resource ([https://reich.hms.harvard.edu/](https://reich.hms.harvard.edu/downloadable-genotypes-present-day-and-ancient-dna-data-compiled-published-papers), S7 Table). The first dataset, ‘HO’, holds individuals merged over the 597,573 polymorphic sites in the Human Origin array (34). The second dataset ’1240K’ contains individuals merged over the 1,233,013 polymorphic sites discovered in Mallick et al. (2016) (35), and recovered through in-solution target capture. In the ‘HO’ dataset, we kept 3,037 present-day individuals including two Saami sequenced for the Simon Genome Diversity Project. In the ‘1240K’ dataset, we kept 243 present-day individuals (35). In both datasets, we kept previously published ancient genomes from the Palaeolithic to the medieval period, 580 individuals were generated via shotgun sequencing ('shotgun') and 1,052 via in-solution target capture ('capture') (3,29,32,36–76). Any biological 1^st^- and 2^nd^-degree relatives and individuals showing evidence of contamination were discarded. The ancient genomes are all pseudo-haploid, and transitions were removed before the analysis. Finally, we merged the comparative datasets with the newly generated early medieval samples from Pictland and converted the EIGENSTRAT format to the plink binary format using the MERGEIT and CONVERTF programs from EIGENSOFT package v6.1.4 (77).

**Principal Component Analysis**

We computed a principal component analysis (PCA) on the present-day western Eurasian and worldwide populations from the ‘HO’ dataset using all substitutions via SMARTPCA (34). For the first analysis, the program kept 1,056 present-day western Eurasian individuals after outlier removal and used 524,081 SNPs. For the second analysis, the program retained 2,803 worldwide individuals and used 564,169 SNPs. We projected the ancient individuals with the option lsqproject: YES (111,208 SNPs).

BAL003,LUN004, LUN003 and LUN001 pseudo-haploid genomes are projected within the European diversity in a principal component analysis constructed from present-day Eurasians (S10 Fig). BAL003 and LUN004 fit within the north-western European cluster, including present-day individuals from Britain, France, Norway and Iceland (S10 Fig). They cluster within the range of previously published ancient genomes from the British Isles dating from the Bronze Age to the medieval period (56,64,69). LUN003 and LUN001 deviate from this cluster and fit clusters made of eastern and north-eastern present-day Europeans, respectively. They still fit the cluster made from Bronze Age individuals from the British and Irish Isles, but they do not fall within the post-Bronze Age individuals group, seemingly because of a relative greater affinity to either the Yamnaya-related individuals or the Afontova Gora and Mal’ta genomes.

**ADMIXTURE**

To estimate the ancestry components in the samples for comparison to modern world populations and published ancient data, we used a model-based clustering approach from the program ADMIXTURE v1.2 (78). All ‘HO’ modern individuals except the two Saami were used, since the different sequencing methods might bias the component calculation and ancestry attribution. We restrained the ancient dataset to genomes generated via shotgun sequencing to prevent reference bias. To avoid incorrect genetic component assignation induced by biases between diploid and pseudo-haploid data, we transformed all sequences into pseudo-haploid sequences, including the modern individuals. The remaining 111,208 positions were subsequently pruned for SNPs in strong linkage disequilibrium using PLINK v1.9 (79), with the parameter –indep-pairwise 200 25 0.4 to yield a final set of 85,655 SNPs. ADMIXTURE was run with cross-validation (CV) enabled using –cv lag for all ancestral population numbers from K=2 to K=20.

The genetic clustering of ancient and modern worldwide populations using ADMIXTURE is optimal for K = 15 (S6 Fig), at which we observe the three main ancestry components constituting present-day Europeans in BAL003, LUN004, LUN003 and LUN001 (S12 Fig). The three clusters stem from 1) the Western and Scandinavian Hunter-Gatherers (WHG and SHG) (light blue), 2) Early European Farmers (EEF), who derived the majority of their ancestry from Middle Eastern Early Farmers (light green) and 3) Caucasus Hunter-Gatherers (CHG) (orange). The CHG cluster emerged in Europe via migration during the Late Neolithic and Bronze Age from Yamnaya-like steppe pastoralists (S12 Fig) (36,80). BAL003 and LUN004 share similar admixture proportions with European samples dating to the Bronze Age, Iron Age, and medieval periods (S12 Fig). In addition, LUN004 displays a component maximised in present-day South Asians (red), which is also observed in central Asians and Europeans until the Iron Age (including the Iron Age and Roman samples from England) (S13-S15 Figs). However, affinity with central Asians is not supported by *D-statistics* nor *qpAdm* ancestry modelling (S16 and S18 Figs). LUN001, and to some extent LUN003, carry a genetic component maximised in present-day Native Americans (S12 and S13 Figs), which explains the deviation observed in the PCA (S10 Fig), since the population leading to the Mal’ta genome admixed with the ancestors of the first people settling in the Americas (29). This signal is most likely not genuine but rather an artefact from sample misassignment occurring with two co-sequenced ancient genomes from South America (S1.2 Text).

**Genetic relatedness based on *f-statistics***

*Outgroup-f3*

Individual relatedness to present-day Eurasian populations was performed using *outgroup-f3* statistics in the admixr R package (34,81). *F3-statistics* f3(X; A, B), with X, A and B being individuals or populations, measure allele frequency correlation between groups to infer relatedness and reveal admixture. When X is an equidistant outgroup to A and B, *outgroup-f3* becomes a genetic drift measure between A and B. To measure relatedness between individuals from Pictland and present-day Eurasians, we computed *f3(Mbuti; modern Eurasian population, BAL003/LUN004)* using the ‘HO’ dataset.

Using outgroup *f3-statistics* BAL003 and LUN004 are more related to western and northern Europeans (S8 Table). However, BAL003 shares more genetic drift with Norwegians and LUN004 with Scots (S7 Table). Yet, the standard errors overlap with several European populations (S8 Table), suggesting great uncertainty in using this method to resolve population affinity.

*D-statistics*

To obtain information on individual or population admixture, we performed outgroup *D-statistics* of the form (A, B; C, Mbuti) using the D function from the admixr R package (34,81). A, B and C are either present-day or ancient populations/individuals. The outgroup Mbuti is expected to be equidistant to all tested samples. A result equal to 0 means that the proposed tree (((A, B), C), Mbuti) is consistent with the data. If D deviates from 0, there are more alleles shared than expected given the proposed tree either between A and C (D > 0) or between B and C (D < 0).

*D-statistics* were generated for different configurations of populations using transversions only from the '1240K’ dataset when the tests involved only ancient populations (not considering the modern Mbuti outgroup) or using the ‘HO’ dataset when the tests involved modern populations. We restricted the *D-statistics* to tests involving either only shotgun data for population A and B as *D(shotgun, shotgun; C, Mbuti)* or only capture data for population A and B as *D(shotgun, shotgun; C, Mbuti)* or *D(capture, capture; C, Mbuti)* to avoid wet-lab induced and reference biases between shotgun sequencing and capture to impact the *D-statistics*. Only tests supported with >10,000 SNPs were reported.

We studied continuity with older or contemporaneous European populations. D-statistics reveal that BAL003 and LUN004 form a clade with the samples from England dated to the Iron Age and Roman period to the exclusion of any other modern and ancient test population (S16 Fig, S9 Table). Here, we consider excess allele sharing to be statistically significant when both *Z-score(BAL003/LUN004, England_IA/Roman; X, Mbuti) > 2* to account for stochasticity in low coverage samples. However, we could not find a closer affinity between BAL003, LUN004 and any of the contemporaneous populations present in England (Iron Age, Roman or early medieval people) using *D-statistics* as *D(ancient England 1, ancient England 2; BAL003/LUN004, Mbuti)* = 0, -2 < Z-score < 2. We also tested for gene flow with the hypothetical ancestor of BAL003 and LUN004 following the split between Iron Age and Roman samples from England and Iron Age and medieval continental Europeans. The test *D(England_IA/Roman, ancient IA/medieval European; BAL003/LUN004, Mbuti)* = 0, -2 < Z-score < 2 (S10 Table) supports that BAL003 and LUN004 were symmetrically related to any Iron Age or medieval Europeans (when of European ancestry) (37,38,43,45,50,51,68,76), and the Iron Age or Roman from England (56,69).

**Testing for admixture model using *qpAdm***

We estimated ancestry proportions modelling a target population as a mixture of a set of left (source) populations differentially related to a set of right (outgroup) populations using *qpAdm* (80). Briefly, *qpAdm* creates a “full” model of perfect virtual fit to the data (one free parameter is assigned to each *f4-statistic*). It compares the “full” model to *f4-statistics* generated based on each tested model. A small p-value (<< 0.05) means the model fits the data worse than a “full” model. This program is based on a matrix of *f4-statistics* of the form F_ij_ = f4(L_1_, Lj; R_1_, R_j_). L1 is the target population, L_j_ a potential source population and R is an outgroup population. They are susceptible to SNP ascertainment bias, ancient DNA damage bias, and differences between shotgun and capture data (82). This analysis was conducted on the ‘1240K’ dataset restricted to ancient shotgun data.

We tested whether BAL003 and LUN004 were consistent with descending from a single gene pool from Iron Age or early medieval European populations. In practice, we tested whether one of the following left populations could explain BAL003 and LUN004 observed diversity: *England_IA.SG*, *England_Roman.SG*, *England_Emedieval.SG*, *Czech_HallstattBylany.SG*, *Estonia_IA.SG*, *Estonia_Medieval.SG*, *Germany_Early_Medieval_elongskull.SG*, *Germany_Early_Medieval_normskull.SG*, *Hungary_Scythian.SG*, *Sweden_IA.SG*, Sweden_*VikingAge.local.SG*

In this part, *England_IA.SG* and *England_Emedieval.SG* are the damage repaired samples from (69). We used the following outgroup set: *Iran_TepeAbdulHosein_N.SG, Russia_Kostenki14.SG, Switzerland_Bichon.SG, Russia_Sunghir.SG, Russia_Shamanka_Eneolithic.SG, Alaska_LP.SG, DevilsCave_N.SG, Yana_UP.SG, Estonia_BA.SG, Ireland_BA.SG*

BAL003 and LUN004 genetic diversity are consistent with originating from a single source from several Iron Age or early medieval period European populations, including the ancient samples from England dated from the Iron Age, Roman and early medieval periods (S17 Fig). As a result, we could not disentangle the fine-scale affinity between Iron Age and Roman from England and other above-mentioned Iron Age and medieval Europeans (36,37,48,50,56,69).

Then, we inferred mixture proportions with the three primary sources constituting current European diversity, as represented by the following sampled populations: WHG, Anatolia Neolithic (the main source for EEF ancestry) and the steppe Yamnaya. BAL003, LUN004, Iron Age, Roman and early medieval from England, ancient Icelandic, and the Scandinavians from the Viking Age were consecutively used as a target for comparison. As the ‘Sweden_VikingAge.outliers.SG’ group includes diverse individuals based on their genetic ancestry and isotopes studies (50), we modelled these four individuals buried in Sweden separately. The following outgroup set was used: *Iran_TepeAbdulHosein_N.SG, Russia_Kostenki14.SG, Switzerland_Bichon.SG, Russia_Sunghir.SG, Russia_Shamanka_Eneolithic.SG, Alaska_LP.SG, DevilsCave_N.SG, Yana_UP.SG.*

Ancestry modelling using *qpAdm* confirm that LUN004 and BAL003 were best modelled as a mix of Western European HG (WHG.SG), Anatolian farmers (Anatolia_N.SG) and Steppe pastoralists (Russia_EBA_Yamnaya_Kalmykia.SG, see S7 Table for population labels); these relationships are expected in present-day western and central Europeans (S18 Fig) (32,80). Likewise, the Viking Age Scandinavians (50), Iron Age, Roman and early medieval genomes from England (56,69), and the ancient Icelandic population (83) fit the same European three-way admixture model (S18 Fig).

During the Bronze Age, populations from England and Wales became differentiated from populations of Scotland, as they were admixed with migrants from continental Europe carrying an excess of EEF ancestry and this pattern was still observable during the early Iron Age (84). We observed an increase of Anatolian Neolithic ancestry in the Iron Age individual buried in southern England (13.4% ± 4.7 SE), compared to an Iron Age individual buried in northern England (6.6% ± 17.5 SE) (56), replicating Patterson et al. (84) results (S18 Fig). However, the observed difference is not significant. We noticed that the individuals from a Roman cemetery in northern England (13.7% ± 3.6 SE) (56) and individuals dated to the early medieval period, both from southern England, (13.4% ± 4.7 SE) (69) exhibit Anatolian Neolithic ancestry proportions close to those of the Iron Age southern England individuals. Individuals dating to the early medieval period in northern England show an intermediate Anatolian Neolithic ancestry proportion (11.4% ± 7.8 SE) (56). Thus, we detected an increase in Anatolian Neolithic ancestry in BAL003 and LUN004 compared to Iron Age and Roman groups from England (S18 Fig), but the difference in our estimates is not statistically significant. We could not detect any excess sharing of derived alleles between England or Scotland Iron Age samples and England or Scotland from the Middle and Late Bronze Age, tested as *D(England_MBA/LBA, Scotland_MBA/LBA; LUN004/BAL003/England_IA.SG/England_Roman.SG, Mbuti)* = 0, -2 < Z-score < 2. An excess of EEF-related ancestry in the two individuals from Scotland compared with contemporaneous groups from England could suggest admixture with EEF-rich populations from Britain or Europe (84). However, the relatively small sample size compared to the one in Patterson et al. (84) limits the power of these tests.

**S1.4 Imputation of the early medieval genomes from Pictland and published ancient genomes.**

**Imputation method**

Reads of the published ancient genomes >35 bp, unmapped or with a mapping quality (MAPQ) >30 were retained using SAMTOOLS v1.7 (33). PCR duplicates of the published ancient genomes were removed using PICARD v2.0.1 MarkDuplicates (85), except for samples from Martiniano et al. (2016) (56) published with duplicates previously removed using rmdup from SAMTOOLS v1.7 (33). In brief, we followed the imputation pipeline published in Margaryan et al. (87), using a similar approach as in (45,49,86,88) with the only exception that we used the most recent GLIMPSE software (89) based on the Li and Stephen model (90) as the previously extensively used BEAGLE software (91,92), but that has shown better imputation accuracy and efficiency (89).

The read groups were replaced using PICARD v2.0.1 (85). The reads were realigned using GATK v3.8.1 (93) using Mills and 1000G gold standard indels (<https://gatk.broadinstitute.org/hc/en-us/articles/360035890811-Resource-bundle>). The MD tag of the newly realigned reads were updated and had the extended BAQs calculated using SAMTOOLS v1.7 calmd -E (33). The few first and last bases of the molecules from USER-treated double-stranded libraries still carry uracils that can influence variant calling (S3 Fig). So, the five first and last bases were ‘soft-clipped’, meaning that we reduced the base quality of any ‘T’ in the first five bases and any ‘A’ in the five last bases to a PHRED score of 2 (32).

Genotypes from the genomic positions in the 1000G phase 3 (94), restricted to 77,818,191 biallelic SNPs, were called. The USER/UDG enzymes cannot remove deamination at methylated sites (Cs are substituted directly into Ts), so variants in CpG context were excluded for full and half-USER/UDG treated samples. The SNPs in CpG context were defined as one of the reference or alternative alleles being a C followed by a G (3,462,945 variants removed).

SNPs were called following the workflow suggested for imputation with GLIMPSE (<https://odelaneau.github.io/GLIMPSE/tutorial_b38.html#run_likelihoods>). First, Genotype likelihoods were generated using BCFTOOLS 1.9 mpileup (95), allowing for BAQ recalculation (-E) and skipping indels (-I). We then called the genotypes using BCTOOLS 1.9 call with the multiallelic caller algorithm (-m) (96) and -Ai and -C *alleles* options. For non-damaged repaired genomes, genotypes potentially resulting from deamination were set as missing, replacing 0/1 and 1/1 genotypes by ‘./.’ where the reference allele is C or G, and the alternate is T or A or replacing 0/0 and 0/1 genotypes by ‘./.’ where the reference allele is T or A and the alternate is C or G.

The genomes were merged using BCFTOOLS 1.9. Missing genotypes were imputed on each genome separately by providing the 1000G phase 3 dataset (<http://bochet.gcc.biostat.washington.edu/beagle/1000_Genomes_phase3_v5a>) split in chunks of 15,000 markers overlapped by 3,000 markers and the GRCh37 genomic maps provided by GLIMPSE (89). The missing genotypes were imputed with five burn-in iterations and five main iterations using GLIMPSE (89). After ligating the chunks and merging the chromosomes, the imputed genotypes with a genotype probability ≥0.99 were kept using BCFTOOL 1.9 filter (95).

**Imputation allele frequency bias**

On the Principal Component Analysis (PCA) calculated using the newly imputed genomes in this study, the data from Margaryan et al. (87) and the EU and UK dataset, we noticed a pipeline/imputation bias on PC3, mainly differentiating the ancient genomes imputed by (87) and the newly imputed genomes in this study (S8 Fig). This bias could be caused by different algorithms between the two software programs used. More specifically, (87) imputed the ancient genomes using BEAGLE v4.1 (91). The algorithm used both the information from a set of reference haplotypes and the haplotypes found in the target genomes whereas GLIMPSE (89) uses only the set of modern reference haplotypes. Thus, any errors in the low coverage of the ancient genomes from (87) in turn impacted the whole set of imputed ancient genomes.

We hypothesised that genome coverage influenced the imputation quality, together with the algorithms and the set of target genomes. Therefore, we tested for correlation between coverage and coordinates on the first three PCs in the genomes imputed from Margaryan et al. (87) and in the newly imputed genomes. We tested the correlation between coverage and coordinates on PCs using Kendall’s Tau-b correlation from R v4.0.2 cor.test. The coverage is positively correlated with the coordinates on PC3 within the data imputed in Margaryan et al. (87) (Kendall’s correlation test, n = 252, τ = 0.25, p = 3.76x10^-9^) and within the newly imputed genomes in this study (Kendall’s correlation test, n = 33, τ = 0.26, p = 0.03). The ancient genomes with high coverage are closer to the present-day individuals on PC3 (S9 Fig). However, the coordinates on PC1 and PC2 are not significantly correlated with the coverage nor within the data imputed in Margaryan et al. (87) (Kendall’s correlation test, n = 252, τ_PC1_ = -0.05, p_PC1_ = 0.24; τ_PC2_ = -0.01, p_PC2_ = 0.76) nor within the newly imputed genomes in this study (Kendall’s correlation test, n = 33, τ_PC1_ = -0.06, p_PC1_ = 0.61; τ_PC2_ = -0.13, p_PC2_ = 0.29), suggesting we could rely on the results from PC1 and PC2.

We also compare genome affinities based on PCA using the more traditionally used approach projecting pseudo-haploid ancient genomes on axes computed from modern genomes (S24 and S25 Figs). It is noticeable that the imputation bias on the PC3 axis from S8 Fig disappear when projecting ancient pseudo-haploid genomes (S24 Fig). When focusing on western European diversity, the relative position of the ancient genomes are similar using either the ancient imputed genomes in the PCs computation (Fig 2A) or with projecting the ancient pseudo-haploid genomes (S25 Fig), and replicates previous results from Schiffels et al. (69) and Martiniano et al. (86). The only discrepancy is the position of the pseudo-haploid genome of BAL003, distant from the modern-day Welsh, Scottish and Northern Irish populations, as it fits present-day Belgium individuals (Fig S25). However, high uncertainties exists concerning the position of projected pseudo-haploid genomes. Martiniano et al. (88) could empirically compare bias within the same genomes either pseudo-haploidised or imputed from 2X genome coverage, and found that even if imputation bias exists, pseudo-haploid bias is stronger and the main process could be excess affinity towards the reference genome (97). The ‘reference bias’ might impact pseudo-haploid genome affinities in different unpredictable ways especially when comparing genomes reconstructed from diverse coverage.

**Inferring phenotypes from imputed genotypes**

We investigated loci associated with selective sweeps in BAL003 and LUN004 based on the imputed genotypes from positions described in the HIris-PlexS system related to skin, hair and eyes pigmentation (98) and loci associated with lactase persistence (99).

Individual BAL003 had brown/dark brown hair, blue eyes and pale to intermediate skin pigmentation, while LUN004 had blond/dark blond hair, blue eyes and pale to intermediate skin pigmentation (S12 Table). Both samples carried genetic variants associated with the ability to digest lactose in adulthood (S12 Table).

**S1.5 Reference dataset compiled with the imputed published ancient genomes and newly generated early medieval genomes from Pictland.**

A total of 1,764 individuals from 18 populations (excluding admixed African Ancestry in south-west USA (ASW) and African Caribbean in Barbados (ACB) populations) of the 1000 Genomes project phase 3 release 5 ([ftp.1000genomes.ebi.ac.uk/vol1/ftp/release/20130502/](http://ftp.1000genomes.ebi.ac.uk/vol1/ftp/release/20130502/)) were included as a worldwide reference panel. Multiallelic SNPs were removed with GATK v3.8 SelectVariants (100). Chromosomes X and Y, indels and SNPs within the strict mappability mask (<http://ftp.1000genomes.ebi.ac.uk/vol1/ftp/release/20130502/supporting/accessible_genome_masks/>) were excluded using VCFtools v0.1.15 (101), resulting in 59,145,432 variants.

A European (EU) reference panel was constructed from individuals included in the International Multiple Sclerosis Genetics Consortium & The Wellcome Trust Case Control Consortium 2 (EGAD00000000120) genotyped on the Human670-QuadCustom v1 chip (102). A UK reference panel was constructed from individuals included in the People of the British Isles study (EGAD00010000632) genotyped on the Human1-2M-DuoCustom chip (103). We converted the data from the original Oxford GEN format to the Plink Binary format, filtering for positions with genotype likelihood >0.9 using PLINK v2.0 (104,105). Of these, 10,299 individuals and 475,806 SNPs from the EU dataset and 2,578 individuals and 870,170 SNPs from the UK dataset passed the Wellcome Trust Case Control Consortium 2 (WTCCC2) quality control (106). Non-European individuals with European ancestry from Australia, New Zealand, and the US were removed from the EU dataset.

The imputed ancient genomes and the 1000 genomes phase 3 dataset SNPs are defined using the *plus* strand nomenclature, and the EU and UK datasets are defined using the TOP strand nomenclature. After checking that the SNPs from the EU and UK datasets were accurately defined on the TOP strand, we used the strand file information of the two genotyping chips to flip positions from the *minus* strand to the *plus* strand (<http://www.well.ox.ac.uk/~wrayner/strand/>) using PLINK v2.0 (104,105). Some positions did not match the 1000 genomes phase 3 dataset, likely caused by a discrepancy in the version used to define whether an SNP is on the *plus* or *minus* strand. PLINK can detect strand mismatches except for ambiguous SNPs (A/T and C/G). So, ambiguous SNPs were removed from the EU and UK datasets and the remaining mismatching SNPs positions detected by PLINK were flipped. For each genotyping chip, SNPs that did not reach the required threshold for mapping to the genome (.miss file) or that had more than one high-quality match (.multiple file) were excluded (<http://www.well.ox.ac.uk/~wrayner/strand/>). The SNP positions were lifted from NCBI36/hg18 to GRCh37/hg19 using LiftOver (107) and deleted positions in GRCh37/hg19 were excluded.

We applied an extra set of quality control to the world populations and EU and UK datasets using PLINK v2.0. Individuals with missingness >10% (--mind 0.1), sites with genotype missingness >2% (--geno 0.02), or those which deviated from Hardy-Weinberg equilibrium using a cut-off p-value of 1.10^-6^ (--hwe) were removed. The HLA region on chromosome 6 (chr6:22915594-37945593) was removed, as the EU dataset includes cases and control cases for multiple sclerosis, a disease strongly associated with the HLA region. Individuals with heterozygosity (--het) deviating from 3 median absolute deviations from their population median (measured per country) were excluded. The final datasets comprised 8,019 individuals and 457,287 SNPs for the EU dataset, 2,000 individuals and 796,718 SNPs for the UK dataset and 1,764 individuals and 57,421,294 SNPs for the world population dataset.

Imputed ancient genomes from (87) were used. We selected 252 unrelated individuals with a coverage >0.7X. Genotypes with genotype likelihood ≥0.99 using PLINK 2.0 (104,105) were retained, resulting in 27,904,557 SNPs.

**S1.6 Identity-by-Descent and Homozygosity-By-Descent**

**Methods**

The identification of Identity-By-Descent (IBD) and Homozygosity-By-Descent (HBD) segments was carried out using RefinedIBD (108). RefinedIBD compares small windows for similarity and sums them to find the full IBD or HBD segments. RefinedIBD inferred genetic positions from the HapMap GrCh37 genetic maps (<http://bochet.gcc.biostat.washington.edu/beagle/genetic_maps/>). The window size was set to 3 cM. Too large values may result in missing short segments of IBD or HBD, while too small values will prohibitively increase computation time. The minimal size for a segment to be considered shared by IBD or HBD is 1 cM, as in Margaryan et al. (109). We decided to consider segments >1 cM as shared by IBD since 1 cM corresponds to the theorical average timespan between the oldest samples from the Iron Age and present-day populations. Indeed, a common ancestor $n$ generations in the past (2$n$ meiosis) results on average in $100/2n$ cM segment length (110). Thus, IBD blocks longer than ~1 cM derive from common genetic ancestors living ~50 generations in the past, or ~1,500 years ago; this assumes an average human generation time of 30 years (111), which correspond approximately to the Late Iron Age period until the present. However, this is a mere approximation and even in a Wright-Fisher population, the distribution of genetic ancestors contributing to IBD blocks of a given length is wide (112). In reality, the population size hugely affect the distribution of IBD length and the coalescence time of a given IBD block length (113). The smaller the population size is, the more likely two chromosomal segments will be in IBD, and these segments will descent from a more recent common genetic ancestor.

IBD and HBD segments overlapping the centromere and telomeres were excluded because of the poor assembly quality of these regions. The cytobands from the UCSC genome annotation database were employed to define the centromeres coordinates (<http://hgdownload.cse.ucsc.edu/goldenPath/hg19/database/cytoBand.txt.gz>) (107). The UCSC table was used to define the telomeres coordinates, which were defined as the first and last 10,000 bp of the chromosome (114). The LOD score was calculated by trimming 0.15 cM at the end of a shared haplotype, and IBD and HBD segments with LOD >3 were kept. The LOD score corresponds to the log of the odds, i.e., the chance that two genes are linked together, by comparing the likelihood of obtaining the test data if the two loci are indeed linked to the likelihood of observing the same data by chance. LOD = 3 indicates approximately 1,000-to-one odds that the two loci are linked. We then merged segments having at most one discordant homozygote and <0.6 cM apart, to remove breaks induced by haplotype phasing and genotype errors.

The total number and total length of shared IBD segments were then calculated. To avoid sample size bias, we randomly selected the same number of modern individuals across populations. Four sets of IBD comparisons were used:

1) modern European populations, based on the mean and 95% confidence interval (CI) using 100 bootstraps based on 44 randomly drawn individuals per modern population (the minimum sample size among the European populations),

2) modern European populations compared to ancient individuals, based on the mean and 95% CI using 100 bootstraps based on 44 randomly drawn individuals per modern population,

3) modern UK populations compared to ancient individuals from Britain, using 17 random individuals per population (defined by county), based on the mean and 95% CI using 100 bootstraps based on 17 randomly drawn individuals per modern UK population (minimum sample size among the UK populations),

4) ancient individuals.

Additionally, we generated interpolated frequency maps of the total number of shared IBD between modern UK populations and ancient individuals/populations from Britain with QGIS v3.14.1 (115) using distance coefficient P = 2 and pixel size = 0.01. We used the county town as a proxy for the county geographic coordinate.

**IBD distribution across time and space**

As expected, IBD segment length is negatively correlated with the time between two pairwise samples (S23 Fig). We explored pairwise IBD segment sharing between different combinations of ancient and modern individuals using the total number and length and different thresholds of IBD segment length (1, 4 and 6 cM), interpreted as the number of shared common ancestors at different time scales (on average <2,000, <400 and <250 years ago respectively), taking a generation time of 30 years (111). For the interpretation of shared common ancestors between Iron Age or early medieval individuals and modern individuals, the threshold >1 cM is more appropriate as it corresponds with common ancestors living during the Iron Age or early medieval period.

The observed pattern of shared IBD segments among modern Europeans replicates previous findings (106). The Orcadians and Finnish populations show the highest number of intra-population shared IBD segments, interpreted as mainly reflecting small population size (S19 Fig). In particular, the results highlight that the Orcadians share more IBD segments >6 cM, relative to the Finnish, reflecting a high rate of recent shared common ancestors (S19D Fig). The Finnish population shares the most IBD segments >1 and >4 cM, likely reflecting a past small population size relative to the Orcadians (S19B and D Fig).

**Evidence for European ancestry in a Romano-British individual from Driffield terrace, Yorkshire.**

Our results show that individual 6DT3 buried in Yorkshire and dated from the Roman period is not from the same population as the bulk of the Iron Age and Roman period individuals from England (‘pop12’, S27 Fig). However, he is from the same genetic population as two early medieval people from England, and central Europeans and Scandinavians from the Iron Age and early medieval period (‘pop11’, S27Fig). This same individual shares a high proportion of >1 cM IBD segments with modern individuals from the UK, Scandinavia and Belgium (Fig 3). Population genetics study of the only ancient genomes dated from the Roman period in Britain concludes that the Roman occupation did not occur with population replacement or gene flow from mainland Europe (56). We here present, for the first time, direct evidence for an individual with central European/Scandinavian-related ancestry who lived in the British Isles during the Roman period. He was likely not a first-generation migrant, given the fit with the admixed European-like and pre-medieval period-like groups from England. This result is consistent with the archaeology suggesting that small-scale migrations throughout the Empire were common and manifested also in Britain (117). Furthermore, the ancestry is indistinguishable from that of early medieval peoples from England, which has important implications. That is, central European-related ancestry in modern individuals from Britain was initially attributed solely to Anglo-Saxon migrations (56,69,118). As Britain's most Romanised areas also correspond to the main regions of Anglo-Saxon settlement, Anglo-Saxon and Roman population movements may have both contributed to a similar pattern of western and central European admixture. Thus, gene flow responsible for the observed pattern of western and central European genetic affinities in the British Isles could have started before and during the Roman period, resulting in an over-estimation of the genetic-based Anglo-Saxon migration rate.

**References**

1. Millar C, Ross D, Harden G. Balintore (Fearn p). In: Discovery and excavation in Scotland. Proudfoot, E.V.W. The Council for British Archaeology, Scotland; 1985. p. 23.

2. Sheridan A, Armit I, Reich D, Booth T, Bernardos R, Barnes I, et al. A summary round-up list of Scottish archaeological human remains that have been sampled/analysed for DNA as of January 2019. Discovery and Excavation in Scotland. 2019;19:227–50.

3. Sánchez-Quinto F, Malmström H, Fraser M, Girdland-Flink L, Svensson EM, Simões LG, et al. Megalithic tombs in western and northern Neolithic Europe were linked to a kindred society. Proceedings of the National Academy of Sciences. 2019;116(19):9469–74.

4. Greig C, Greig M, Ashmore P, Campbell-Wilson M, Lorimer D, Smart I, et al. Excavation of a cairn cemetery at Lundin Links, Fife, in 1965-6. Proceedings of the Society of Antiquaries of Scotland. 2000;130:585–636.

5. Maldonado A. Burial in Early Medieval Scotland: New Questions. Medieval Archaeology. 2013;57(1):001–34.

6. Mitchell J, Noble G. The Monumental Cemeteries of Northern Pictland. Medieval Archaeology. 2017;61(1):1–40.

7. Longin R. New Method of Collagen Extraction for Radiocarbon Dating. Nature. 1971;230(5291):241–2.

8. Collins MJ, Galley P. Towards and optimal method of archaeological collagen extraction: the influence of pH and grinding. Ancient Biomolecules. 1998;2(2/3):209–23.

9. Britton K, Müldner G, Bell M. Stable isotope evidence for salt-marsh grazing in the Bronze Age Severn Estuary, UK: Implications for palaeodietary analysis at coastal sites. Journal of Archaeological Science. 2008;35:2111–8.

10. van Klinken GJ. Bone Collagen Quality Indicators for Palaeodietary and Radiocarbon Measurements. Journal of Archaeological Science. 1999;26(6):687–95.

11. Sayle KL, Brodie CR, Cook GT, Hamilton WD. Sequential measurement of δ15N, δ13C and δ34S values in archaeological bone collagen at the Scottish Universities Environmental Research Centre (SUERC): A new analytical frontier. Rapid Communications in Mass Spectrometry. 2019;33(15):1258–66.

12. Talamo S, Richards M. A Comparison of Bone Pretreatment Methods for AMS Dating of Samples >30,000 BP. Radiocarbon. 2011 ed;53(3):443–9.

13. Talamo S, Nowaczewska W, Picin A, Vazzana A, Binkowski M, Bosch MD, et al. A 41,500 year-old decorated ivory pendant from Stajnia Cave (Poland). Scientific Reports. 2021;11(1):1–11.

14. Brown TA, Nelson DE, Vogel JS, Southon JR. Improved Collagen Extraction by Modified Longin Method. Radiocarbon. 1988;30(2):171–7.

15. Brock F, Ramsey CB, Higham T. Quality Assurance of Ultrafiltered Bone Dating. Radiocarbon. 2007;49(2):187–92.

16. Kromer B, Lindauer S, Synal HA, Wacker L. MAMS – A new AMS facility at the Curt-Engelhorn-Centre for Achaeometry, Mannheim, Germany. Nuclear Instruments and Methods in Physics Research Section B: Beam Interactions with Materials and Atoms. 2013;294:11–3.

17. Reimer PJ, Austin WEN, Bard E, Bayliss A, Blackwell PG, Bronk Ramsey C, et al. The IntCal20 Northern Hemisphere Radiocarbon Age Calibration Curve (0–55 cal kBP). Radiocarbon. 2020;62(4):725–57.

18. Ramsey CB. Bayesian Analysis of Radiocarbon Dates. Radiocarbon. 2009;51(1):337–60.

19. Stuiver M, Polach HA. Discussion Reporting of 14 C Data. Radiocarbon. 1977;19(3):355–63.

20. Stuiver M, Reimer PJ. A Computer Program for Radiocarbon Age Calibration. Radiocarbon. 1986;28(2B):1022–30.

21. Stuiver M, Reimer PJ. Extended 14 C Data Base and Revised CALIB 3.0 14 C Age Calibration Program. Radiocarbon. 1993;35(1):215–30.

22. Meyer M, Kircher M. Illumina sequencing library preparation for highly multiplexed target capture and sequencing. Cold Spring Harbor Protocols. 2010;5(6).

23. Kircher M, Sawyer S, Meyer M. Double indexing overcomes inaccuracies in multiplex sequencing on the Illumina platform. Nucleic Acids Research. 2012;40(1):e3.

24. Illumina. Minimizing Index Hopping [Internet]. 2022 [cited 2022 Mar 4]. Available from: https://www.illumina.com/techniques/sequencing/ngs-library-prep/multiplexing/index-hopping.html

25. Valk T van der, Vezzi F, Ormestad M, Dalén L, Guschanski K. Index hopping on the Illumina HiseqX platform and its consequences for ancient DNA studies. Molecular Ecology Resources. 2020;20(5):1171–81.

26. Meyerhans A, Vartanian JP, Wain-Hobson S. DNA recombination during PCR. Nucleic Acids Research. 1990;18(7):1687–91.

27. Pääbo S, Irwin DM, Wilson AC. DNA damage promotes jumping between templates during enzymatic amplification. Journal of Biological Chemistry. 1990;265(8):4718–21.

28. Costello M, Fleharty M, Abreu J, Farjoun Y, Ferriera S, Holmes L, et al. Characterization and remediation of sample index swaps by non-redundant dual indexing on massively parallel sequencing platforms. BMC Genomics. 2018;19(1):332.

29. Raghavan M, Skoglund P, Graf KE, Metspalu M, Albrechtsen A, Moltke I, et al. Upper Palaeolithic Siberian genome reveals dual ancestry of native Americans. Nature. 2014;505(7481):87–91.

30. Renaud G, Slon V, Duggan AT, Kelso J. Schmutzi: Estimation of contamination and endogenous mitochondrial consensus calling for ancient DNA. Genome Biology. 2015;16(1):1–18.

31. Furtwängler A, Reiter E, Neumann GU, Siebke I, Steuri N, Hafner A, et al. Ratio of mitochondrial to nuclear DNA affects contamination estimates in ancient DNA analysis. Scientific Reports. 2018;8(14075).

32. Lazaridis I, Patterson N, Mittnik A, Renaud G, Mallick S, Kirsanow K, et al. Ancient human genomes suggest three ancestral populations for present-day Europeans. Nature. 2014;513(7518):409–13.

33. Li H, Handsaker B, Wysoker A, Fennell T, Ruan J, Homer N, et al. The Sequence Alignment/Map format and SAMtools. Bioinformatics. 2009;25(16):2078–9.

34. Patterson N, Moorjani P, Luo Y, Mallick S, Rohland N, Zhan Y, et al. Ancient admixture in human history. Genetics. 2012;192(3):1065–93.

35. Mallick S, Li H, Lipson M, Mathieson I, Gymrek M, Racimo F, et al. The Simons Genome Diversity Project: 300 genomes from 142 diverse populations. Nature. 2016;538(7624):201–6.

36. Allentoft ME, Sikora M, Sjögren KG, Rasmussen S, Rasmussen M, Stenderup J, et al. Population genomics of Bronze Age Eurasia. Nature. 2015;522(7555):167–72.

37. Amorim CEG, Vai S, Posth C, Modi A, Koncz I, Hakenbeck S, et al. Understanding 6th-century barbarian social organization and migration through paleogenomics. Nature Communications. 2018;9(1):1–11.

38. Antonio ML, Gao Z, Moots HM, Lucci M, Candilio F, Sawyer S, et al. Ancient Rome: A genetic crossroads of Europe and the Mediterranean. Science. 2019;366(6466):708–14.

39. Brace S, Diekmann Y, Booth TJ, Dorp L van, Faltyskova Z, Rohland N, et al. Ancient genomes indicate population replacement in Early Neolithic Britain. Nature Ecology and Evolution. 2019;3(5):765–71.

40. Broushaki F, Thomas MG, Link V, López S, Dorp L van, Kirsanow K, et al. Early Neolithic genomes from the eastern Fertile Crescent. Science. 2016;353(6298):499–503.

41. Cassidy LM, Martiniano R, Murphy EM, Teasdale MD, Mallory J, Hartwell B, et al. Neolithic and Bronze Age migration to Ireland and establishment of the insular Atlantic genome. Proceedings of the National Academy of Sciences. 2016;113(2):368–73.

42. Damgaard P, Martiniano R, Kamm J, Moreno-Mayar JV, Kroonen G, Peyrot M, et al. The first horse herders and the impact of early Bronze Age steppe expansions into Asia. Science. 2018;360(6396):eaar7711.

43. Damgaard PB, Marchi N, Rasmussen S, Peyrot M, Renaud G, Korneliussen T, et al. 137 ancient human genomes from across the Eurasian steppes. Nature. 2018;557(7705):369–74.

44. Fu Q, Posth C, Hajdinjak M, Petr M, Mallick S, Fernandes D, et al. The genetic history of Ice Age Europe. Nature. 2016;534(7606):200–5.

45. Gamba C, Jones ER, Teasdale MD, McLaughlin RL, Gonzalez-Fortes G, Mattiangeli V, et al. Genome flux and stasis in a five millennium transect of European prehistory. Nature Communications. 2014;5(1):1–9.

46. González-Fortes G, Jones ER, Lightfoot E, Bonsall C, Lazar C, Grandal-d’Anglade A, et al. Paleogenomic Evidence for Multi-generational Mixing between Neolithic Farmers and Mesolithic Hunter-Gatherers in the Lower Danube Basin. Current Biology. 2017;27(12):1801-1810.e10.

47. Hofmanová Z, Kreutzer S, Hellenthal G, Sell C, Diekmann Y, Díez-Del-Molino D, et al. Early farmers from across Europe directly descended from Neolithic Aegeans. Proceedings of the National Academy of Sciences of the United States of America. 2016;113(25):6886–91.

48. Järve M, Saag L, Scheib CL, Pathak AK, Montinaro F, Pagani L, et al. Shifts in the Genetic Landscape of the Western Eurasian Steppe Associated with the Beginning and End of the Scythian Dominance. Current Biology. 2019;29(14):2430-2441.e10.

49. Jones ER, Gonzalez-Fortes G, Connell S, Siska V, Eriksson A, Martiniano R, et al. Upper Palaeolithic genomes reveal deep roots of modern Eurasians. Nature Communications. 2015;6(1):1–8.

50. Krzewińska M, Kjellström A, Günther T, Hedenstierna-Jonson C, Zachrisson T, Omrak A, et al. Genomic and Strontium Isotope Variation Reveal Immigration Patterns in a Viking Age Town. Current Biology. 2018;28(17):2730-2738.e10.

51. Krzewińska M, Kılınç GM, Juras A, Koptekin D, Chyleński M, Nikitin AG, et al. Ancient genomes suggest the eastern Pontic-Caspian steppe as the source of western Iron Age nomads. Science Advances. 2018;4(10):eaat4457.

52. Lazaridis I, Mittnik A, Patterson N, Mallick S, Rohland N, Pfrengle S, et al. Genetic origins of the Minoans and Mycenaeans. Nature. 2017;548(7666):214–8.

53. Lazaridis I, Nadel D, Rollefson G, Merrett DC, Rohland N, Mallick S, et al. Genomic insights into the origin of farming in the ancient Near East. Nature. 2016;536(7617):419–24.

54. Lipson M, Szécsényi-Nagy A, Mallick S, Pósa A, Stégmár B, Keerl V, et al. Parallel palaeogenomic transects reveal complex genetic history of early European farmers. Nature. 2017;551(7680):368–72.

55. Malmström H, Günther T, Svensson EM, Juras A, Fraser M, Munters AR, et al. The genomic ancestry of the Scandinavian Battle Axe Culture people and their relation to the broader Corded Ware horizon. Proceedings of the Royal Society B: Biological Sciences. 2019;286(1912).

56. Martiniano R, Caffell A, Holst M, Hunter-Mann K, Montgomery J, Müldner G, et al. Genomic signals of migration and continuity in Britain before the Anglo-Saxons. Nature Communications. 2016;7:1–8.

57. Mathieson I, Alpaslan-Roodenberg S, Posth C, Szécsényi-Nagy A, Rohland N, Mallick S, et al. The genomic history of southeastern Europe. Nature. 2018;555(7695):197–203.

58. Mathieson I, Lazaridis I, Rohland N, Mallick S, Patterson N, Roodenberg SA, et al. Genome-wide patterns of selection in 230 ancient Eurasians. Nature. 2015;528(7583):499–503.

59. Mittnik A, Massy K, Knipper C, Wittenborn F, Friedrich R, Pfrengle S, et al. Kinship-based social inequality in Bronze Age Europe. Science. 2019;366(6466):731–4.

60. Mittnik A, Wang CC, Pfrengle S, Daubaras M, Zariņa G, Hallgren F, et al. The genetic prehistory of the Baltic Sea region. Nature Communications. 2018;9(1):1–11.

61. Moreno-Mayar JV, Vinner L, De Barros Damgaard P, De La Fuente C, Chan J, Spence JP, et al. Early human dispersals within the Americas. Science. 2018;362(6419).

62. Narasimhan VM, Patterson N, Moorjani P, Rohland N, Bernardos R, Mallick S, et al. The Formation of Human Populations in South and Central Asia. Science. 2019;365(6457):1–43.

63. Olalde I, Mallick S, Patterson N, Rohland N, Villalba-mouco V, Silva M, et al. The genomic history of the Iberian Peninsula over the past 8000 years. Science. 2019;1234(6432):1230–4.

64. Olalde I, Brace S, Allentoft ME, Armit I, Kristiansen K, Booth T, et al. The Beaker phenomenon and the genomic transformation of northwest Europe. Nature. 2018;555(7695):190–6.

65. Olalde I, Allentoft ME, Sánchez-Quinto F, Santpere G, Chiang CWK, DeGiorgio M, et al. Derived immune and ancestral pigmentation alleles in a 7,000-year-old Mesolithic European. Nature. 2014;507(7491):225–8.

66. Prendergast ME, Lipson M, Sawchuk EA, Olalde I, Ogola CA, Rohland N, et al. Ancient DNA reveals a multistep spread of the first herders into sub-Saharan Africa. Science. 2019;365(44).

67. Rodríguez-Varela R, Günther T, Krzewińska M, Storå J, Gillingwater TH, MacCallum M, et al. Genomic Analyses of Pre-European Conquest Human Remains from the Canary Islands Reveal Close Affinity to Modern North Africans. Current Biology. 2017;27(21):3396-3402.e5.

68. Saag L, Laneman M, Varul L, Malve M, Valk H, Razzak MA. The Arrival of Siberian Ancestry Connecting the Eastern Baltic to Uralic Speakers Further East. 2019;29(10):1701–11.

69. Schiffels S, Haak W, Paajanen P, Llamas B, Popescu E, Loe L, et al. Iron Age and Anglo-Saxon genomes from East England reveal British migration history. Nature Communications. 2016;7:1–9.

70. Seguin-Orlando A, Korneliussen TS, Sikora M, Malaspinas AS, Manica A, Moltke I, et al. Genomic structure in Europeans dating back at least 36,200 years. Science. 2014;346(6213):1113–8.

71. Sikora M, Pitulko V V., Sousa VC, Allentoft ME, Vinner L, Rasmussen S, et al. The population history of northeastern Siberia since the Pleistocene. Nature. 2019;570(7760):182–8.

72. Sikora M, Seguin-Orlando A, Sousa VC, Albrechtsen A, Korneliussen T, Ko A, et al. Ancient genomes show social and reproductive behavior of early Upper Paleolithic foragers. Science. 2017;358(6363):659–62.

73. Skoglund P, Malmström H, Omrak A, Raghavan M, Valdiosera C, Günther T, et al. Genomic diversity and admixture differs for stone-age Scandinavian foragers and farmers. Science. 2014;344(6185):747–50.

74. Sunna Ebenesersdóttir S, Sandoval-Velasco M, Gunnarsdóttir ED, Jagadeesan A, Guðmundsdóttir VB, Thordardóttir EL, et al. Ancient genomes from Iceland reveal the making of a human population. Science. 2018;360(6392):1028–32.

75. Unterländer M, Palstra F, Lazaridis I, Pilipenko A, Hofmanová Z, Groß M, et al. Ancestry and demography and descendants of Iron Age nomads of the Eurasian Steppe. Nature Communications. 2017;8(1):1–10.

76. Veeramah KR, Rott A, Groß M, Dorp LV, López S, Kirsanow K, et al. Population genomic analysis of elongated skulls reveals extensive female-biased immigration in Early Medieval Bavaria. Proceedings of the National Academy of Sciences of the United States of America. 2018;115(13):3494–9.

77. Patterson NJ, Price AL, Reich D. Population Structure and Eigenanalysis. PLoS Genetics. 2006;2(12):e190.

78. Alexander DH, Novembre J. Fast Model-Based Estimation of Ancestry in Unrelated Individuals. Genome Research. 2009;1655–64.

79. Purcell S, Neale B, Todd-Brown K, Thomas L, Ferreira MAR, Bender D, et al. PLINK: A Tool Set for Whole-Genome Association and Population-Based Linkage Analyses. The American Journal of Human Genetics. 2007;81(3):559–75.

80. Haak W, Lazaridis I, Patterson N, Rohland N, Mallick S, Llamas B, et al. Massive migration from the steppe was a source for Indo-European languages in Europe. Nature. 2015;522(7555):207–11.

81. Petr M, Vernot B, Kelso J. admixr—R package for reproducible analyses using ADMIXTOOLS. Bioinformatics. 2019;35(17):3194–5.

82. Harney É, Patterson N, Reich D, Wakeley J. Assessing the Performance of qpAdm: A Statistical Tool for Studying Population Admixture. bioRxiv. 2020.

83. Ebenesersdóttir SS, Sandoval-Velasco M, Gunnarsdóttir ED, Jagadeesan A, Guðmundsdóttir VB, Thordardóttir EL, et al. Ancient genomes from Iceland reveal the making of a human population. Science. 2018;360(6392):1028–32.

84. Patterson N, Isakov M, Booth T, Büster L, Fischer CE, Olalde I, et al. Large-scale migration into Britain during the Middle to Late Bronze Age. Nature. 2021;601(7894):1–14.

85. Broad Institute. Picard tools. http://broadinstitute.github.io/picard/. 2016.

86. Martiniano R, Caffell A, Holst M, Hunter-Mann K, Montgomery J, Müldner G, et al. Genomic signals of migration and continuity in Britain before the Anglo-Saxons. Nature Communications. 2016;7:1–8.

87. Margaryan A, Lawson DJ, Sikora M, Racimo F, Rasmussen S, Moltke I, et al. Population genomics of the Viking world Scandinavian ancestry and Viking Age origins Check for updates. Nature. 2020;585(7825):390.

88. Martiniano R, Cassidy LM, Ó’Maoldúin R, McLaughlin R, Silva NM, Manco L, et al. The population genomics of archaeological transition in west Iberia: Investigation of ancient substructure using imputation and haplotype-based methods. PLoS Genetics. 2017;13(7):1–24.

89. Rubinacci S, Ribeiro DM, Hofmeister RJ, Delaneau O. Efficient phasing and imputation of low-coverage sequencing data using large reference panels. Nature Genetics. 2021;53(1):120–6.

90. Li N, Stephens M. Modeling Linkage Disequilibrium and Identifying Recombination Hotspots Using Single-Nucleotide Polymorphism Data. Genetics. 2003;165(4):2213–33.

91. Browning SR, Browning BL. Rapid and Accurate Haplotype Phasing and Missing-Data Inference for Whole-Genome Association Studies By Use of Localized Haplotype Clustering. The American Journal of Human Genetics. 2007;81(5):1084–97.

92. Browning BL, Zhou Y, Browning SR. A One-Penny Imputed Genome from Next-Generation Reference Panels. American Journal of Human Genetics. 2018;103(3):338–48.

93. Depristo MA, Banks E, Poplin R, Garimella K V., Maguire JR, Hartl C, et al. A framework for variation discovery and genotyping using next-generation DNA sequencing data. Nature Genetics. 2011;43(5):491–501.

94. Auton A, Abecasis GR, Altshuler DM, Durbin RM, Abecasis GR, Bentley DR, et al. A global reference for human genetic variation. Nature. 2015;526(7571):68–74.

95. Danecek P, Bonfield JK, Liddle J, Marshall J, Ohan V, Pollard MO, et al. Twelve years of SAMtools and BCFtools. GigaScience. 2021;10(2).

96. Danecek P, Schiffels S, Durbin R. Multiallelic calling model in bcftools (-m). 2014.

97. Günther T, Nettelblad C. The presence and impact of reference bias on population genomic studies of prehistoric human populations. PLoS Genetics. 2019;15(7).

98. Chaitanya L, Breslin K, Zuñiga S, Wirken L, Pośpiech E, Kukla-Bartoszek M, et al. The HIrisPlex-S system for eye, hair and skin colour prediction from DNA: Introduction and forensic developmental validation. Forensic Science International: Genetics. 2018;35:123–35.

99. Enattah NS, Sahi T, Savilahti E, Terwilliger JD, Peltonen L, Järvelä I. Identification of a variant associated with adult-type hypolactasia. Nature Genetics. 2002;30(2):233–7.

100. Van der Auwera GA, O’Connor BD. Genomics in the Cloud. Using Docker, GATK, and WDL in Terra. O’Reilly Media, Inc; 2020.

101. Danecek P, Auton A, Abecasis G, Albers CA, Banks E, DePristo MA, et al. The variant call format and VCFtools. Bioinformatics. 2011;27(15):2156–8.

102. Sawcer S, Hellenthal G, Pirinen M, Spencer CCA, Patsopoulos NA, Moutsianas L, et al. Genetic risk and a primary role for cell-mediated immune mechanisms in multiple sclerosis. Nature. 2011;476:214–9.

103. Winney B, Boumertit A, Day T, Davison D, Echeta C, Evseeva I, et al. People of the British Isles: Preliminary analysis of genotypes and surnames in a UK-control population. European Journal of Human Genetics. 2012;20(2):203–10.

104. Chang CC, Chow CC, Tellier LCAM, Vattikuti S, Purcell SM, Lee JJ. Second-generation PLINK: Rising to the challenge of larger and richer datasets. GigaScience. 2015;4(1):7.

105. Purcell S, Chang C. PLINK 2.0. 2015.

106. Strange A, Capon F, Spencer CCA, Knight J, Weale ME, Allen MH, et al. A genome-wide asociation study identifies new psoriasis susceptibility loci and an interaction betwEn HLA-C and ERAP1. Nature Genetics. 2010;42(11):985–90.

107. Kent WJ, Sugnet CW, Furey TS, Roskin KM, Pringle TH, Zahler AM, et al. The human genome browser at UCSC. Genome research. 2002;12(6):996–1006.

108. Browning BL, Browning SR. Improving the Accuracy and Efficiency of Identity-by-Descent Detection in Population Data. Genetics. 2013;194(2):459–71.

109. Browning SR. Estimation of Pairwise Identity by Descent From Dense Genetic Marker Data in a Population Sample of Haplotypes. Genetics. 2008;178(4):2123.

110. Helgason A, Hrafnkelsson B, Gulcher JR, Ward R, Stefánsson K. A Populationwide Coalescent Analysis of Icelandic Matrilineal and Patrilineal Genealogies: Evidence for a Faster Evolutionary Rate of mtDNA Lineages than Y Chromosomes. The American Journal of Human Genetics. 2003;72(6):1370–88.

111. Ringbauer H, Novembre J, Steinrücken M. Parental relatedness through time revealed by runs of homozygosity in ancient DNA. Nat Commun. 2021;12(1):5425.

112. Ralph P, Coop G. The Geography of Recent Genetic Ancestry across Europe. PLOS Biology. 2013;11(5):e1001555.

113. Karolchik D, Hinrichs AS, Furey TS, Roskin KM, Sugnet CW, Haussler D, et al. The UCSC Table Browser data retrieval tool. Nucleic Acids Research. 2004;32:D493.

114. QGIS Development Team. QGIS Geographic Information System [Internet]. 2020. Available from: http://qgis.osgeo.org

115. Ferrando-Bernal M, Morcillo-Suarez C, de-Dios T, Gelabert P, Civit S, Díaz-Carvajal A, et al. Mapping co-ancestry connections between the genome of a Medieval individual and modern Europeans. Scientific Report. 2020;10(1):6843.

116. Cunliffe B. Britain Begins. Oxford University Press; 2013.

117. Leslie S, Winney B, Hellenthal G, Davison D, Boumertit A, Day T, et al. The fine-scale genetic structure of the British population. Nature. 2015;519(7543):309–14.
