# Supplementary material for: Imputed genomes and haplotype-based analyses of the Picts of early medieval Scotland reveal fine-scale relatedness between Iron Age, early medieval and the modern people of the UK
Source: PLoS Genet. 2023 Apr 27;19(4):e1010360. doi: 10.1371/journal.pgen.1010360 (PMC10138790; doi:10.1371/journal.pgen.1010360)
Supplement: Attachment — Submitted filename: Response To Reviewers.docx [file pgen.1010360.s041.docx]

**Page and Line number of changes brought to the manuscript refer to those from the revised manuscript with changes highlighted.**

**Reviewer #1:**

Morez et al. use ancient DNA to investigate the genetics of the ancient ‘Picts’ – groups who inhabited aspects of present-day Scotland in the early medieval period. The origins of these groups of people and what language they spoke have been longstanding questions in the history of Britain, with multiple theories deriving from linguistic comparisons as well as histories written by contemporary and later historians. Even though Britain is one of the most studied areas of the world when it comes to ancient DNA, so far there has been no head-on attempt to investigate what ancient DNA can tell us about the Picts.

Morez et al. are the first to directly investigate Pictish genetics by, in the first instance producing one medium and one high coverage ancient genome from disparate parts of Pictish territory. However, the main novelty and power of their paper is in applying imputation methods to previously published data to explore fine-scale genetic affinities of Iron Age and early medieval populations of Britain in a way that has not been possible previously. They therefore produce a whole range of insights not only about the Picts, but also other ancient populations of Britain and their relationship to some of the populations who inhabit the same regions today. Morez et al. also generate mitochondrial genome data from a further 7 Pictish samples from the Lundin Links site to investigate hypotheses established from historical sources that Pictish groups were matriarchal. The diversity of maternal lineages they discover at this cemetery suggests that burial was not based on matrilineal descent. However, the authors are careful to discuss a whole range of caveats to any conclusions about social organisation in Pictish societies from a small number of samples from a single site. The paper is well-written and clear, uses appropriate methods in novel ways and includes some excellent figures. Therefore I believe is well worthy of publication. I just had a few minor comments that I think the authors could consider:

Page 2, Line 22: ‘The origins and ancestry of the Picts of early medieval Scotland (ca. AD 300-900) has been traditionally seen as a problem, prompted in part by exotic medieval origin myths, their enigmatic symbols and inscriptions, and the meagre textual evidence.’

The wording of this sentence comes across as slightly odd to me. ‘Problem’ in this context means something potentially harmful rather than a question to be resolved. I think the authors could consider rephrasing, perhaps something like, ‘There are longstanding questions about the origins and ancestry of the Picts of early medieval Scotland (ca. AD 300-900), prompted…’

‘Problem’ here refers to ‘the problem of the Picts’ by Wainwright (1955), which remains a key reference in research concerning Pictish ancestry, culture, society, *etc*. However, we understand that the reference will not be understood by everyone so we replaced it as suggested.

Page 3, Line 59: ‘The British Isles witnessed a complex cultural turnover from the Iron Age to the early medieval period. The Romans occupied part of Britain to southern Scotland from AD 43 to ca. AD 410; however, this occupation resulted in little detectable gene flow from mainland Europe (14).’

The authors use the term ‘British Isles’ throughout. Are the authors using ‘British Isles’ to refer to the islands associated with Britain and Ireland or just Britain? While ‘British Isles’ is primarily a geographic term and is strictly accurate when discussing Britain and Ireland (along with their smaller islands together), it is still considered by some to be politically loaded, particularly in the Republic of Ireland where they (understandably) take issue being referred to as ‘British’. If the authors are using this term just to refer to Britain and its associated islands, then ‘British Isles’ is a bit confusing because of this history of use – If the authors are using it this way they should probably define what they mean by it. It is up to the authors whether they want to adjust this, but alternatives which are less politically sensitive include ‘British and Irish Isles’, or just ‘Britain and Ireland’, with some definition specifying that this refers to both or either of these countries and associated smaller islands.

This is a good and insightful comment on the issues surrounding the nomenclature of the isles comprising Britain and Ireland, and we accept the suggestion and clarify this by using ‘the British and Irish Isles’ and writing out this definition at the beginning of the introduction Pages 3-4 Line 68-69.

Page 3, Line 61: ‘The Romans occupied part of Britain to southern Scotland from AD 43 to ca. AD 410; however, this occupation resulted in little detectable gene flow from mainland Europe (14).’

I think this is quite a bold statement from the authors deriving from a single study of a single Roman site, particularly as later genetic shifts might have distorted or erased any pre-early medieval gene flow. Also, the authors themselves later find evidence that this may not be strictly true in their reanalysis of the same site. Perhaps ‘…however, the single ancient DNA study of Roman Britain suggests this occupation…’

That is a very good point and we modify this sentence following the suggestion.

On a related note, we added a reference to Gretzinger *et al.* 2022 Page 4, Line 85, which analyse 460 medieval north-western Europeans and refined the pattern of migration from continental Europe into present-day England associated with the Anglo-Saxon culture.

Page 4, Line 84: ‘In the modern era perceptions of Pictish origins have varied, often according to cultural and political biases, with the Picts and their languages regarded as Germanic, Gaelic, Brittonic, Basque, and Illyrian, among other theories. In the 1950s Jackson influentially argued that the Picts spoke a non-Indo-European language and a Celtic language akin to ancient Gaulish (23,24). The current consensus is that they spoke a Celtic language closest to that of neighbouring Britons from which Cornish, Welsh, and Breton derive (25–27). However, some still argue from undeciphered inscriptions and other words that some Picts spoke an otherwise unknown language, presumably derived from a pre-Celtic population (28,29). Thus, the question remains of whether the Picts were somehow fundamentally different from their neighbours.’

I may have missed it, but I think the authors could consider mentioning a further complication, particularly when it comes to links between language and genetics - that the Patterson et al. (2021) paper found that genetic change in southern Britain produced by Late Bronze Age migrations which may have introduced Celtic languages or certain forms of Celtic to these areas, was not so influential in northern Britain.

That is definitely something very interesting to include and we have added a sentence to emphasise this, Page 5 Lines 110-112.

Page 7, Line 147: ‘but without additional resolution to determine whether this sample carries the R1b-P312/S116 haplogroup introduced to Britain by Bell Beaker peoples during the Chalcolithic, alongside Yamnaya-related ancestry (7).’

I think it would be useful for the authors to consider whether it is better to refer to steppe-related or Western Steppe Herder (WSH) ancestry rather than Yamnaya-related, as these terms are not culturaly specific and there has been some recent evidence that this ancestry may not be so much derived from Yamnaya groups (although I suppose ‘Yamnaya-related’ is still technically accurate).

We agree, this is a more accurate term. We replace any occurrence of ‘Yamnaya-related' with ‘steppe-related'.

Page 8, Line 158: ‘The PCA shows that the ancient individuals from Britain broadly fit within present-day diversity (Fig 159 2A).’

I think this sentence is a bit unclear – present-day diversity of where? Also, I think the authors (and indeed, all geneticists to some extent) could be more careful to be precise when discussing present-day populations. The present-day samples referred to here do not represent the diversity of present-day people in Britain but of people whose recent ancestors predominantly came from Britain. I appreciate this kind of thing is understood between geneticists, but increasingly there will be many non-specialists interested in this paper who sometimes get the wrong idea of what geneticists mean when referring to present-day populations. I don’t think the authors need to define what they mean by present-day groups every time they are mentioned, perhaps just once on their first mention.

This is again a very good point. We modified this at the first appearance in the text Page 9 Lines 180-181 and more developed answer in Material and Methods Page 22 Line 508.

Page 9, Line 194: ‘This implies that we cannot consider individuals from Pictland a homogenous genetic group but instead a complex mixture of contemporary genetic ancestries.’

I think ‘implies’ might be the wrong word here as the authors make this observation directly. ‘Suggests’ or ‘indicates’ might be better, although I think beginning the sentence from ‘We’ would also be justifiable and make a stronger statement.

Good point and we amended this sentence following these suggestions, Page 10, Lines 200-202.

Page 10, Line 213: ‘In fact, recent research show that Bronze Age populations in Orkney were differentiated from their counterparts on mainland Britain due to retention of male Neolithic ancestry (Y-chromosomal haplogroup I2), while the R1b haplogroup associated with Bell Beaker expansion largely replaced the I2 haplogroups in the rest of Britain (13), implying that local ancestry may have persisted also into the Iron Age and early medieval period’

This is a very interesting idea. However, if there had been significant retention of ancestry from Neolithic groups in Orkney into the Bronze Age beyond paternal lineages, wouldn’t we see this in the whole ancestry of Bronze Age Orcadians? From what I understand, Dulias et al. (2022) found the ancestry turnover in Orkney was just as large as in mainland Scotland. Or are the authors speculating that there may have been remnant local pockets of Neolithic-derived ancestry amongst unsampled communities in Bronze Age Orkney? If so, I think this could be clearer. If not, would this then suggest significant genetic drift in Orkney through the Bronze Age is more likely to be responsible? In fact, Dulias et al. (2022) observed such Bronze Age drift directly.

That is correct. They estimate that 95% genome-wide replacement could be achieved within 5 generations (100-150 years) in a patrilocal system with indigenous fathers (of Neolithic descent) and migrant mothers (of continental European ancestry), assuming a small population size at the onset of the Bronze Age (as shown empirically). The long HBD (or RoH) seen in one ancient Orcadian (10 cM, S26 Fig) and the high number of IBD segments shared within the modern Orcadians corroborates the small population size in Orkney. Lawson, Dorp and Falush, 2018 demonstrated formally that bottlenecks can mimic admixture component increase via gene flow in an ADMIXTURE profile (see their fig 2) (added Page 11, L243-244). While the idea of unsampled pockets of Neolithic ancestry is intriguing, it is merely speculative and we have modified this sentence to exclude this suggestion. Instead, we have emphasised the empirical evidence for small population size and genetic drift described in Dulias *et al.* 2022.

Page 10, Line 220: ‘This is consistent with our results that show a high proportion of shared IBD segments among modern Orcadians (>1 to >6 cM, S19 Fig), meaning they share a high proportion of recent common ancestors relative to most modern European populations, typical of small or genetically isolated populations.’

This is an excellent, brief explainer of what IBD effectively means in practice, nice work, very useful!

We thank the reviewer for this comment!

Page 11, Line 243: ‘This pattern suggests that Anglo-Saxon ancestry expanded out of south-eastern England followed by admixture with local populations, which is a scenario consistent with previous research (11,14,17,42,43).’

I think the authors could consider whether discussion of ‘Anglo-Saxon ancestry’ is appropriate here. ‘Anglo-Saxon’ is a cultural grouping and one which as far as we know seems to encompass people with ancestries derived both from northern continental Europe and Roman Britain (Schiffels et al. 2016). Therefore discussing ‘Anglo-Saxon ancestry’ seems imprecise to me as well as having some political potency. Something along the lines of ‘This pattern suggests ancestry from northern continental Europe associated with Saxon migrations expanded out of south-eastern Britain…’ might reflect the situation more precisely without making such a direct link between culture and DNA.

Yes, we agree this is a more accurate way to describe this finding.

Page 12, Line 262: ‘Alternatively, the ancestors of BAL003 and LUN004 share more IBD segments with present-day people from western Scotland, Wales, and Northern Ireland because they (or their direct ancestors) migrated from these regions but did not contribute substantially to later generations via admixture with local groups in eastern Scotland.’

Given that some of the historical sources make clear a connection between the Picts and Ireland (as the authors discuss briefly I their introduction), the primary thing that feels to be missing from this paper is a fuller comparison of Picts against populations from Ireland. This especially true given the author’s interesting finding that BAL003 and LUN004 have affinities to present-day people from northern Ireland and other place in Britain (west of Wales, Argyll) that have been influenced by Irish migration in more recent times. The authors do hint at this possibility in posing a scenario where both Pictish individuals were recent migrants or descendants of migrants from further west, but couldn’t this be explored in more detail? I appreciate that there is little relevant ancient data from Ireland, but couldn’t the present-day Irish data from the Gilbert et al. (2017) paper help here? For instance if BAL003 and LUN004 share ibd segments with mainly northern Ireland, this may suggest that sharing between Pictish samples and Ireland might be down to more recent interactions between northern Ireland and southwestern Scotland. If the Pictish samples shared affinities with people on the island of Ireland more generally, this may be more suggestive of an ancient Irish influence. Alternatively, are the Bronze Age samples from Rathlin island too temporally distant to be of use here? Could comparison of the authors’ Pictish genomes against Bronze Age samples from Ireland (with an assumption of some level of continuity into the Iron Age) and Iron Age samples from Scotland help to tease out any ancient Irish-specific genetic influence?

We originally intended to include additional reference data from the Republic of Ireland. However, despite repeated attempts to gain access to the Byrne *et al.* (2018) dataset (which is the one used in Gilbert *et al.* (2019)) by contacting the named data curators, we never received replies to our request. Due to the extremely long time required to process and analyse the data presented in our paper, we had to make the decision to proceed with the current iteration of the modern reference data/panel.

We decided that the Rathlin genomes are likely too distant relative to Iron Age groups from Ireland to be of immediate relevance here. Indeed, Rathlin could be closer to Bronze Age groups from Scotland or Ireland, especially given the Late Bronze Age (Patterson *et al.,* 2021) and Iron Age/Early Medieval influx of migrants (Martiniano *et al.*, 2016; Schiffels *et al.*, 2016; Gretzinger *et al.*, 2022) was asymmetrical in the British and Irish Isles. The Bronze to Iron Age transition is too poorly characterised in terms of genetic shifts that we prefer to be careful interpreting such signals. For example, a putative relatively high affinity between the Picts and Rathlin could mean either an ancient Irish influence, a southwest Scotland influence, or continuity with Bronze Age central/east Scotland.

Page 13, Line 286: ‘Therefore, the marked genetic differentiation between the Orkney and mainland Britain is not only a result of Scandinavian admixture, as previously hypothesised (11,42,50–53) but also pronounced genetic continuity that persisted for at least 2,000 years.’

Typo - ‘the Orkney’

Accepted and also corrected one occurrence in the abstract.

Page 13, Line 297: ‘6DT3 also share relatively more IBD segments >1 cM with the present-day population from Scandinavia, Belgium and the UK (Fig 3), suggesting that Scandinavian-like ancestry could have spread to the British Isles before the Anglo-Saxon period.’

As the authors are discussing Britain, rather than just England, I think they could consider whether it would be better to stick to ‘early medieval’ rather than use ‘the Anglo-Saxon period’. The authors tend to use ‘early medieval’ period throughout the paper and so using it here would be more consistent.

Accepted. We discussed that extensively and for us both are correct if ‘Anglo-Saxon’ refers to a *period* and not an *ethic* or an *ancestry*.

Page 14, Line 300 ‘Seven mtDNA genomes were retrieved at Lundin Links, which allows us to answer questions about the Pictish social organisation reflected in the individuals interred at the site.’

I’m not sure if the authors ‘answer’ questions about Pictish social organisation here – maybe ‘…address questions…’?

I am impressed by the way the authors qualify their statements about inferring social organisation from ancient genomic data and go into some of the complexities that make it difficult to be sure of their interpretations. Having said that, I do feel one thing that is missing or at least not as prominent as it could be is that the authors (as in other archaeogenetics papers) seem to take it for granted that cemeteries include people who cohabited in life and so provide an insight into who stayed and who left a particular settlement. For instance, there could be a situation where bodies are returned to their natal communities despite having lived their lives within the communities they married into. I think it is totally reasonable to discuss social organisation with this assumption in mind but I think the assumption has to be made explicit to show what interpretive steps are being taken.

Very good point. We clarify this in the sentence *(italics):* “This result suggests the individuals buried at Lundin Links were unlikely to have been practicing matrilocality; *this assumes that this cemetery is a snapshot of the community near Lundin Links, and individuals did not return to their natal place for burial after having lived in their marital community.*”

Page 14, Line 305: ‘It is worth noting that the two individuals retrieved from the horned cairns complex individuals (S1 Fig) show evidence of familial links based on skeletal morphology (LUN001 and LUN009) (35).’

I think it would be useful here for the authors to give some brief detail on what skeletal traits suggested a family link just to make it easier for a reader to assess the strength of this evidence.

Accepted. We clarified this part of the manuscript by adding a description of the skeletal traits in question.

Page 14, Line 314: ‘Thus, this is unlikely that the community at Lundin Links followed a matrilineal inheritance system, which challenges older arguments for matrilineal succession among Pictish rulers (59).’

As I’ve discussed above, I think an important caveat to the authors’ statement is ‘Insofar as funerary treatment can tell us about social organisation in life…’.

Good point, accepted.

Page 15, Line 343: ‘On a more local level, our mtDNA analysis of individuals interred at Lundin Links is inconsistent with matrilocality. This finding argues against the older hypothesis that Pictish succession was based on a matrilineal system, assuming that wider Pictish society was organised in such manner.’

Given the limitations to this interpretation the authors themselves cover in their discussion, I think these sentences are a bit strong and require some qualification – perhaps a ‘Tentatively…’ at the beginning of the second sentence. Also I’m not sure if the finding ‘argues’ against the hypotheses, maybe ‘goes against’ would be a better fit. There may be in ‘a’ missing from ‘such a manner’.

Supplementary Information:

‘We observed an increase of Anatolian Neolithic ancestry in the Iron Age individual buried in southern England (13.4% ± 4.7 SE) , compared to an Iron Age individual buried in northern England (6.60% ± 17.5 SE) (52)’

Has the second of these figures percentages/standard errors been switched?

The second of these figures percentages and standard errors were not switched. The extremely high standard error for the *qpAdm* estimates of the Iron Age individual buried in northern Europe is due to only one, low coverage genome being used in this group (Martiniano *et al.* 2016).

‘Evidence for European ancestry in a Romano-British individual from Duffield terrace, Yorkshire.’

I think the name of the site is ‘Driffield Terrace’.
Accepted, good catch!

Overall though, an excellent paper, which I thoroughly enjoyed reading. A lot of hard work has been put into this and it shows.

References

Dulias, K., Foody, M.G.B., Justeau, P., Silva, M., Martiniano, R., Oteo-García, G., Fichera, A., Rodrigues, S., Gandini, F., Meynert, A. and Donnelly, K., 2022. Ancient DNA at the edge of the world: Continental immigration and the persistence of Neolithic male lineages in Bronze Age Orkney. Proceedings of the National Academy of Sciences, 119(8), p.e2108001119.

Gilbert, E., O’Reilly, S., Merrigan, M., McGettigan, D., Molloy, A.M., Brody, L.C., Bodmer, W., Hutnik, K., Ennis, S., Lawson, D.J. and Wilson, J.F., 2017. The Irish DNA Atlas: Revealing fine-scale population structure and history within Ireland. Scientific reports, 7(1), pp.1-11.

Patterson, N., Isakov, M., Booth, T., Büster, L., Fischer, C.E., Olalde, I., Ringbauer, H., Akbari, A., Cheronet, O., Bleasdale, M. and Adamski, N., 2022. Large-scale migration into Britain during the Middle to Late Bronze Age. Nature, 601(7894), pp.588-594

Schiffels, S., Haak, W., Paajanen, P., Llamas, B., Popescu, E., Loe, L., Clarke, R., Lyons, A., Mortimer, R., Sayer, D. and Tyler-Smith, C., 2016. Iron age and Anglo-Saxon genomes from East England reveal British migration history. Nature communications, 7(1), pp.1-9.

**Reviewer #2:**

This paper presents newly generated ancient DNA data, from two sites dated to the 4-6th century located in Northern and Southern present-day Scotland. They are considered "Pictish" sites based on their age and location. The new data consists of two whole-genome individuals sequenced (one intermediate and one for aDNA exceptionally high coverage individual with 2.4 and 16.5x average coverage, respectively), and six additional mitogenomes.

The authors apply a high-resolution population genetic analysis to the two individuals with autosomal data, including PCAs based on many present-day Northern and Western Europeans, as well as haplotype sharing analysis, which is cutting edge in aDNA analysis. They find that the two whole genomes cluster close to contemporaneous Northern British samples, which is not a major surprise. There is variability between the two samples and they both cluster seemingly with "Western" British samples (i.e. Walisians and coastal Norther Islanders), even closer than with present-day Eastern Scotlanders which would be closer to the region of the Pictish samples. There is some interesting speculation on why that may be, including a hypothesis that Eastern Scotland received immigration later on. The patterns hold both for classical allele frequency-based and haplotype-sharing methods.

The authors also make a claim that the seven different mtDNA lineages from one Archeological site in Southern Scotland argue against a matrilineal system, a long-standing hypothesis for Pictish society (based on vague textual records).

Regarding the significance of the study, it is notably the first genome-wide data from the Picts. However, as the authors point out themselves, it is not clear how general the results are. Two samples, from two geographically far apart sites, do not allow one to make a conclusion about the general populace of all of Scotland, as there could be population genetic variation, both between and within sites. Nowadays it is a very low sample size, most aDNA studies include dozens or even hundreds of individuals, in particular from more recent times where the number of potential aDNA samples is abundant.

Considering these caveats of low sample size, it is especially striking that two individuals with seemingly well-conserved genome-wide DNA data have been excluded from any autosomal analysis. The reason is suspected cross-contamination from native Americans due to a technical artifact called index hopping (see point A1 below). So the sample size could have been larger, with reasonable monetary effort. That exclusion also raises alarm bells, in terms of data quality and also in terms of potentially biased exclusion of data (see point A1 below).

The population analysis is mostly sound, but there are several oddities in the analysis (see points below). The imputation and IBD part is seemingly cutting edge - but it is effectively a "black box" approach, where algorithms developed for high-quality modern DNA data are applied to aDNA, with very limited verification of the tools and testing for technical covariates (coverage, ancestry, etc.). Thus, it is questionable how well this study can serve as a blueprint for other work - however, I understand that the overall goal here is to screen for qualitative patterns.

Overall, the article is well structured and easy to follow, and the analysis and the interpretation are mostly laid out clearly. The literature and previous studies are generally cited well, at least from an ancient DNA viewpoint (which is my expertise). Previous work on shared segments could be cited more comprehensively, e.g. about relationships between population sizes and segment sharing or expected geographical patterns - to aid discussion of the results.

Below please find detailed comments, first summarize critical comments (A), then major points (B), and then minor suggestions (C) which are not-make-or-break but the authors hopefully find useful.


A: Critical Points (which can be make-or-break pitfalls):

A1) Exclusion of genome-wide data from two samples (LUN001 and LUN003) from any analysis. According do the SI text, LUN003 actually does not show any direct significant evidence of contamination, and the Y-based one for both samples (1 and 3%) is well within the standard limits of aDNA contamination estimates (<10%). The f-statistics-based admixture estimate is highly problematic, as Native American samples are enriched for an ancestry component also high in Steppe-ancestries (e.g. Karitiana are often the top attraction in f-statistics when comparing Steppe ancestries to Neolithic Europeans). In case the two samples are simply higher in Steppe ancestry than the other samples, this admixture test would give significant results, as the assumptions of cladality are broken. A simpler and more robust method would be e.g. a two-way qpADM model with the two putative contaminants as one source, several plausible European ancient individuals as the second source, and other Native Americans as one outgroup.

But given the e.g. low Y chromosome contamination at least showing some basic population genetic analysis (such as PCA) in Supplementary Analysis is clearly warranted. Otherwise, some readers might suspect data has been omitted because it "does not fit or would complicate the story".

Ideally, of course, these data would be actually generated again to be sure - some in the aDNA field might even argue that there is a moral duty to make the most out of the data obtained through destructive sampling.

S1.2 Text. Evidence for index misassignment in LUN001 and LUN003, Lines 102-128:

Thank you, the *qpAdm* test was a good suggestion. It is a more straightforward way to summarise the *D-statistics* and *f4-ratio*. The results of this analysis are displayed in S2 Table. The results are consistent with a true increase in X1 and X2 alleles to the exclusion of any modern Native Americans (set as outgroup), which is consistent with a significant index hopping rate occurring between the samples. X1 and X2 estimates across different ancient Europeans as a second source are consistent with the estimate based on *f4-ratio*, 12-18% for LUN001 and 2-5% for LUN003.

Even if LUN003 has 2-5% contamination from the ancient Native Americans X1 and X2, the PCA shows that low contamination from these highly divergent sources has an important impact on the population genetics statistics.

We also updated S10 Fig PCA and S13-S16 Figs ADMIXTURE plots, now including LUN001 and LUN003.

See also updated parts in S1.3 Text. Genomic analysis on pseudo-haploid sequences, Lines 153-226: LUN001 and LUN003 SNPs calling, PCA and ADMIXTURE have been added.

We agree with the Reviewer that the best thing to do would be to regenerate the data. However, while we would like to generate the data, we have spent significant resources already generating genome-wide data from libraries with ~1% endogenous content. We simply do not have the time or resources to re-generate that data, but by publishing the results we do have (including complete mitochondrial genomes) we maximise the output.

A2) In Table 1, the mtDNA-contamination estimates and CIs are reported in steps of "1.00%". Is that extreme coarseness the consequence of unwanted rounding?

Good point. We replaced them to match the Schmutzi output which is without decimals.

A3) The Haplotype Analysis consists of "black box" approaches, by applying methods developed for modern DNA data without any evaluation of the results. Some potential pitfalls would need investigation in case the methodology should serve as a blueprint for other studies. IBD calling is a highly challenging task even in modern genomes (see e.g. Ralph & Coop 2013, with extensive testing across IBD calling that confirmed rather large IBD detection errors). Of course, the goal of this paper is to screen for broad patterns, but these potential technical pitfalls should be at least acknowledged in the text. As one example, IBD segments of length 1 cm are extremely hard to detect in present-day data - with little power (<<50%) and large false positive rates (see e.g. Seidman et al, 2020) - that plausibly differ between ancestral backgrounds. Some discussion of these points would be certainly in order.

One possible validation is to compare ROH to any ROH estimates based on methods developed, calibrated, and tested on aDNA data (e.g. hapROH, Ringbauer et al 2021). Here you seem to find no ROH>4cM in the two new ancient samples - which is a finding in itself (of relatively large population sizes) - but, if confirmed with hapROH, you could compare on other ancient samples with longer ROH.

That is a good point, we agree with including a discussion on flaws of IBD detection methods. We added a discussion on the potential pitfalls in the Results and discussion, see pages 15-16, Lines 336-359.

We also would like to draw attention to Main Text, Page 24, Line 548 and Supplementary Text S1.6 Page 19, Line 466, with reference to Margaryan *et al.* (2020) who used the 1 cM threshold for their IBD detection.

Concerning our work on the haplotypes, we made a few extra changes:

-Main Text, Page 10, Line 203: we added two extra references to the statement ‘Haplotype-based methods have been shown to outperform conventional unlinked SNP approaches in the detection of population substructure (Lawson *et al.*, 2012; Ralph and Coop, 2013; Al-Asadi *et al.*, 2019)’ to support the choice of using haplotype data for the analysis of ancient European and in particular, populations from the UK, for which allele frequency-based methods have a limited power to detect structure, given their low differentiation.

-We replaced any occurrence of ‘last common ancestor’ with ‘last common genetic ancestor’ which is more accurate when using IBD, and to differentiate it from ‘genealogical ancestor’ (cf. Ralph and Coop (2013)).

A4) Abstract: "Analysis of mitochondrial DNA diversity at the Pictish cemetery of Lundin Links (n = 7) reveals the absence of female endogamy"
The sample size is very small, and the sample is potentially spread out over 130 years, with even non-overlapping RC dates. Without having any "family" or "pedigree" results it is plausible that the 7 individuals are from different families. Generally, mtDNA diversity is very high in most populations - and it is not clear whether a few generations of "female endogamy" can reduce mtDNA diversity (it's hard for women to have a large number of daughters). Thus, I believe this claim is not strongly supported and the language should be tuned down also in the abstract.

That is a good point. See modifications Page 2, Line 44.


B: Major Points:

B1) A PCA built including imputed ancient genomes has the potential for batch effects (e.g. discussed for PC3 in the SI). Given the vast number of present-day genomes, a much more robust way to do this is to construct the PCA based on modern genomes only, and then project the ancient pseudo-haploid data onto it, using a shrinkage correction (as available in the SMARTPCA software used here). This is the standard in the aDNA field, and e.g. Fig. 2 would very likely look very similar - as the present-day variation dominates the sample in any case. Importantly, it would leave much less room for any bias.

We decided that it was needed to expand on the imputation strategy we used, following your comments, and added more to this part. We have added justification concerning the imputation method (see Supplementary Text S1.4, Page 14, Lines 336-340), stating that we followed the imputation pipeline from Margaryan et al. (2020) which used a similar approach as in Gamba *et al.* (2014), Jones *et al.* (2015) and Martiniano *et al.* (2016, 2017), to the only exception that we used GLIMPSE instead of BEAGLE. GLIMPSE has shown better accuracy and efficiency (Rubinacci *et al.*, 2021).
This method, as used in Martiniano *et al.* (2017), was shown to be reliable as the authors empirically proved the imputed genotypes from 2X genomes were 99% accurate compared to the diploid call based on ~15X genomes, except for sites with maf < 5%, which were discarded in our study. Moreover, Hui *et al.* (2020) showed that imputation of very low-coverage genome <0.5X resulted in <97% imputation accuracy.

However, in Martiniano *et al.* (2017) they found that pseudo-haploid bias was stronger than imputation bias as tested using *D-statistics*. They hypothesise bias towards the reference (as shown in Günther and Nettelblad, 2019). The ‘reference bias’ might impact pseudo-haploid genome affinities in different and unpredictable ways, especially when comparing genomes reconstructed from diverse coverage.

The PCAs replicating the PCA including the ancient, imputed genomes, but with the same genomes projected and pseudo-haploidised, are displayed in S24 and S25 Figs. Interpretation and previous discussion on imputation vs. pseudo-haploid bias are included in Text S1.4, Page 16-17, Line 392-406.

B2) L546-L548, SI: Downsampling for IBD "The total number and total length of shared IBD segments were then calculated. To avoid sample size bias, we randomly selected the same number of modern individuals across populations."

One can simply normalize with the number of screened pairs. That way one can make use of all the modern data and does not need to throw away so many individuals.

In order to use all the individuals we gathered for this study, we used bootstrapping. We calculated the number of IBD between an equal subset of individuals per population, resampled and recalculated a 100 times, and then averaged to get the total number of shared IBD or the total length of shared IBD. We explain our method in these sections:

Main text, Page 24, Lines 554-559: ‘To avoid sample size bias, we randomly selected the same number of modern individuals across populations using 100 bootstraps (S1.6 Text).’

Supplementary text S1.6, Page 20, Line 491-503: ‘The total number and total length of shared IBD segments were then calculated. To avoid sample size bias, we randomly selected the same number of modern individuals across populations. Four sets of IBD comparisons were used:

1) modern European populations, based on the mean and 95% confidence interval (CI) using 100 bootstraps based on 44 randomly drawn individuals per modern population (the minimum sample size among the European populations),

2) modern European populations compared to ancient individuals, based on the mean and 95% CI using 100 bootstraps based on 44 randomly drawn individuals per modern population,

3) modern UK populations compared to ancient individuals from Britain, using 17 random individuals per population (defined by county), based on the mean and 95% CI using 100 bootstraps based on 17 randomly drawn individuals per modern UK population (minimum sample size among the UK populations),

4) ancient individuals.’

Using bootstrapping or normalisation over the number of screened pairs are two equally acceptable methods, so we chose to keep the bootstrapping method first used. However, we appreciate that the phrasing is perhaps not so clear. We modify the method in the main text as follows, Page 24, Lines 555-559:

‘To avoid sample size bias, we used a bootstrapping method to compare the total number and length of shared IBD over an equal number of individuals when using modern populations with an unequal number of individuals. We randomly selected an equal number of modern individuals across populations to calculate the total number and length of shared IBD and averaged using 100 replicates with replacement (S1.6 Text).’

B3) L526-L528, SI: "Thus, IBD blocks longer than 1 cM derive from common ancestors living ~50 generations in the past, or ~1,500 years ago"

That is a huge oversimplification. For an insightful primer see e.g. Ralph & Coop 2013.

First, you mean segments ~1cM (longer ones tend to be more recent). Second, other periods contribute to ancestors producing IBD of 1cM too, how much depends on the demographic history. E.g. a bottleneck 3000 years ago still has the potential to contribute very substantially to IBD 1 cm long. Even for a panmictic population, the distribution of recent ancestors contributing to IBD of a given length is very broad (see e.g. Ringbauer et al, 2021, Fig. S14).

That is a very insightful comment. However, we decided to keep IBD ≥ 1 cM because many of them might fall within close to ~50 generations (assuming that some populations in this study underwent a bottleneck and others population size expansion). We assume that we will lose too much relevant information concerning the ancient individual relatedness if we lift the IBD length detection threshold (i.e. see S21 Fig, we observe many individuals with 0 IBD segment ≥ 4 cM).

However, we agree that at least a discussion concerning the different causes impacting IBD block length distribution and coalescence time is a constructive addition here. We added a more nuanced discussion in Text S1.6, Page 19, Lines 473-478.

B4) Data availability" It seems that only the raw bam files will be shared. However, for overall replicability, it will be extremely helpful if the processed data used in the analysis (both pseudo-haploid and especially the imputed data) would be made publicly available too.

We are more than happy to provide the imputed genomes. We have clarified this in the data availability section as part of the submission process.

B5) Fig. S1 is very low resolution. The main figures suffer from low resolution and strong pixelations artifacts too, especially the PCA figure (Fig. 2)

All the figures were generated with 400 dpi. Resolution loss is done on purpose by Plos Genetics to make it easier for the Editor and reviewers to download the file.

Please see the email below:

*Dear Dr Morez,

Thank you for your email.

The compiled PDF compresses the figures to reduce the size of the file and make it easier for the Editor and reviewers to download. Please be assured that, should your manuscript be accepted for publication, our Production team will source the figures from the original files and not the PDF.

Please don't hesitate to get in touch if you have further questions.

Kind regards,  
  
Andrea Szabo
Journal Assistant, Editorial Office Ltd.
On behalf of PLOS Genetics

PLOS |* [*plos.org*](http://plos.org/) *Empowering researchers to transform science
1160 Battery Street, Suite 225, San Francisco, CA 94111

Case Number: 07761800
ref:_00DU0Ifis._5004P22tVae:ref*

B6) One of the two individuals with autosomal data lacks a C14 date (LUN004). Given the relatively low cost of radiocarbon dating (<500 Euro/Dollar), is there any chance to obtain one such date? RC dates can provide critical information and safeguard against unwanted surprises (such as the Neolithic "Picts" mentioned in the text).

This is a very good point so we directly radiocarbon dated LUN004 to 406-542 cal AD (95.4% probability), which falls within the Pictish period. The collagen extraction method and radiocarbon dating details are in Text S1.1, Pages 2-3, Lines 33-51.

We re-generated the Bayesian model for the Lundin Links cemetery and updated S2 Fig and S4 Table and Table 1.

B7) The UDG-treated two whole-genome sequences have very little damage (<<5%, even on the end, see Fig. S3), as expected. It is not needed to restrict the analysis to transversions only then (and effectively never done in aDNA practice in these cases). But here this removal of large amounts of data is done for pseudo-haploid analysis and as input for imputation (see below). That throws away large amounts of data without any real need to.

In the pseudo-haploid analyses, we decided to remove transitions for BAL003 and LUN004 to standardise comparison with other ancient genomes that were not UDG-treated and for which transitions were excluded. Otherwise, it can potentially bias the result, especially of ADMIXTURE and *f-statistics*.

For imputation, however, we removed the transitions only on the first 5 bases in 5’ and 3’ (by decreasing the Phred score to 2) (following Martiniano *et al.* (2016) and Margaryan *et al.* (2020)) (see Text S1.4, Page 15, Lines 358-361), since BAL003 and LUN004 libraries evidence slight deamination occurring in the first 5 bases 5’ and 3’ (S3 Fig).

B8) L147-148 "we assigned LUN004 to R1b-L52 (Table 1, S6 Table), but without additional resolution to determine whether this sample carries the R1b-P312/S116" R1b-P312"
A sample with >2x average whole genome (!) coverage should most likely be resolvable. Is it really the case that all of the several equivalent markers (and dozens of potential downstream mutations along each major branch) are not covered at all?

We used the relaxed parameter -m ‘no-filter’ from pathPhynder and could find one derived allele at R1b1a1b1a1a2c1a1a, supported by a single read (G to A). See updated S6 Table.

We modified accordingly the Results and Discussion (Page 8, Line 168) and Material and Methods (Page 21, Lines 469/470).

B9) L204-L206: "However, due to allele frequency bias between the two imputed datasets, likely skewing allele frequency-based analyses (S1.4 Text, S8, and S9 Figs), we refrained from calculating D-statistics to investigate this signal further"

Why not calculate the f-statistics on pseudo-haploid data then? f-statistics are extremely robust and their power anyways "tops out" with higher coverage (due to linkage - above 100k pseudo-haploid SNPs usually more SNPs != more power).

We calculated *f-statistics* on the pseudo-haploid data. We used outgroup *f3-statistics* on the pseudo-haploid data to estimate the shared drift between Picts and modern Orcadians and between Iron and Viking Age Orcadians and modern Orcadians, which confirms high shared drift in Orkney for the past 2,000 years. The results are reported Page 11, Lines 228-236. We also modified Material and Methods Page 22, Lines 510-513 to integrate this new dataset into the method.

An intuitive test would be *D(Orkney_IA,modern Orcadian; Picts/England_IA/England_Roman, YRI)*, to test whether ancient and modern Orcadians form a clade to the exclusion of contemporary people living in the British Isles. However, we know this test design is subject to bias caused by a greater chance to find an excess similarity between pseudo-haploid genomes and the reference genome (Günther and Nettelblad, 2019), which will falsely inflate the similarity between the Picts and Orkney_IA/VA.

C: Minor Points:

C1) L407-411: Imputation
"For non-damaged repaired genomes, genotypes potentially resulting from deamination were set as missing, replacing 0/1 and 1/1 genotypes by ‘./.’ where the reference allele is C or G, and the alternate is T or A or replacing 0/0 and 0/1 genotypes by ‘./.’ where the reference allele is T or A and the alternate is C or G."

Is this not a potential bias, as only derived alleles are being masked out? Imputation will probably not recover all of them, or at least with lower certainty than in cases where there are reference alleles. The way to go would be to call genotype likelihoods with aDNA damage aware likelihoods - e.g. ATLAS or snpAD.

This is a good observation. This method might increase the error rate of the imputation but not population affinities: there is no reason to believe that the error rate will change allele frequency towards a certain ancestry. Moreover, in the PCA for example, there is no bias associated with UDG/non-UDG genome (transition kept/transition removed).

Importantly, given the low remaining damage after the USER enzyme treatment, restricting to only transversion SNPs seems overly conservative in any case (see point B8 above).

The genomes undergoing this filtering (i.e., replacing the derived allele at transition sites with a missing genotype) are only the *non-damaged repaired* genomes. Your suggestion on calling genomes using ATLAS or snpAD is indeed a good consideration but we chose to follow the already published pipeline from Martiniano *et al.* (2016) and Margaryan *et al.* (2020). In particular, Martiniano *et al.* 2016 showed that imputation bias in the pipeline we followed is less important than pseudo-haploid bias.

C2) L420-L443: You investigate bias on PC3 - showing that there is a coverage-correlated bias for both the Margaryan and your imputation. It would be good to test and mention that on PC1/PC2 there is none - as these PCs are centrally featured in the main text and figures. In any case, you could simply project pseudo-haploid ancient data to avoid these evident interactions of PCA and imputation (see point B1 above).

The coverage-correlated bias observed on PC3 is not present on PC1 and PC2 as shown by S9 Fig and the correlation tests described in SI Page 16, Line 387-391. This is an extra confirmation that we can rely on the results and draw informative conclusions based on PC1 and PC2.

C3) L181: "To make use of the additional power provided by linkage disequilibrium"
That's an anal technical comment: Haplotype analysis does not need any LD - there can be shared long haplotypes even in a population without any substantial LD. So you really want to say: "the additional power provided by haplotype information"

That is absolutely correct. We modified this sentence.

C4) B3) L565, SI: The "quality control" section first explains some heuristics of the length-cutoff - and then reports that patterns qualitatively agree with present-day IBD patterns. Titling such a section as "quality control" seems ambitious.

We renamed it ‘IBD distribution across time and space’.

References:
- Ralph, Peter, and Graham Coop. "The geography of recent genetic ancestry across Europe." PLoS biology 11.5 (2013): e1001555.

- Seidman, Daniel N., et al. "Rapid, phase-free detection of long identity-by-descent segments enables effective relationship classification." The American Journal of Human Genetics 106.4 (2020): 453-466.

- Ringbauer, Harald, John Novembre, and Matthias Steinrücken. "Parental relatedness through time revealed by runs of homozygosity in ancient DNA." Nature communications 12.1 (2021): 1-11.

**Reviewer #3:**

A review of Morez et al, Early medieval Pictish genomes reveal fine-scale relatedness in the UK.

The authors present a detailed examination of two Pictish genomes, using an array of tools and methods from the literature. This is a well written article that undertakes an informative and competent analysis of their data, and deserves to be published somewhere. They discuss interesting topics such as the potential for these societies to be matrilineal, and exactly who these people were.

However, the main sources of evidence are the amount of IBD that these two genomes share with other genomes, as well as a detailed discussion of seven mtDNA genomes. This is an interesting but slightly under-developed analysis - we could do the same for any data, and typically do not report the results in such detail, because it is fundamentally just a property of the data, and not enough to overcome the lack of sample diversity. The other methodology deployed is competent and appropriate but lacks detailed insight. The overall result then is somewhat anecdotal at the level of its implications for the diversity of historical Pictish cultural groups. Whilst the conclusions drawn are careful, and the topics discussed important, I therefore don't find this paper to be compelling enough for the highest tier of journal.

Technically I think this is a good paper without any substantial issues. The main suggestion, if the authors think it valuable and indeed possible, would be to try to find tools that are specifically designed to formalise the IBD and mt patterns into a model. Can we do any formal statistical testing for the mt results based on no-sex-bias assumptions? Counting IBD is something, but it is not e.g. LD-based admixture dating, which would really have helped say something new about the past. What does the distribution of IBD lengths tell us about the dates for which populations were in contact? etc. But maybe it is better to simply wait for more data to address these questions.

We appreciate the view of Reviewer 3, but kindly disagree in that, while further work and alternate methods will improve our characterization of the Picts, our present research provides an important first step in understanding these enigmatic people. Thanks to the imputed low and high(er) coverage ancient genomes, the IBD analysis provides additional insights only possible due to the amount of data we have for these two individuals, which is not commonly achieved in aDNA studies. We agree that more data would provide additional insights but, as each shared IBD segment represents a genetic ancestor, our IBD analysis can reach as far back as tens of generations (i.e. hundreds of years ago), so they are a sample of the broader population history and not just the history of the individuals. The suggestion to use models to test our hypotheses concerning for example mtDNA-based evidence of female mobility is very promising. However, as Reviewer 3 suggested, we agree that more data would be beneficial before attempting this kind of approach. Thus, based on the positive opinions of Reviewers' 1 and 2, we maintain that it is important to disseminate our findings in such a widely read journal as Plos Genetics.

**References**

Al-Asadi, H. et al. (2019) ‘Estimating recent migration and population-size surfaces’, PLOS Genetics. Edited by M. DeGiorgio, 15(1), p. e1007908. Available at: https://doi.org/10.1371/journal.pgen.1007908.

Byrne, R.P. et al. (2018) ‘Insular Celtic population structure and genomic footprints of migration’, PLoS Genetics, 14(1), pp. 1–22. Available at: https://doi.org/10.1371/journal.pgen.1007152.

Gamba, C. et al. (2014) ‘Genome flux and stasis in a five millennium transect of European prehistory’, Nature Communications, 5(1), pp. 1–9. Available at: https://doi.org/10.1038/ncomms6257.

Gilbert, E. et al. (2019) ‘The genetic landscape of Scotland and the Isles’, Proceedings of the National Academy of Sciences, p. 201904761. Available at: https://doi.org/10.1073/PNAS.1904761116.

Gretzinger, J. et al. (2022) ‘The Anglo-Saxon migration and the formation of the early English gene pool’, Nature, 610(7930), pp. 112–119. Available at: https://doi.org/10.1038/s41586-022-05247-2.

Günther, T. and Nettelblad, C. (2019) ‘The presence and impact of reference bias on population genomic studies of prehistoric human populations’, PLoS Genetics, 15(7). Available at: https://doi.org/10.1371/journal.pgen.1008302.

Hui, R. et al. (2020) ‘Evaluating genotype imputation pipeline for ultra-low coverage ancient genomes’, Scientific Reports, 10(1), p. 18542. Available at: https://doi.org/10.1038/s41598-020-75387-w.

Jones, E.R. et al. (2015) ‘Upper Palaeolithic genomes reveal deep roots of modern Eurasians’, Nature Communications, 6(1), pp. 1–8. Available at: https://doi.org/10.1038/ncomms9912.

Lawson, D.J. et al. (2012) ‘Inference of Population Structure using Dense Haplotype Data’, PLOS Genetics, 8(1), p. e1002453. Available at: https://doi.org/10.1371/journal.pgen.1002453.

Lawson, D.J., Dorp, L. van and Falush, D. (2018) ‘A tutorial on how not to over-interpret STRUCTURE and ADMIXTURE bar plots’, Nature Communications, 9(1), pp. 1–11. Available at: https://doi.org/10.1038/s41467-018-05257-7.

Margaryan, A. et al. (2020) ‘Population genomics of the Viking world’, Nature, 585(7825), p. 390. Available at: https://doi.org/10.1038/s41586-020-2688-8.

Martiniano, R. et al. (2016) ‘Genomic signals of migration and continuity in Britain before the Anglo-Saxons’, Nature Communications, 7, pp. 1–8. Available at: https://doi.org/10.1038/ncomms10326.

Martiniano, R. et al. (2017) ‘The population genomics of archaeological transition in west Iberia: Investigation of ancient substructure using imputation and haplotype-based methods’, PLoS Genetics, 13(7), pp. 1–24. Available at: https://doi.org/10.1371/journal.pgen.1006852.

Patterson, N. et al. (2021) ‘Large-scale migration into Britain during the Middle to Late Bronze Age’, Nature, pp. 1–14. Available at: https://doi.org/10.1038/s41586-021-04287-4.

Ralph, P. and Coop, G. (2013) ‘The Geography of Recent Genetic Ancestry across Europe’, PLOS Biology, 11(5), p. e1001555. Available at: https://doi.org/10.1371/journal.pbio.1001555.

Rubinacci, S. et al. (2021) ‘Efficient phasing and imputation of low-coverage sequencing data using large reference panels’, Nature Genetics, 53(1), pp. 120–126. Available at: https://doi.org/10.1038/s41588-020-00756-0.

Schiffels, S. et al. (2016) ‘Iron Age and Anglo-Saxon genomes from East England reveal British migration history’, Nature Communications, 7(1), pp. 1–9. Available at: https://doi.org/10.1038/ncomms10408.

Wainwright, F.T. (1955) The Problem of the Picts. Edited by F.T. Wainwright. Nelson.
